# Synthesis, chirality-dependent conformational and biological properties of siRNAs containing 5′-(*R*)- and 5′-(*S*)-*C*-methyl-guanosine

**DOI:** 10.1093/nar/gkaa750

**Published:** 2020-09-29

**Authors:** Atsushi Mikami, Namrata Erande, Shigeo Matsuda, Alexander Kel’in, Lauren Blair Woods, Tyler Chickering, Pradeep S Pallan, Mark K Schlegel, Ivan Zlatev, Martin Egli, Muthiah Manoharan

**Affiliations:** Alnylam Pharmaceuticals, 675 West Kendall Street, Cambridge, Massachusetts 02142, USA; Alnylam Pharmaceuticals, 675 West Kendall Street, Cambridge, Massachusetts 02142, USA; Alnylam Pharmaceuticals, 675 West Kendall Street, Cambridge, Massachusetts 02142, USA; Alnylam Pharmaceuticals, 675 West Kendall Street, Cambridge, Massachusetts 02142, USA; Alnylam Pharmaceuticals, 675 West Kendall Street, Cambridge, Massachusetts 02142, USA; Alnylam Pharmaceuticals, 675 West Kendall Street, Cambridge, Massachusetts 02142, USA; Department of Biochemistry Vanderbilt University, School of Medicine Nashville, TN 37232, USA; Alnylam Pharmaceuticals, 675 West Kendall Street, Cambridge, Massachusetts 02142, USA; Alnylam Pharmaceuticals, 675 West Kendall Street, Cambridge, Massachusetts 02142, USA; Department of Biochemistry Vanderbilt University, School of Medicine Nashville, TN 37232, USA; Alnylam Pharmaceuticals, 675 West Kendall Street, Cambridge, Massachusetts 02142, USA

## Abstract

Various chemical modifications have been identified that enhance potency of small interfering RNAs (siRNAs) and that reduce off-target effects, immune stimulation, and toxicities of metabolites of these therapeutic agents. We previously described 5′-*C*-methyl pyrimidine nucleotides also modified at the 2′ position of the sugar. Here, we describe the synthesis of 2′-position unmodified 5′-(*R*)- and 5′-(*S*)-*C*-methyl guanosine and evaluation of these nucleotides in the context of siRNA. The (*R*) isomer provided protection from 5′ exonuclease and the (*S*) isomer provided protection from 3′ exonuclease in the context of a terminally modified oligonucleotide. siRNA potency was maintained when these modifications were incorporated at the tested positions of sense and antisense strands. Moreover, the corresponding 5′ triphosphates were not substrates for mitochondrial DNA polymerase. Models generated based on crystal structures of 5′ and 3′ exonuclease oligonucleotide complexes with 5′-(*R*)- and 5′-(*S*)-*C*-methyl substituents attached to the 5′- and 3′-terminal nucleotides, respectively, provided insight into the origins of the observed protections. Structural properties of 5′-(*R*)-*C*-methyl guanosine incorporated into an RNA octamer were analysed by X-ray crystallography, and the structure explains the loss in duplex thermal stability for the (*R*) isomer compared with the (*S*) isomer. Finally, the effect of 5′-*C*-methylation on endoribonuclease activity has been explained.

## INTRODUCTION

**Figure 1.**
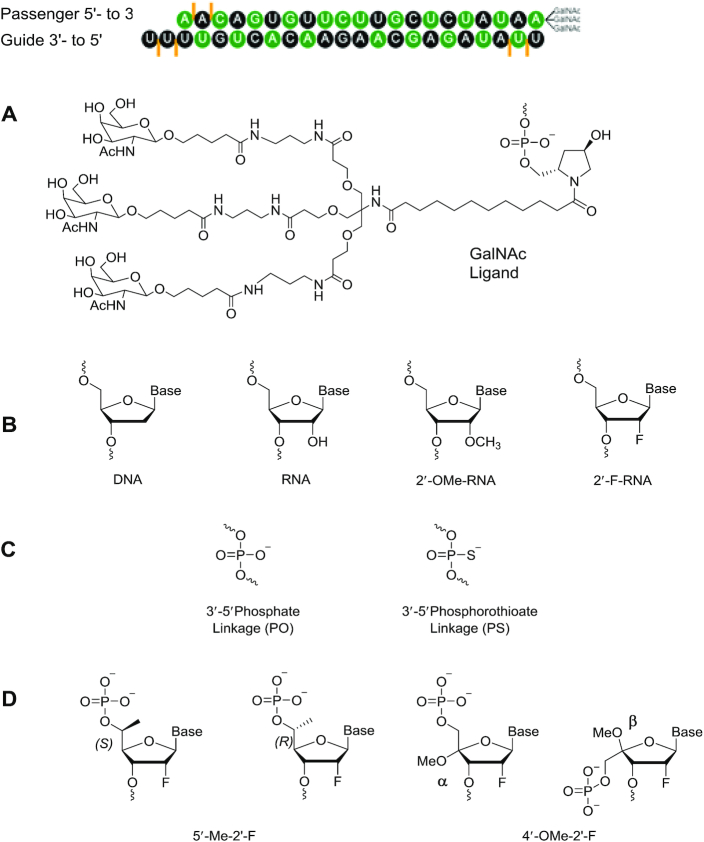
(**A**) Structural illustration of a chemically modified siRNA-GalNAc conjugate duplex. Black: 2′-*O*-methyl (2′-OMe), green: 2′-deoxy-2′-fluoro (2′-F), orange lines: phosphorothioate linkage (PS). (**B**) Structures of the native nucleotides and standard modified nucleotides mentioned in this study, including 2′-OMe and 2′-F. (**C**) Backbone chemistries include both natural phosphate (PO) and phosphorothioate (PS; orange lines in A). (**D**) Representative 4′- and 5′ modifications studied in our laboratories with 2′-modifications.

Chemical modifications impart drug-like properties and have made possible the recent clinical successes of oligonucleotide therapeutics. The first FDA-approved agent to act through the RNA interference (RNAi) pathway, patisiran (ONPATTRO, Alnylam Pharmaceuticals), was approved by regulatory agencies in the USA and Europe in 2018 and in Japan in 2019 for the treatment of hereditary transthyretin amyloidosis polyneuropathy caused by the deposition of mutated amyloid protein in multiple organs of the body (1). Some of the nucleotides in patisiran are chemically altered at the 2′ position of the ribose sugar with 2′-*O*-methyl (2′-OMe). This modification increases binding affinity of the siRNA for the complementary mRNA by stabilizing the sugar in the C3′-*endo* conformation (2–4). The 2′-OMe modification and other types of 2′-ribosugar chemical modifications also inhibit nuclease cleavage of the siRNA and reduce immunostimulatory properties (3,4). Patisiran is packaged in a multi-component lipid nanoparticle (LNP), and the LNP provides necessary nuclease protection for the 2′-unmodified nucleotides in addition to providing molecular trafficking to liver cells by an Apo-E binding mechanism (1). The FDA recently approved a second RNAi drug, givosiran (GIVLAARI, Alnylam Pharmaceuticals, 2019, https://www.fda.gov/news-events/press-announcements/fda-approves-first-treatment-inherited-rare-disease), for the treatment of acute hepatic porphyria (5,6). Givosiran is also the first approved oligonucleotide-based therapeutic to be conjugated to trivalent *N*-acetylgalactosamine (GalNAc), a ligand that results in hepatocyte-specific delivery (7). Except for the chemical modifications and the terminal ligand, this duplex is not protected from nucleases; it is not formulated with LNP. The clinically approved siRNAs and those currently in phase III clinical trials (5,8–15) contain some residues modified at the 2′ position of the ribosugar when they are formulated with LNP or, in the case of GalNAc conjugates are fully modified with 2′-OMe or 2′-deoxy-2′-fluoro (2′-F) (16–18) as well as phosphorothioate (PS) linkages (Figure 1). PS, 2′-OMe and 2′-F are tolerated as single modifications in all individual positions of an siRNA, but when all residues are modified with 2′-OMe, the siRNAs are inactive, and siRNAs fully modified with 2′-F have reduced activity compared to the unmodified siRNAs in cell-based assays (19).

Although chemical modifications can mitigate certain undesired effects of oligonucleotide therapeutics, chemically modified nucleosides released due to metabolism of the oligonucleotide can potentially be phosphorylated by cellular kinases and interfere with cellular polymerases. For example, the 5′-phosphorylated 2′-F monomers are recognized as substrates by human RNA and DNA polymerases at high concentrations (17,20–22). Some 2′-F- and PS-modified oligonucleotides alter levels of RNA and protein markers for hepatotoxicity, liver necrosis, and apoptosis, but not all the 2′-F-modified oligonucleotides are toxic (23). It appears that the combination of 2′-F with PS may be the contributing factor for toxicity. Furthermore, oligonucleotides containing 2′-F nucleosides are significantly less nuclease resistant than oligonucleotides with other 2′ modifications (24).

To overcome such limitations and to further improve the pharmacological properties of oligonucleotide therapeutics, effects of modifications of sugar positions other than the 2′ position have been investigated (25–27). Both C4′ and C5′ epimers (Figure 1) have remarkable enzymatic stability due to steric conflicts with the two conserved metal ions (typically Mg^2+^) in the nuclease active site (25,27). Such steric interference may also hamper polymerase activity. siRNAs modified with 2′-F or 2′-OMe in combination with 4′-*C*α-*O*-methyl-uridine afford excellent protection against attack by nucleases and exhibit thermal stability similar to the unmodified duplex and gene silencing efficiency comparable to siRNA modified with only 2′-F (25). In contrast, 2′-F, 4′-*C*β-*O*-methyl-uridine with an inverted stereocenter at C4′ strongly destabilizes the RNA duplex (25,28).

**Figure 2.**
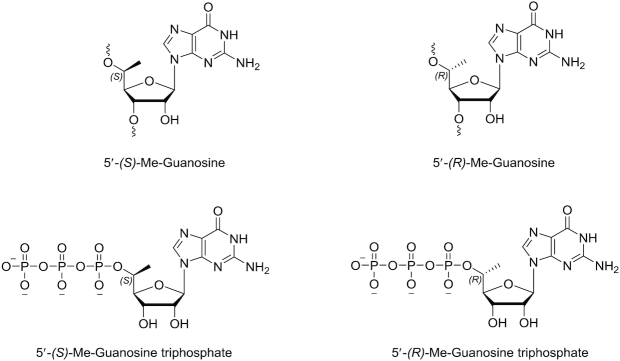
Structures of the 5′-*C*-methyl guanosine ribonucleosides and their triphosphates studied in the present work.

A clinically advanced GalNAc-conjugated siRNA for the treatment of hypercholesterolemia is inclisiran, which targets the *PCSK9* gene. It has a single unmodified deoxynucleotide in the sense strand and has shown efficacy in thousands of patients. Many unformulated siRNA candidates are being tested have unmodified RNA nucleotides, but none have progressed in clinical studies, presumably due to poor metabolic stability. To maintain the natural RNA nucleotides in strategic positions along with improved biostability, we decided to evaluate 5′-modified RNA nucleotides, beginning first with guanosine (Figure 2).

Shah *et al.* recently synthesized and characterized 5′-*C*-methyl guanosine deoxynucleotides (29), and our laboratory has synthesized a series of chirally pure 5′-(*R*)- and 5′-(*S*)-*C*-methyl pyrimidines carrying either 2′ modifications or 2′-deoxy (27); however, the effect of enantiomerically pure 5′-(*R*)- and 5′-(*S*)-*C*-methyl substitutions in combination with 2′-hydroxy group in the context of siRNA is unexplored. In the context of RNA duplexes, these modifications generally decrease thermal stability, and both isomers increased stability in the presence of 3′ exonucleases compared to unmethylated analogues (27). There is substantial structural similarity between (*S*) epimers of 5′-*C*-methyl nucleosides and corresponding unmethylated counterparts, although the methylated nucleosides have a higher percentage of C3′-*endo* sugar puckering regardless of 2′-ribo substitution than the unmethylated nucleosides. In contrast, the (*R*) isomers preferentially exhibit a *syn* nucleobase orientation and C2′-*endo* sugar puckering. Hence, in all cases, the (*R*) isomer caused greater destabilization than the (*S*) isomer. The spatial orientation of the 5′-*C*-methyl also influences duplex stability (27). The effects of 5′-substitution in combination with locked nucleic acid (LNA) have been investigated by Seth *et al.* (30). LNAs have extremely high affinity for complementary RNA and are stable in serum, but LNA residues at certain positions of siRNAs compromise activity (31). The introduction of 5′-*C*-methyl group in the (*S*) configuration in LNA results in high-affinity recognition of complementary nucleic acids; in contrast, the 5′-(*R*)-*C*-methyl group was destabilizing (30). The 5′-(*R*)-*C*-methyl-LNA oligomer had higher stability in a 3′-exonuclease digestion assay than the (*S*)-isomer-modified strand. In animal experiments, antisense oligonucleotides modified with 5′-(*S*)-*C*-methyl and LNA had slightly lower potency than sequence-matched LNA antisense oligonucleotides but improved therapeutic profiles (30).

Beigelman's group used a glycosylation approach to synthesize 5′-(*R*)- and 5′-(*S*)-*C*-methyl guanosine and adenosine nucleosides and analysed their influence on ribozyme activity (32,33). As one goal of this effort was to increase the metabolic stability of siRNAs and guanosine provides the most metabolic stability of the nucleobases (34), we first focused on 5′-(*R*)- and 5′-(*S*)-*C*-methyl-substituted guanosine. Here, we report the synthesis and structural characterization of enantiomerically pure 5′-(*R*)- and 5′-(*S*)-*C*-methyl-substituted guanosine 3′-phosphoramidites as well as their solid supports and 5′-triphosphates. Since the direct Grignard methylation from guanosine had not been attempted, we explored this route. We performed Mosher ester analysis for the configurational analyses of both 5′-(*R*)- and 5′-(*S*) enantiomers, and the sugar conformations were analysed using ^1^H NMR. We have evaluated the impacts of chirality of 5′-*C*-methyl ribonucleotides on resistance to cleavage by 3′-exonuclease snake venom phosphodiesterase (SVPD) and by 5′-exonuclease phosphodiesterase-II (PDE-II), and the results ware explained using molecular modelling of modified oligonucleotides in the context of the crystal structure of an exonuclease. To explore the impact of metabolites of siRNAs modified with these residues on mitochondrial polymerases, we also studied the recognition of 5′-(*R*)- and 5′-(*S*)-*C*-methyl-guanosine 5′-triphosphates by the mitochondrial RNA polymerase (POLRMT) and the DNA polymerase subunit γ (POLG). Unlike the 2′-F monomers (17,20–22), the 5′-*C*-methyl-substituted guanosine monomers were not polymerase substrates. These modifications were also well tolerated at the tested positions in siRNAs when RNAi activity was investigated in a cell-based assay. The conformational features of 5′-(*R*)-*C*-methyl guanosine incorporated into an RNA octamer was also analysed by X-ray crystallography allowing us to rationalize chiral dependencies of biophysical and biological properties of the 5′-*C*-methyl-substituted siRNAs.

## MATERIALS AND METHODS

### Synthetic procedures and compound characterization

#### General conditions

TLC was performed on Merck silica gel 60 plates coated with F254. Compounds were visualized under UV light (254 nm) or after spraying with the *p*-anisaldehyde staining solution followed by heating. Flash column chromatography was performed using a Teledyne ISCO Combi Flash system with pre-packed RediSep Teledyne ISCO silica gel cartridges. All moisture-sensitive reactions were carried out under anhydrous conditions using dry glassware, anhydrous solvents, and argon atmosphere. All commercially available reagents and solvents were purchased from Sigma-Aldrich unless otherwise stated and were used as received. ESI-MS spectra were recorded on a Waters Qtof Premier instrument using the direct flow injection mode. ^1^H NMR spectra were recorded at 400 or 500 MHz. ^13^C NMR spectra were recorded at 101 or 126 MHz. ^31^P NMR spectra were recorded at 202 MHz. Chemical shifts are given in ppm referenced to the solvent residual peak (DMSO-*d*_6_ – ^1^H: δ at 2.50 ppm and ^13^C δ at 39.5 ppm; CDCl_3_ – ^1^H: δ at 7.26 ppm and ^13^C δ at 77.2 ppm). Coupling constants are given in Hertz. Signal splitting patterns are described as singlet (s), doublet (d), triplet (t) or multiplet (m).

**Scheme 1.**
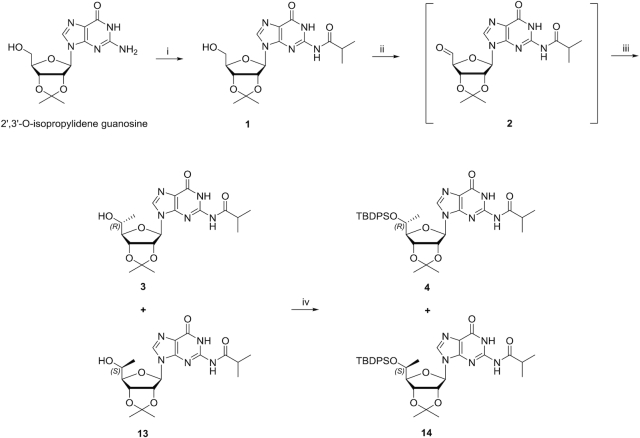
Reagents and conditions: (i) 1) TMSCl/pyridine, 0°C to room temperature, 1 h. 2) isobutyrylchloride/pyridine, 0°C to room temperature, overnight; 93%. (ii) Dess–Martin periodinane/DCM, 0°C to room temperature, 2 h. (iii) MeMgBr/DCM, 0°C, 30 min; 2 steps 33% as a mixture of **3** and **13**. (iv) TBDPSCl, imidazole/DMF, room temperature, overnight; 17% for **4** and 23% for **14**.

**Scheme 2.**
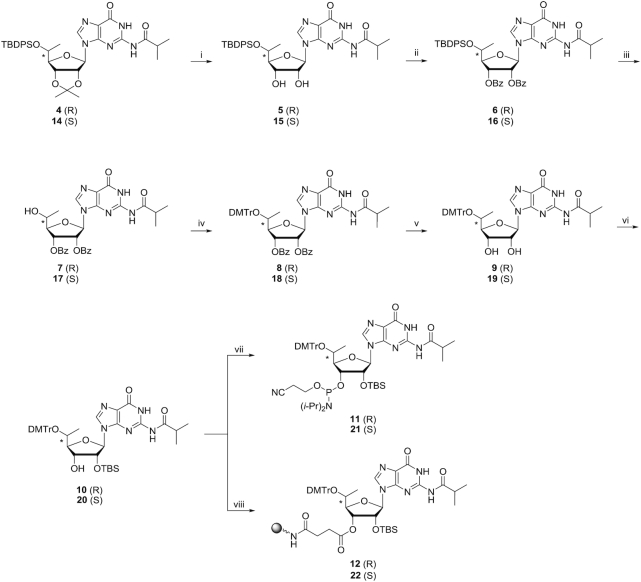
Reagents and conditions: (i) 80% aq. TFA/DCM, 0°C to room temperature, overnight; 83% for **5**, 70% for **15**. (ii) Benzoic anhydride, DMAP/pyridine, room temperature, overnight; 99% for **6** and for **16**. (iii) TBAF/THF, room temperature, overnight; 87% for **7**, 94% for **17**. (iv) DMTrCl, AgNO_3_/THF/pyridine (THF/pyridine, 4:1, v/v), room temperature, overnight; 98% for **8**, 98% for **18**. (v) NaOH/THF/MeOH (THF/MeOH, 1:1, v/v), 0°C, 30 min; 96% for **9** and for **19**. (vi) TBSCl, AgNO_3_/THF/pyridine (THF/pyridine, 4:1, v/v), room temperature, overnight; 50% for **10**, 53% for **20**. (vii) *i*-Pr_2_NP(Cl)O(CH_2_)_2_CN, DIPEA, 1-methylimidazole/DCM, room temperature, 2 h; 88% for **11**, 65% for **21**. (viii) 1) Succinic anhydride, DMAP/DCM, room temperature, overnight. 2) LCAA-CPG (pore size 500 Å, 171 μmol/g), HBTU, DIPEA/DMF, room temperature, overnight. 3) Acetic anhydride/pyridine, room temperature, overnight; 70 μmol/g loading for **12**, 97 μmol/g for **22**.

Synthesis of (*R*)- and (*S*)-Isomers of 5*′-C-*methyl-guanosine are shown in Schemes 1 and 2.

#### Synthesis of Compound 1

Compound **1** was synthesized from 2′, 3′-*O*-isopropylideneguanosine as previously reported (35–37).

#### Synthesis of compound 2

To a solution of compound **1** (34.0 g, 86.4 mmol) in DCM (1 L) at 0°C was added Dess–Martin periodinane (45.8 g, 108.0 mmol), and the mixture was stirred at room temperature for 2 h. The reaction mixture was then added to a solution of 5% sodium thiosulfate (aq.) (250 ml) and saturated NaHCO_3_ (aq.) (250 ml) at 0°C, and the mixture was stirred at room temperature for 20 min. The mixture was washed with brine and dried over Na_2_SO_4_. The organic phase was evaporated to dryness and used for next reaction without further purification.

#### Syntheses of compounds 3 and 13

To a solution of crude compound **2** in DCM (1 l) at 0°C was added MeMgBr (3 M in THF, 86 ml, 258.0 mmol), and the mixture was stirred at room temperature. After 30 min, saturated ammonium chloride (aq.) (500 ml) was added. The mixture was washed with brine and dried over Na_2_SO_4_. The organic phase was evaporated to dryness and purified by silica gel column chromatography (DCM/MeOH, 97.5:2.5, v/v). The mixture of compounds **3** and **13**, which was a white solid, was obtained in 33% yield (11.5 g). ^1^H NMR compound **3** (400 MHz, DMSO-*d*_6_) δH: 1.01 (3H, d, *J* = 6.4 Hz), 1.12 (6H, d, *J* = 6.8 Hz), 1.32 (3H, s), 1.51 (3H, s), 2.77 (1H, m), 3.65 (1H, m), 3.86 (1H, dd, *J* = 5.6, 2.9 Hz), 5.06 (2H, m), 5.24 (1H, dd, *J* = 6.4, 2.9 Hz), 5.98 (1H, d, *J* = 2.9 Hz), 8.23 (1H, s), 11.59 (1H, s), 12.11 (1H, s). ^13^C NMR compound **3** (126 MHz, DMSO-*d*_6_) δC: 180.17, 154.77, 148.38, 148.20, 137.85, 120.08, 113.15, 89.95, 88.26, 83.40, 80.28, 65.92, 34.72, 27.00, 25.18, 19.83 and 18.85. HRMS calc. for compound **3**, C_18_H_26_N_5_O_6_ [M+H] ^+^: 408.1883; found: 408.1889. ^1^H NMR compound **13** (400 MHz, DMSO-*d*_6_) δH: 1.07 (3H, d, *J* = 6.4 Hz), 1.12 (6H, d, *J* = 6.8 Hz), 1.31 (3H, s), 1.52 (3H, s), 2.78 (1H, m), 3.77 (1H, m), 3.95 (1H, m), 4.97 (1H, dd, *J* = 6.3, 3.3 Hz), 5.05 (1H, d, *J* = 4.7 Hz), 5.15 (1H, dd, *J* = 6.3, 3.0 Hz), 6.01 (1H, d, *J* = 3.0 Hz), 8.31 (1H, s), 11.57 (1H, s), 12.11 (1H, s). ^13^C NMR compound **13** (126 MHz, DMSO-*d*_6_) δC: 180.16, 154.80, 148.33, 148.16, 138.01, 120.08, 113.19, 89.66, 88.39, 83.81, 81.05, 66.28, 34.72, 27.17, 25.27, 19.16, 18.89 and 18.85. HRMS calc. for compound **13**, C_18_H_26_N_5_O_6_ [M+H]^+^: 408.1883; found: 408.1889.

#### Syntheses and separation of compounds 4 and 14

To a solution of compounds **3** and **13** (11.5 g, 28.0 mmol) in DMF (300 ml) were added *tert-*butyldiphenylsilylchloride (7.3 ml, 28.0 mmol) and imidazole (3.8 g, 56.0 mmol), and the mixture was stirred at room temperature overnight. The solvent was evaporated to dryness and dissolved into AcOEt, washed with saturated NaHCO_3_ (aq.) and brine, and dried over Na_2_SO_4_. The organic phase was evaporated to dryness and purified by silica gel column chromatography (hexanes/AcOEt/MeOH, 30:66:4, v/v/v). Compound **4** was isolated in 17% yield (3.1 g), and compound **14** was isolated in 23% yield (4.2 g), both as white solids. ^1^H NMR compound **4** (400 MHz, DMSO-*d*_6_) δH: 0.85 (1H, d, *J* = 5.5 Hz), 0.96 (9H, s), 1.11 (6H, m), 1.35 (3H, s), 1.51 (3H, s), 2.75 (1H, m), 3.87 (2H, m), 5.13 (1H, dd, *J* = 6.8, 3.5 Hz), 5.41 (1H, dd, *J* = 6.7, 3.1 Hz), 5.95 (1H, d, *J* = 3.1 Hz), 7.30–7.33 (2H, m), 7.40–7.45 (4H, m), 7.53–7.55 (2H, m), 7.60–7.62 (2H, m), 8.08 (1H, s), 11.57, (1H, s), 12.10 (1H, s). ^13^C NMR compound **4** (126 MHz, DMSO-*d*_6_) δC: 180.10, 154.69, 148.41, 148.19, 137.86, 135.36, 135.23, 133.44, 132.98, 129.83, 129.80, 127.69, 127.59, 120.21, 113.99, 88.25, 87.05, 82.25, 79.73, 68.39, 34.71, 27.05, 26.66, 25.41, 19.29, 18.83, 18.78, 18.71. HRMS calc. for compound **4**, C_34_H_44_N_5_O_6_Si [M+H]^+^: 646.3061; found: 646.3057. ^1^H NMR compound **14**, 40.0 MHz, DMSO-*d*_6_) δH: 0.86 (9H, s), 1.03 (3H, d, *J* = 6.0 Hz), 1.10 (6H, m), 1.31 (3H, s), 1.53 (3H, s), 2.70 (1H, m), 3.86 (1H, m), 3.94 (1H, dd, *J* = 7.9, 4.0 Hz), 5.17 (2H, m), 6.13 (1H, d, *J* = 1.6 Hz), 7.19 (2H, m), 7.29–7.39 (6H, m), 7.45 (2H, m), 8.10 (1H, s), 11.24 (1H, s), 12.03 (1H, s). ^13^C NMR compound **14** (126 MHz, DMSO-*d*_6_) δC: 179.88, 154.72, 147.64, 147.60, 138.75, 135.13, 135.09, 133.83, 132.88, 129.54, 129.47, 127.42, 127.24, 120.72, 113.29, 91.33, 87.68, 83.81, 80.48, 70.01, 34.70, 27.14, 26.60, 25.53, 19.04, 18.79, 18.57. HRMS calc. for compound **14**, C_34_H_44_N_5_O_6_Si [M+H]^+^: 646.3061; found: 646.3069.

#### Synthesis of compound 5

To a solution of compound **4** (3.1 g, 4.8 mmol) in DCM (50 ml) at 0°C was added 80% TFA (aq.) (15 ml), and the mixture was stirred at room temperature overnight. The reaction mixture was washed twice with water twice and then with saturated NaHCO_3_ (aq.) and brine and dried over Na_2_SO_4_. The organic phase was evaporated to dryness and purified by silica gel column chromatography (DCM/MeOH, 95:5, v/v). Compound **5** was isolated in 83% yield (2.4 g) as a white solid. ^1^H NMR (400 MHz, DMSO-*d*_6_) δH: 0.91 (3H, d, *J* = 6.3 Hz), 0.98 (9H, s), 1.10 (3H, d, *J* = 3.5 Hz), 1.12 (3H, d, *J* = 3.5 Hz) 2.76 (1H, m), 3.75 (1H, t, *J* = 3.2 Hz), 4.04 (1H, m), 4.30(1H, m), 4.42 (1H, q, *J* = 6.2 Hz), 5.16 (1H, d, *J* = 5.1 Hz), 5.58 (1H, d, *J* = 6.1 Hz), 5.74 (1H, d, *J* = 6.6 Hz), 7.31–7.47 (6H, m), 7.56–7.61(4H, m), 8.01 (1H, s), 11.69 (1H, s), 12.08 (1H, s). ^13^C NMR (126 MHz, DMSO-*d*_6_) δC: 180.10, 154.77, 149.24, 148.26, 137.11, 135.35, 135.24, 133.80, 133.23, 129.79, 129.75, 127.69, 127.65, 127.64, 120.12, 88.10, 85.26, 72.61, 69.04, 34.72, 26.78, 19.12, 18.83, 18.80, 18.79. HRMS calc. for C_31_H_40_N_5_O_6_Si [M+H]^+^: 606.2748; found: 606.2740.

#### Synthesis of compound 6

To a solution of compound **5** (2.3 g, 3.8 mmol) in pyridine (40 ml) were added benzoic anhydride (1.9 g, 8.4 mmol) and DMAP (460 mg, 3.8 mmol), and the mixture was stirred at room temperature overnight. The reaction mixture was evaporated to dryness. The resulting mixture was dissolved in AcOEt, washed with saturated NaHCO_3_ (aq.) and brine, and dried over Na_2_SO_4_. The organic phase was evaporated to dryness and purified by silica gel column chromatography (hexanes/AcOEt, 1:1, v/v). Compound **6** was isolated in 99% yield (3.1 g) as a white solid. ^1^H NMR (400 MHz, DMSO-*d*_6_) δH: 0.98 (9H, s),1.03 (1H, d, *J* = 6.2 Hz), 1.08 (3H, d, *J* = 6.8 Hz), 1.11 (3H, d, *J* = 6.8 Hz), 2.75 (1H, m), 4.30 (1H, m), 4.34 (1H, t, *J* = 4.6 Hz), 6.02 (1H, m), 6.19 (1H, t, *J* = 6.3 Hz), 6.25 (1H, d, *J* = 6.1 Hz), 7.32–7.70 (16H, m), 7.73–7.75 (2H, m), 7.89–7.92 (2H, m) 8.21 (1H, s), 11.55 (1H, s), 12.12 (1H, s). ^13^C NMR (126 MHz, DMSO-*d*_6_) δC: 180.08, 164.48, 164.22, 154.69, 148.84, 148.42, 137.54, 135.34, 135.24, 133.97, 133.93, 133.46, 133.04, 129.90, 129.88, 129.26, 129.24, 128.82, 128.66, 128.51, 128.01, 127.75, 127.73, 120.31, 85.12, 84.28, 72.43, 70.07, 68.77, 34.71, 26.69, 18.83, 18.76, 18.74. HRMS calc. for C_45_H_48_N_5_O_8_Si [M+H]^+^: 814.3272; found: 814.3282.

#### Synthesis of compound 7

To a solution of compound **6** (3.1 g, 3.8 mmol) in THF (40 ml) was added TBAF (1 M solution in THF, 11.4 ml, 11.4 mmol), and the mixture was stirred at room temperature overnight. The reaction mixture was diluted with AcOEt, washed with saturated NaHCO_3_ (aq.) and brine, and dried over Na_2_SO_4_. The organic phase was evaporated to dryness and purified by silica gel column chromatography (DCM/MeOH, 98:2, v/v). Compound **7** was isolated in 87% yield (1.9 g) as a white solid. ^1^H NMR (400 MHz, DMSO-*d*_6_) δH: 1.09 (6H, m), 1.20 (3H, d, *J* = 6.5 Hz), 2.75 (1H, m), 4.04 (1H, m), 4.29 (1H, m), 5.49 (1H, d, *J* = 5.0 Hz), 5.94 (1H, dd, *J* = 5.6 Hz, 2.2 Hz), 6.13 (1H, m), 6.31 (1H, d, *J* = 7.2 Hz), 7.35–7.39 (2H, m), 7.51–7.76 (6H, m) 7.94–7.97 (2H, m), 8.40 (1H, s), 11.59 (1H, s), 12.10 (1H, s). ^13^C NMR (126 MHz, DMSO-*d*_6_) δC: 180.13, 164.63, 164.27, 154.72, 148.89, 148.44, 137.51, 133.92, 133.90, 129.26, 129.24, 128.88, 128.79, 128.70, 128.10, 120.21, 87.11, 84.10, 73.83, 71.19, 66.10, 34.71, 19.67, 18.82, 18.79. HRMS calc. for C_29_H_30_N_5_O_8_ [M+H]^+^: 576.2094; found: 576.2095.

#### Synthesis of compound 8

To a solution of compound **7** (1.8 g, 3.1 mmol) in THF-pyridine (80 ml, 4:1, v/v) were added DMTrCl (2.1 g, 6.3 mmol) and AgNO_3_ (1.0 g, 5.9 mmol), and the mixture was stirred at room temperature overnight. The reaction mixture was filtered through Celite, diluted with AcOEt, washed with saturated NaHCO_3_ (aq.) and brine, and dried over Na_2_SO_4_. The organic phase was evaporated to dryness and purified by silica gel column chromatography (DCM/MeOH/NEt_3_, 97:2:1, v/v/v). Compound **8** was isolated in 98% yield (2.7 g) as a yellow solid. ^1^H NMR (400 MHz, DMSO-*d*_6_) δH: 1.03 (3H, d, *J* = 6.3 Hz), 1.10 (6H, m), 2.74 (1H, m), 3.62 (3H, s), 3.65 (3H, s), 3.81 (1H, m), 3.96 (1H, dd, *J* = 6.1, 2.7 Hz), 5.76 (1H, t, *J* = 6.3 Hz), 6.07 (1H, m), 6.16 (1H, d, *J* = 5.3 Hz), 6.80 (4H, m), 7.15–7.51 (13H, m), 7.60–7.70 (2H, m), 7.77 (2H, m), 7.84 (2H, m), 8.10 (1H, s), 11.51 (1H, s), 12.13 (1H, s). ^13^C NMR (101 MHz, DMSO-*d*_6_) δC: 180.09, 164.40, 164.28, 158.12, 158.08, 154.70, 148.68, 148.41, 145.93, 137.69, 136.20, 135.88, 134.03, 133.94, 130.09, 129.98, 129.24, 128.81, 128.76, 128.52, 128.08, 127.77, 127.70, 126.65, 120.28, 113.10, 113.05, 86.18, 84.64, 84.15, 72.88, 70.26, 69.24, 54.91, 54.88, 34.74, 18.83, 18.81, 16.16. HRMS calc. for C_50_H_48_N_5_O_10_ [M+H]^+^: 878.3401; found: 878.3427.

#### Synthesis of compound 9

To a solution of compound **8** (2.7 g, 3.1 mmol) in THF-MeOH (70 ml, THF/MeOH, 1:1, v/v) at 0°C was added NaOH (1 M solution in water, 12.3 ml, 12.3 mmol), and the mixture was stirred at 0°C for 30 min. The reaction mixture was diluted with DCM, washed with brine, and dried over Na_2_SO_4_. The organic phase was evaporated to dryness and purified by silica gel column chromatography (DCM/MeOH/NEt_3_, 97:2:1, v/v/v). Compound **9** was isolated in 96% yield (2.0 g) as a white solid. ^1^H NMR (400 MHz, DMSO-*d*_6_) δ: 0.65 (3H, d, *J* = 6.3 Hz), 1.10 (3H, d, *J* = 3.4 Hz), 1.12 (3H, d, *J* = 3.4 Hz), 2.75 (1H, m), 3.53 (1H, m), 3.71 (3H, s), 3.72 (3H, s), 3.75 (1H, t, *J* = 3.8 Hz), 4.23 (1H, m), 4.36 (1H, m), 5.07 (1H, d, *J* = 5.2 Hz), 5.53 (1H, d, *J* = 6.0 Hz), 5.71 (1H, d, *J* = 6.1 Hz), 6.80–6.85 (4H, m), 7.19–7.28 (7H, m), 7.39–7.41 (2H, m), 7.89 (1H, s), 11.67 (1H, s), 12.08 (1H, s). ^13^C NMR (126 MHz, DMSO-*d*_6_) δC: 180.11, 158.06, 158.03, 154.76, 149.14, 148.25, 146.22, 137.04, 136.47, 136.38, 130.11, 130.07, 127.90, 127.59, 126.57, 120.07, 112.97, 87.58, 85.69, 85.68, 72.81, 69.48, 69.33, 54.99, 54.98, 54.97, 34.74, 18.83, 18.81, 16.82. HRMS calc. for C_36_H_40_N_5_O_8_ [M+H]^+^: 670.2877; found: 670.2872.

#### Synthesis of compound 10

To a solution of compound **9** (1.9 g, 2.8 mmol) in THF-pyridine (30 ml, 4:1, v/v) were added TBSCl (1.7 g, 11.3 mmol) and AgNO_3_ (1.0 g, 5.9 mmol), and the mixture was stirred at room temperature overnight. The reaction mixture was filtered through Celite, diluted with DCM, washed with saturated NaHCO_3_ (aq.) and brine, and dried over Na_2_SO_4_. The organic phase was evaporated to dryness and purified by silica gel column chromatography (DCM/AcOEt/NEt_3_, 80:19:1, v/v/v). Compound **10** was isolated in 50% yield (1.1 g) as a white solid. ^1^H NMR (400 MHz, DMSO-*d*_6_) δ: –0.16 (3H, s), -0.03 (3H, s), 0.72 (9H, s), 0.75 (3H, d, *J* = 6.3 Hz), 1.09 (3H, d, *J* = 2.8 Hz), 1.11 (3H, d, *J* = 2.8 Hz), 2.74 (1H, m), 3.54 (1H, m), 3.718 (3H, s), 3.721 (3H, s), 3.75 (1H, t, *J* = 3.6 Hz), 4.24 (1H, m), 4.33 (1H, t, *J* = 5.9 Hz), 4.92 (1H, d, *J* = 5.8 Hz), 5.76 (1H, d, *J* = 6.2 Hz), 6.83–6.87 (4H, m), 7.18–7.30 (7H, m), 7.40–7.42 (2H, m), 7.79 (1H, s), 11.67 (1H, s), 12.07 (1H, s). ^13^C NMR (126 MHz, DMSO-*d*_6_) δC: 180.11, 158.08, 154.70, 149.07, 148.33, 146.25, 136.60, 136.30, 136.22, 130.09, 130.06, 127.81, 127.61, 126.63, 119.86, 112.99, 87.96, 85.75, 85.45, 75.59, 69.52, 69.22, 54.99, 34.72, 25.44, 18.82, 18.78, 17.74, 16.99, –4.89, –5.44. HRMS calc. for C_42_H_54_N_5_O_8_Si [M+H]^+^: 784.3742; found: 784.3745.

#### Synthesis of compound 11

To a solution of compound **10** (0.9 g, 1.2 mmol) in DCM (10 ml) were added DIPEA (1.2 ml, 6.9 mmol), 1-methylimidazole (180 μl, 2.3 mmol), and *i*-Pr_2_NP(Cl)O(CH_2_)_2_CN (1.0 ml, 4.6 mmol), and the mixture was stirred at room temperature for 2 h. The reaction mixture was diluted with DCM, washed with saturated NaHCO_3_ (aq.) and brine, and dried over Na_2_SO_4_. The organic phase was evaporated to dryness and purified by precipitation with hexanes and then further purified by silica gel column chromatography (hexanes/acetone/NEt_3_, 50:49:1, v/v/v). Compound **11** was isolated in 88% yield (1.0 g) as a white solid. ^1^H NMR (500 MHz, CDCl_3_) δ: -0.27 (2H, s), –0.23 (2.8H, s), –0.07 (2H, s), 0.01 (2.8H, s), 0.72 (3H, d, *J* = 6.4 Hz), 0.79 (15H, m), 0.90 (2H, d, *J* = 6.4 Hz), 1.05 (4H, d, *J* = 6.9 Hz), 1.12–1.21 (30H, m), 2.17 (0.6H, s), 2.26 (1H, m), 2.45 (0.7H, m), 2.52–2.65 (3H, m), 2.76 (0.7H, m), 2.85 (0.7H, m), 3.41–3.47 (0.8H, m), 3.53–3.70 (5H, m), 3.78 (10H, m), 3.85 (2.4H, m), 4.02 (1.6H, m), 4.24–4.38 (2.5H, m), 4.51–4.63 (2.8H, m), 5.75 (1H, d, *J* = 6.2 Hz), 5.89 (0.7H, d, *J* = 7.8 Hz), 6.80 (6.7H, m), 7.19–7.55 (18H, m), 8.33 (1H, s), 9.06 (0.7H, s), 12.00 (1.4H, s). ^13^C NMR (126 MHz, CDCl_3_) δ 179.28, 178.83, 158.85, 158.80, 155.79, 155.69, 149.12, 148.58, 148.10, 147.59, 146.35, 146.30, 138.05, 137.12, 136.90, 136.51, 136.47, 136.25, 130.75, 130.69, 130.48, 130.44, 128.64, 128.20, 128.02, 127.91, 127.16, 127.12, 122.15, 121.15, 118.38, 117.98, 113.39, 113.32, 113.21, 113.18, 88.99, 88.96, 88.52, 87.64, 87.61, 87.01, 86.75, 85.57, 74.66, 74.63, 72.69, 72.63, 71.86, 71.74, 70.63, 69.90, 59.17, 59.10, 57.49, 57.36, 55.46, 55.44, 46.39, 43.53, 43.43, 43.22, 43.12, 36.19, 36.06, 25.82, 25.78, 25.02, 24.96, 24.93, 24.91, 24.88, 20.50, 20.45, 20.30, 19.22, 19.05, 19.00, 18.23, 18.07, 17.30, 0.19, –4.28, –4.67, –4.71, –4.88, –5.07. ^31^P NMR (202 MHz, CDCl_3_) δ: 148.95, 149.02. HRMS; calc. for C_51_H_71_N_7_O_9_PSi [M+H]^+^: 984.4620; found: 984.4625

#### Synthesis of compound 15

To a solution of compound **14** (3.5 g, 5.4 mmol) in DCM (55 ml) at 0°C was added 80% TFA (aq.) (17 ml), and the mixture was stirred at room temperature overnight. The reaction mixture was washed with water twice then with saturated NaHCO_3_ (aq.) and brine and dried over Na_2_SO_4_. The organic phase was evaporated to dryness, and the resulting solid was washed with DCM. Compound **15** was isolated in 70% yield (2.3 g) as a white solid. ^1^H NMR (400 MHz, DMSO-*d*_6_) δ: 0.96 (9H, s), 0.98 (3H, d, *J* = 6.4 Hz), 1.10 (3H, d, *J* = 2.6 Hz), 1.12 (3H, d, *J* = 2.6 Hz), 2.76 (1H, m), 3.78 (1H, t, *J* = 4.6 Hz), 3.99 (1H, m), 4.22 (1H, q, *J* = 5.1 Hz), 4.41 (1H, q, *J* = 5.5 Hz), 5.14 (1H, d, *J* = 5.5 Hz), 5.61 (1H, dd, *J* = 5.8 Hz), 5.80 (1H, d, *J* = 5.3 Hz), 7.31–7.43 (6H, m), 7.58–7.60 (4H, m), 8.10 (1H, s), 11.67 (1H, s), 12.09 (1H, s). ^13^C NMR (126 MHz, DMSO-*d*_6_) δC: 180.10, 154.78, 148.79, 148.14, 136.96, 135.30, 135.26, 133.80, 132.80, 129.77, 129.68, 127.63, 127.58, 120.23, 87.75, 86.30, 73.74, 69.88, 69.62, 34.69, 26.77, 19.39, 18.88, 18.84, 18.79. HRMS calc. for C_31_H_40_N_5_O_6_Si [M+H]^+^: 606.2748; found: 606.2742.

#### Synthesis of compound 16

To a solution of compound **15** (2.3 g, 3.8 mmol) in pyridine (40 ml) were added benzoic anhydride (1.9 g, 8.4 mmol) and DMAP (460 mg, 3.8 mmol), and the mixture was stirred at room temperature overnight. The reaction mixture was evaporated to dryness. The resulting mixture was dissolved in AcOEt, washed with saturated NaHCO_3_ (aq.) and brine, and dried over Na_2_SO_4_. The organic phase was evaporated to dryness and purified by silica gel column chromatography (hexanes/AcOEt, 1:1, v/v). Compound **16** was isolated in 99% yield (3.1 g) as a white solid. ^1^H NMR (400 MHz, DMSO-*d*_6_) δ: 0.97 (9H, s), 1.10 (9H, m), 2.74 (1H, m), 4.25 (1H, m), 4.43 (1H, t, *J* = 4.8 Hz), 5.96 (1H, m), 6.12 (1H, t, *J* = 5.9 Hz), 6.32 (1H, d, *J* = 5.5 Hz), 7.25–7.29 (2H, m), 7.36–7.52 (8H, m), 7.57–7.69 (6H, m), 7.78–7.80 (2H, m), 7.91–7.94 (2H, m), 8.24 (1H, s), 11.47 (1H, s), 12.12 (1H, s). ^13^C NMR (126 MHz, DMSO-*d*_6_) δC: 180.03, 164.58, 164.24, 154.72, 148.57, 148.30, 137.31, 135.30, 135.24, 133.97, 133.89, 133.45, 132.57, 129.82, 129.79, 129.30, 129.25, 128.78, 128.69, 128.55, 128.05, 127.62, 127.60, 120.53, 85.31, 84.92, 73.44, 70.60, 69.13, 34.70, 26.68, 18.87, 18.81, 18.80, 18.72. HRMS calc. for C_45_H_48_N_5_O_8_Si [M+H]^+^: 814.3272; found: 814.3272.

#### Synthesis of compound 17

To a solution of compound **16** (3.1 g, 3.8 mmol) in THF (40 ml) was added TBAF (1 M solution in THF, 11.1 ml, 11.1 mmol), and the mixture was stirred at room temperature overnight. The reaction mixture was diluted with DCM, washed with brine, and dried over Na_2_SO_4_. The organic phase was evaporated to dryness and purified by silica gel column chromatography (DCM/MeOH, 98:2, v/v). Compound **17** was isolated in 94% yield (2.0 g) as a white solid. ^1^H NMR (400 MHz, DMSO-*d*_6_) δ: 1.07 (3H, d, *J* = 6.8 Hz), 1.09 (3H, d, *J* = 6.8 Hz), 1.20 (3H, d, *J* = 6.4 Hz), 2.73 (1H, m), 4.07 (1H, m), 4.39 (1H, t, *J* = 2.5 Hz), 5.54 (1H, d, *J* = 4.9 Hz), 5.83 (1H, dd, *J* = 5.4, 2.5 Hz), 6.05 (1H, m), 6.32 (1H, d, *J* = 6.7 Hz), 7.38–7.42 (2H, m), 7.50–7.70 (4H, m), 7.76–7.78 (2H, m), 7.93–7.95 (2H, m), 8.45 (1H, s), 11.57 (1H, s), 12.10 (1H, s). ^13^C NMR (126 MHz, DMSO-*d*_6_) δC: 180.11, 164.76, 164.32, 154.71, 148.83, 148.47, 137.11, 133.92, 133.88, 129.26, 129.22, 128.87, 128.80, 128.72, 128.17, 120.01, 86.80, 84.28, 74.60, 73.12, 65.73, 34.69, 19.30, 18.80, 18.78. HRMS calc. for C_29_H_30_N_5_O_8_ [M+H]^+^: 576.2094; found: 576.2103.

#### Synthesis of compound 18

To a solution of compound **17** (1.9 g, 3.3 mmol) in THF-pyridine (80 ml, 4:1, v/v) were added DMTrCl (2.2 g, 6.6 mmol) and AgNO_3_ (1.1 g, 6.6 mmol), and the mixture was stirred at room temperature overnight. The reaction mixture was filtered through Celite, diluted with AcOEt, washed with saturated NaHCO_3_(aq.) and brine, and dried over Na_2_SO_4_. The organic phase was evaporated to dryness and purified by silica gel column chromatography (DCM/MeOH/NEt_3_, 97:2:1, v/v/v). Compound **18** was isolated in 97% yield (2.8 g) as a yellow solid. ^1^H NMR (400 MHz, DMSO-*d*_6_) δ: 0.99 (3H, d, *J* = 6.2 Hz), 1.10 (6H, m), 2.75 (1H, m), 3.66 (3H, s), 3.68 (3H, s), 3.87 (1H, m), 3.94 (1H, t, *J* = 5.1 Hz), 6.05 (1H, m), 6.16 (2H, m), 6.72–7.91 (5H, m), 7.16–7.40 (10H, m), 7.46–7.50 (2H, m), 7.58–7.67 (2H, m), 7.74–7.76 (2H, m), 7.88–7.90 (2H, m), 8.30 (1H, s), 11.42 (1H, s), 12.11 (1H, s). ^13^C NMR (126 MHz, DMSO-*d*_6_) δC: 180.21, 164.53, 164.34, 158.26, 158.20, 154.84, 148.71, 148.37, 145.74, 138.21, 136.27, 136.08, 134.14, 133.96, 130.18, 130.03, 129.40, 129.38, 128.89, 128.85, 128.78, 128.21, 127.96, 127.73, 126.80, 120.68, 113.13, 113.08, 86.24, 85.08, 82.59, 72.99, 69.40, 68.34, 55.10, 55.08, 34.86, 19.03, 18.91, 16.34. HRMS calc. for C_50_H_48_N_5_O_10_ [M+H]^+^: 878.3401; found: 878.3398.

#### Synthesis of compound 19

To a solution of compound **18** (2.7 g, 3.1 mmol) in THF–MeOH (70 ml, 1:1, v/v) at 0°C was added NaOH (1 M solution in water, 12.3 ml, 12.3 mmol), and the mixture was stirred at 0°C for 30 min. The reaction mixture was diluted with DCM, washed with brine, and dried over Na_2_SO_4_. The organic phase was evaporated to dryness and purified by silica gel column chromatography (DCM/MeOH/NEt_3_, 97:2:1, v/v/v). Compound **19** was isolated in 96% yield (2.0 g) as a white solid. ^1^H NMR (400 MHz, DMSO-*d*_6_) δ: 0.65 (3H, d, *J* = 6.2 Hz), 1.11 (6H, d, *J* = 6.8 Hz), 2.75 (1H, m), 3.57 (1H, m), 3.71 (7H, m), 4.19 (1H, m), 4.45 (1H, m), 5.12 (1H, d, *J* = 4.9 Hz), 5.51 (1H, d, *J* = 6.3 Hz), 5.69 (1H, d, *J* = 6.7 Hz), 6.81 (4H, m), 7.18–7.29 (7H, m), 7.40 (2H, m), 8.13 (1H, s), 11.61 (1H, s), 12.06 (1H, s). ^13^C NMR (126 MHz, DMSO-*d*_6_) δC: 180.06, 158.05, 157.99, 154.76, 149.11, 148.13, 146.08, 137.36, 136.46, 136.33, 130.16, 130.05, 127.94, 127.53, 126.55, 120.28, 112.91, 112.88, 86.91, 85.66, 85.37, 72.72, 69.20, 69.06, 54.97, 54.95, 34.72, 18.86, 18.77, 16.82. HRMS calc. for C_36_H_40_N_5_O_8_ [M+H]^+^: 670.2877; found: 670.2887.

#### Synthesis of compound 20

To a solution of compound **19** (2.3 g, 3.4 mmol) in THF-pyridine (30 ml, 4:1, v/v) were added TBSCl (1.8 g, 12.0 mmol) and AgNO_3_ (1.2 g, 6.9 mmol), and the mixture was stirred at room temperature overnight. The reaction mixture was filtered through Celite, diluted with DCM, washed with saturated NaHCO_3_ (aq.) and brine, and dried over Na_2_SO_4_. The organic phase was evaporated to dryness and purified by silica gel column chromatography (DCM/AcOEt/NEt_3_, 80:19:1, v/v/v). Compound **20** was isolated in 53% yield (1.4 g) as a white solid. ^1^H NMR (400 MHz, DMSO-*d*_6_) δ: –0.20 (3H, s), -0.04 (3H, s), 0.71 (12H, m), 1.10 (6H, d, *J* = 6.8 Hz), 2.74 (1H, m), 3.56 (1H, m), 3.72 (7H, m), 4.09 (1H, m), 4.45 (1H, m), 4.92 (1H, d, *J* = 5.76 Hz), 5.76 (1H, d, *J* = 6.9 Hz), 6.83 (4H, m), 7.17–7.32 (7H, m), 7.42 (2H, m), 8.18 (1H, s), 11.58 (1H, s), 12.07 (1H, s). ^13^C NMR (126 MHz, DMSO-*d*_6_) δC: 180.06, 158.06, 158.02, 154.75, 149.05, 148.19, 146.15, 137.11, 136.38, 136.29, 130.10, 130.03, 127.84, 127.58, 126.55, 120.12, 112.96, 112.94, 87.79, 85.83, 85.21, 75.42, 69.33, 69.14, 54.97, 54.96, 34.71, 25.45, 18.86, 18.71, 17.67, 17.08, –4.90, –5.51. HRMS calc. for C_42_H_54_N_5_O_8_Si [M+H]^+^: 784.3742; found: 784.3751.

#### Synthesis of compound 21

To a solution of compound **20** (1.0 g, 1.3 mmol) in DCM (10 ml) were added DIPEA (1.3 ml, 7.7 mmol), 1-methylimidazole (0.2 ml, 2.6 mmol), and *i*-Pr_2_NP(Cl)O(CH_2_)_2_CN (0.7 ml, 3.2 mmol), and the mixture was stirred at room temperature for 1 h. The reaction mixture was diluted with DCM, washed with saturated NaHCO_3_ (aq.) and brine, and dried over Na_2_SO_4_. The organic phase was evaporated to dryness, precipitated with hexanes and purified by silica gel column chromatography (hexanes/acetone/NEt_3_, 50:49:1, v/v/v). Compound **21** was isolated in 65% yield (1.0 g) as a white solid. ^1^H NMR (500 MHz, CDCl_3_) δ: –0.29 (3H, s), –0.25 (2H, s), –0.16 (3H, s), –0.03 (2H, s), 0.53 (2H, d, *J* = 6.8 Hz), 0.70 (2H, d, *J* = 6.9 Hz), 0.76 (15H, m), 0.94–1.02 (18H, m), 1.12–1.20 (15H, m), 2.04 (2H, m), 2.17 (0.5H, s), 2.25 (0.8H, m), 2.71 (1.2H, m), 2.82 (1H, m), 3.39–3.61 (7H, m), 3.78 (10H, m), 3.96 (2H, m), 4.06 (1H, m), 4.14 (1.8H, m), 4.25 (0.7H, m), 4.86 (1H, m), 5.35 (0.7H, m), 5.64 (0.7H, d, *J* = 8.1 Hz), 5.90 (1H, d, *J* = 8.2 Hz), 6.82 (7H, m), 7.19–7.29 (7H, m), 7.41–7.59 (7H, m), 7.70 (1.3H, m), 7.87 (0.7H, s), 8.10 (1H, s), 8.61 (1H, s), 12.02 (1.7H, brs). ^13^C NMR (101 MHz, CDCl_3_) δ:178.88, 178.18, 158.69, 158.65, 158.59, 155.61, 155.55, 148.74, 148.27, 147.62, 146.85, 146.24, 139.66, 137.27, 136.57, 136.38, 130.20, 130.16, 130.14, 130.04, 128.01, 127.95, 127.89, 126.99, 126.93, 123.09, 121.86, 118.17, 117.17, 113.28, 113.22, 113.20, 113.16, 89.74, 89.70, 89.67, 88.61, 86.78, 86.28, 85.87, 73.83, 73.71, 73.01, 72.87, 72.82, 72.75, 70.53, 70.34, 60.39, 59.03, 58.92, 57.24, 57.06, 55.28, 55.24, 43.54, 43.42, 42.82, 42.70, 35.96, 35.85, 25.63, 25.60, 24.80, 24.71, 24.63, 21.05, 20.15, 20.12, 19.75, 19.68, 18.63, 18.58, 18.54, 18.35, 18.01, 17.93, 17.91, 17.77, 14.20, –4.46, –4.85, –4.89, –5.28. ^31^P NMR (202 MHz, CDCl_3_) δ: 148.82, 151.74. HRMS; calc. for C_51_H_71_N_7_O_9_PSi [M+H]^+^: 984.4820; found: 984.4805

#### Synthesis of compound 3 for Mosher ester analysis

To a solution of compound **4** (300 mg, 0.47 mmol) in THF (5 ml) was added TBAF (1 M solution in THF, 1.4 ml, 1.4 mmol), and the mixture was stirred at room temperature overnight. The reaction mixture was diluted with DCM, washed with brine, and dried over Na_2_SO_4_. The organic phase was evaporated to dryness and purified by silica gel column chromatography (AcOEt/MeOH, 95:5, v/v). Compound **3** was isolated in 82% yield (155 mg) as a white solid. ^1^H NMR (400 MHz, DMSO-*d*_6_) δH: 1.01 (3H, d, *J* = 6.4 Hz), 1.12 (6H, d, *J* = 6.8 Hz), 1.32 (3H, s), 1.51 (3H, s), 2.77 (1H, m), 3.65 (1H, m), 3.86 (1H, dd, *J* = 5.6, 2.9 Hz), 5.06 (2H, m), 5.24 (1H, dd, *J* = 6.4, 2.9 Hz), 5.98 (1H, d, *J* = 2.9 Hz), 8.23 (1H, s), 11.59 (1H, s), 12.11 (1H, s). ^13^C NMR (126 MHz, DMSO-*d*_6_) δC: 180.17, 154.77, 148.38, 148.20, 137.85, 120.08, 113.15, 89.95, 88.26, 83.40, 80.28, 65.92, 34.72, 27.00, 25.18, 19.83, 18.85. HRMS calc. for C_18_H_26_N_5_O_6_ [M+H]^+^: 408.1883; found: 408.1889.

#### Synthesis of compound 23 for Mosher ester analysis

To a solution of compound **3** (25 mg, 0.064 mmol) in DCM-pyridine (1.2 ml, 5:1, v/v) at 0°C was added (*R*)-(−)-α-methoxy-α-(trifluoromethyl)phenylacetyl chloride ((*R*)-(–)-MTPACl) (1.0 M in DCM, 0.064 ml, 0.064 mmol), and the mixture was stirred at room temperature overnight. The reaction mixture was washed with saturated NaHCO_3_ (aq.) and brine and dried over Na_2_SO_4_. The organic phase was evaporated to dryness and purified by silica gel column chromatography (DCM/MeOH, 97.5:2.5, v/v). Compound **23** was isolated in 72% yield (28 mg) as a white solid. ^1^H NMR (400 MHz, DMSO-*d*_6_) δH: 1.12 (6H, m), 1.23 (3H, d, *J* = 6.4 Hz), 1.28 (3H, s), 1.48 (3H, s), 2.75 (1H, m), 3.45 (3H, s), 4.03 (1H, dd, *J* = 6.4, 4.1 Hz), 4.98 (1H, dd, *J* = 6.5, 4.1 Hz), 5.24 (1H, m), 5.35 (1H, dd, *J* = 6.5, 2.8 Hz), 6.02 (1H, d, *J* = 2.9 Hz), 7.43–7.46 (5H, m), 8.13 (1H, s), 11.45 (1H, s), 12.07 (1H, s). HRMS calc. for C_28_H_32_F_3_N_5_O_8_ [M+H]^+^: 624.2281; found: 624.2301.

#### Synthesis of compound 24 for Mosher ester analysis

To a solution of compound **3** (25 mg, 0.064 mmol) in DCM-pyridine (1.2 ml, 5:1, v/v) at 0°C was added (*S*)-(+)-α-methoxy-α-(trifluoromethyl)phenylacetyl chloride ((*S*)-(+)-MTPACl) (1.0 M in DCM, 0.064 ml, 0.064 mmol), and the mixture was stirred at room temperature overnight. The reaction mixture was washed with saturated NaHCO_3_ (aq.) and brine and dried over Na_2_SO_4_. The organic phase was evaporated to dryness and purified by silica gel column chromatography (DCM/MeOH, 97.5:2.5, v/v). Compound **24** was isolated in 38% yield (15 mg) as a white solid. ^1^H NMR (400 MHz, DMSO-*d*_6_) δH: 1.12 (9H, m), 1.34 (3H, s), 1.52 (3H, s), 2.74 (1H, m), 3.50 (3H, s), 4.09 (1H, dd, *J* = 6.4, 4.3 Hz), 5.12 (1H, dd, *J* = 6.6, 4.3 Hz), 5.24 (1H, m), 5.37 (1H, dd, *J* = 6.6, 2.7 Hz), 6.07 (1H, d, *J* = 2.7 Hz), 7.41–7.46 (5H, m), 8.17 (1H, s), 11.44 (1H, s), 12.08 (1H, s). HRMS calc. for C_28_H_32_F_3_N_5_O_8_ [M+H]^+^: 624.2281; found: 624.2272.

#### Synthesis of compound 25

To a solution of compound **13** (25 mg, 0.064 mmol) in DCM-pyridine (1.2 ml, DCM/pyridine, 5:1, v/v) at 0°C were added (*R*)-(–)-MTPACl (1.0 M in DCM, 0.064 ml, 0.064 mmol), and the mixture was stirred at room temperature overnight. The reaction mixture was washed with saturated NaHCO_3_ (aq.) and brine and dried over Na_2_SO_4_. The organic phase was evaporated to dryness and purified by silica gel column chromatography (DCM/MeOH, 97.5:2.5, v/v). Compound **25** was isolated in 31% yield (12 mg) as a white solid. ^1^H NMR (500 MHz, DMSO-*d*_6_) δH: 1.14 (6H, m), 1.26 (3H, d, *J* = 6.3 Hz), 1.35 (3H, s), 1.52 (3H, s), 2.78 (1H, m), 4.10 (1H, dd, *J* = 9.3, 2.9 Hz), 5.24 (1H, m), 5.33 (1H, m), 6.19 (1H, d, *J* = 1.2 Hz), 7.30–7.42 (5H, m), 8.14 (1H, s), 11.48 (1H, s), 12.07 (1H, s). HRMS calc. for C_28_H_32_F_3_N_5_O_8_ [M+H]^+^: 624.2281; found: 624.2299.

#### Synthesis of compound 26

To a solution of compound **13** (25 mg, 0.064 mmol) in DCM-pyridine (1.2 ml, DCM/pyridine, 5:1, v/v) at 0°C was added (*S*)-(+)-MTPACl (1.0 M in DCM, 0.064 ml, 0.064 mmol), and the mixture was stirred at room temperature overnight. The reaction mixture was washed with saturated NaHCO_3_ (aq.) and brine and dried over Na_2_SO_4_. The organic phase was evaporated to dryness and purified by silica gel column chromatography (DCM/MeOH, 97.5:2.5, v/v). Compound **26** was isolated in 51% yield (20 mg) as a white solid. ^1^H NMR (500 MHz, DMSO-*d*_6_) δH: 1.13 (6H, m), 1.28 (3H, s), 1.35 (3H, d, *J* = 6.4 Hz), 1.48 (3H, s), 2.77 (1H, m), 3.38 (3H, s), 4.13 (1H, dd, *J* = 7.6, 2.9 Hz), 4.80 (1H, dd, *J* = 6.1, 2.3 Hz),5.08 (1H, dd, *J* = 6.2, 3.0 Hz), 5.29 (1H, m), 6.03 (1H, d, *J* = 2.4 Hz), 7.28–7.42 (5H, m), 8.08 (1H, s), 11.47 (1H, s), 12.08 (1H, s). HRMS calc. for C_28_H_32_F_3_N_5_O_8_ [M+H]^+^: 624.2281; found: 624.2272.

#### Synthesis of controlled pore glass (CPG) support 12

To a solution of compound **10** (100 mg, 0.13 mmol) in DCM (1 ml) were added succinic anhydride (26 mg, 0.26 mmol) and DMAP (48 mg, 0.39 mmol), and the mixture was stirred at room temperature overnight. The reaction mixture was evaporated to dryness and purified by silica gel column chromatography (DCM/MeOH/TEA, 95:4:1, v/v/v). To a solution of the obtained succinate in DMF (4 ml) were added to CPG functionalized with long chain amino alkyl (LCAA) (pore size 500 Å NH_2_, loading of 171 μmol/g, 760 mg), DIPEA (0.09 ml, 0.5 mmol), and HBTU (53 mg, 0.14 mmol), and the mixture was agitated on a wrist-action shaker at room temperature overnight. The obtained CPG was filtered, washed with DCM-MeOH (9:1, v/v), and dried. To the suspension of the obtained CPG in pyridine (3 ml) was added acetic anhydride (1 ml), and the reaction mixture was agitated on a wrist-action shaker at room temperature overnight. The CPG was filtered, washed with DCM/MeOH (9:1, v/v), and dried to give CPG support **12** (760 mg, 70.2 μmol/g).

#### Synthesis of CPG support 22

To a solution of compound **20** (100 mg, 0.13 mmol) in DCM (1 ml) were added succinic anhydride (26 mg, 0.26 mmol) and DMAP (48 mg, 0.39 mmol), and the mixture was stirred at room temperature overnight. The reaction mixture was evaporated to dryness and purified by silica gel column chromatography (DCM/MeOH/TEA, 95:4:1, v/v/v). To a solution of the obtained succinate in DMF (4 ml) were added LCAA-CPG (pore size 500 Å NH_2_, loading of 171 μmol/g, 820 mg), DIPEA (0.09 ml, 0.5 mmol), and HBTU (58 mg, 0.15 mmol), and the mixture was agitated on a wrist-action shaker at room temperature overnight. The obtained CPG was filtered, washed with DCM/MeOH (9:1, v/v) and dried. To the suspension of the obtained CPG in pyridine (3 ml) was added acetic anhydride (1 ml), and reaction mixture was agitated on a wrist-action shaker at room temperature overnight. The CPG was filtered, washed with DCM/MeOH (9:1, v/v), and dried to give CPG support **22** (850 mg, 97.0 μmol/g).

#### Triphosphate synthesis

Triphosphates were synthesized following previously described protocols (17).

#### Characterization of compound 30

^1^H NMR (500 MHz, D_2_O) δH: 1.40 (3H, d, *J* = 6.4 Hz), 4.09 (1H, d, *J* = 4.5 Hz), 4.63 (2H, m), 4.90 (1H, dd, *J* = 7.0, 5.4 Hz), 5.92 (1H, d, *J* = 7.0 Hz), 8.19 (1H, s). ^31^P NMR (202 MHz, D_2_O) δ: –22.45 (t, *J* = 19.2 Hz), –11.31 (d, *J* = 19.1 Hz), –10.12 (d, *J* = 19.3 Hz). HRMS calc. for C_11_H_18_N_5_O_14_P_3_ [M+Na]^+^: 559.9961; found: 559.9981.

#### Characterization of compound 34

^1^H NMR (500 MHz, D_2_O) δH: 1.41 (3H, d, *J* = 6.5 Hz), 4.18 (1H, m), 4.60 (1H, dd, *J* = 5.0, 3.3 Hz), 4.66 (1H, m), 5.98 (1H, d, *J* = 6.1 Hz), 8.32 (1H, s). ^31^P NMR (202 MHz, D_2_O) δ: –22.37 (t, *J* = 19.1 Hz), –11.25 (d, *J* = 18.7 Hz), –10.06 (d, *J* = 19.4 Hz). HRMS calc. for C_11_H_18_N_5_O_14_P_3_ [M+Na]^+^: 559.9961; found: 559.9963.

### Oligonucleotide synthesis

Oligonucleotides used for the exonuclease assay were synthesized on an ABI-394 and those used for *in vitro* efficacy assays were synthesized on a MerMade 192 synthesizer on 1-μmol scale using universal or custom supports. A solution of 0.25 M 5-(*S*-ethylthio)-1*H-*tetrazole in acetonitrile (CH_3_CN) was used as the activator. The solutions of commercially available phosphoramidites and synthesized 5′-(*R*)-*C*-methyl-guanosine phosphoramidities were 0.15 M in anhydrous CH_3_CN or ACN/DMF (9:1, v/v). The 5′-(*S*)-*C*-methyl-guanosine phosphoramidities were 0.15 M in anhydrous 15% DCM in CH_3_CN. The oxidizing reagent was 0.02 M I_2_ in THF/pyridine/H_2_O. The detritylation reagent was 3% dichloroacetic acid in CH_2_Cl_2_. After completion of the automated synthesis, the oligonucleotide was manually released from support and deprotected using aqueous MeNH_2_ (40% wt) at room temperature for 90 minutes. After filtration through a 0.45-μm nylon filter, oligonucleotides were either purified or, for oligonucleotides containing ribose sugars, the 2′ hydroxyl was deprotected by treatment with Et_3_N·3HF at 60°C for 30 min. Oligonucleotides were purified using IEX-HPLC using an appropriate gradient of mobile phase (buffer A: 0.15 M NaCl, 10% CH_3_CN; buffer B 1.0 M NaBr, 10% MeCN) and desalted using size-exclusion chromatography with water as an eluent. Oligonucleotides were then quantified by measuring the absorbance at 260 nm. Extinction coefficients were calculated using the following extinction coefficients for each residue: A, 13.86; T/U, 7.92; C, 6.57 and G, 10.53 M^−1^cm^−1^. The purity and identity of modified ONs were verified by analytical anion exchange chromatography and mass spectrometry, respectively.

After the trityl-off synthesis using the MerMade 192, columns were incubated with 150 μl of 40% aqueous methylamine for 30 min at room temperature, and solutions were drained via vacuum into a 96-well plate. After repeating the incubation and draining with a fresh portion of aqueous methylamine, the plate containing the crude oligonucleotides was sealed and shaken at room temperature for 60 min to completely remove all protecting groups. In the case of RNA, the 2′ hydroxyl was deprotected by treating with Et_3_N·3HF at 60°C for 60 min. Precipitation of the crude oligonucleotides was accomplished via the addition of 1.2 ml of ACN/EtOH (9:1, v/v) to each well, followed by centrifugation at 3000 rpm for 45 min at 4°C. The supernatant was removed from each well, and the pellets were resuspended in 950 μl of 20 mM aqueous NaOAc. Oligonucleotides were desalted over a GE Hi-Trap desalting column (Sephadex G25 Superfine) using water as an eluant. The identities and purities of all oligonucleotides were confirmed using ESI-MS and IEX-HPLC, respectively.

For oligonucleotides synthesized using the ABI 394, the manufacturer's standard protocols were used for cleavage and deprotection. Crude oligonucleotides were purified using strong anion exchange with phosphate buffers (pH 8.5) containing NaBr. The identities and purities of all oligonucleotides were confirmed using ESI-LC/MS and IEX-HPLC, respectively (Supplementary Tables S1 and S2).

### Evaluation of use of modified nucleotides as polymerase substrates

Purified exonuclease activity-deficient human mitochondrial DNA polymerase POLG was obtained from the lab of Prof. William Copeland (National Institute of Environmental Health Science, Durham, NC, USA). Human mitochondrial POLRMT was purchased from Indigo Biosciences (Cat# MV100–40). Atto-425-labeled DNA and RNA primers were synthesized in house; DNA templates were obtained from IDT (primer and template sequences are listed in Supplementary Table S3).

Reaction conditions for the POLG incorporation assays were as follows: 100 nM DNA template, 100 nM 5′-Atto-425-labeled DNA primer, 40 units POLG, 100 μM or 1 mM NTP substrate in 20 mM Tris–HCl, pH 8.0, 2 mM β-mercaptoethanol, 0.1 mg/ml bovine serum albumin, 10 mM MgCl_2_. Reactions were incubated at 37°C for 30 min. Reactions were quenched by heating the reaction mixture to 85°C for 5 min. The reaction mixtures were diluted with water to a final primer concentration of approximately 2.5 nM and analysed by IEX-HPLC with an attached fluorescence detector (excitation wavelength: 436 nm, emission wavelength: 485 nm) and a Dionex BioLCDNAPac PA200 4 × 250 mm (8 μm particle size) column. Buffer A was 20 mM sodium phosphate, 15% ACN, pH 11; and buffer B was 20 mM sodium phosphate, 15% ACN, 1 M NaBr, pH 11. The flow rate was 1 ml/min, and the gradient was 25% to 40% buffer B in 16 min. Reaction conditions for the POLRMT incorporation assays were as follows: 200 nM DNA template, 50 nM 5′-Atto-425-labeled RNA primer, 300 nM POLRMT, 100 μM or 1 mM NTP substrate in 20 mM Tris–HCl, pH 8, 10 mM MgCl_2_, 10 mM DTT, 0.05% Tween-20. Reactions were incubated at 37°C for 30 min and were quenched by heating the reaction mixture to 85°C for 5 min. The reaction mixtures were diluted with water to a final primer concentration of ∼2.5 nM and analysed by IEX-HPLC as described above, except that the gradient was 35% to 40% buffer B in 16 min.

### Nuclease resistance assays

Oligonucleotides were prepared at final concentrations of 0.1 mg/ml in 50 mM Tris (pH 7.2), 10 mM MgCl_2_ for assays in the presence of 3′-specific SVPD or in 50 mM sodium acetate (pH 6.5), 10 mM MgCl_2_ for assays in the presence of 5′-specific PDE-II. The exonuclease (75 mU/ml SVPD or 500 mU/ml PDE-II) was added to oligonucleotide solution immediately prior to the first injection onto the HPLC column, and enzymatic degradation kinetics were monitored for 24 h at 25°C. Samples collected over 24 h were immediately injected directly onto a Dionex DNAPac PA200 analytical column at 30°C column temperature. The gradient was from 37% to 52% 1 M NaBr, 10% CH_3_CN, 20 mM sodium phosphate buffer at pH 11 over 10 min with a flow rate of 1 ml/min. The full-length oligonucleotide amount was determined as the area under the curve of the peak detected at *A*_260_. Percent full-length ON was calculated by dividing the area under the curve at a given time point by that at the first time point and multiplying by 100. Activity of enzyme was verified for each experiment by including a 20-mer oligodeoxythymidylate with a terminal PS linkage in each experiment. Each aliquot of enzyme was thawed just prior to the experiment. The half-life was determined by fitting to first-order kinetics. Each degradation experiment was performed in duplicate.

### *In vitro* gene silencing assay

Primary mouse hepatocytes were obtained from Life Technologies and cultured in Williams E Medium with 10% foetal bovine serum. Transfection was carried out by adding 4.9 μl of Opti-MEM plus 0.1 μl of Lipofectamine RNAiMax (Invitrogen) per well to 5 μl of each siRNA duplex at the desired concentration to an individual well in a 384-well plate. The mixture was incubated at room temperature for 20 min, and 40 μl of complete growth media containing 5000 cells was added to the siRNA mixture. Samples were incubated for 24 h, and then RNA was isolated. A similar procedure was followed for the transfection of 10 000 000 cells and scaled accordingly. Dose response experiments were done using eight 6-fold serial dilutions over the range of 20 nM to 75 pM or 50 nM to 187.5 pM.

RNA was isolated using a Dynabeads mRNA Isolation Kit (Invitrogen). Cells were lysed in 75 μl of Lysis/Binding Buffer containing 3 μl of beads per well and mixed for 10 min on an electrostatic shaker. Buffers were prepared according to the manufacturer's protocol. The washing steps were automated on a Biotek EL406 using a magnetic plate support. Beads were washed once in buffer A, once in buffer B, and twice in buffer E, with 90 μl volume per wash and with aspiration steps between washes.

cDNA synthesis was accomplished with the ABI High-capacity cDNA Reverse Transcription kit (Applied Biosystems). A mixture of 1 μl of 10× buffer, 0.4 μl of 25 × dNTPs, 1 μl of random primers, 0.5 μl of reverse transcriptase, 0.5 μl of RNase inhibitor, and 6.6 μl of water per reaction were added per well. Plates were sealed, agitated for 10 min on an electrostatic shaker, and then incubated at 37°C for 2 h. Following this, the plates were agitated at 80°C for 8 min. cDNA (2 μl) was added to a master mix containing 0.5 μl mouse GAPDH TaqMan Probe (Applied Biosystems, Cat.# 4308313), 0.5 μl of mouse TTR or F12 TaqMan probes (Applied Biosystems), and 5 μl of Lightcycler 480 probe master mix (Roche) per well in a 384-well plate (Roche). Real-time PCR was performed in an ABI 7900HT RT-PCR system (Applied Biosystems) using the ΔΔCt (RQ) assay. Each siRNA concentration was tested in four biological replicates. To calculate relative fold change, real-time data were analysed using the ΔΔCt method and normalised to assays performed with cells transfected with 10 nM control siRNA. IC_50_ values were calculated using a four-parameter fit model using XLFit.

### X-ray crystal structure of (*R*)-5′-*C*-methyl-guanosine-containing oligonucleotide

Crystals of 5′-r(CCCCXGGG)-3′ (X = 5′-(*R*)-*C*-methyl-guanosine), which forms a self-complementary duplex, were grown by the hanging-drop vapour diffusion technique using the Nucleic Acid Miniscreen (Hampton Research) as previously described (38). Crystals were obtained from droplets (2 μl) containing 0.6 mM oligonucleotide, 20 mM sodium cacodylate (pH 6.0), 40 mM sodium chloride, 10 mM hexamine cobalt(III) chloride, and 5% (v/v) 2-methyl-2,4-pentanediol (MPD) in a reservoir that was equilibrated against MPD (0.7 ml, 35% (v/v)). The crystals were mounted in nylon loops without further cryo-protection and frozen in liquid nitrogen.

Diffraction data were collected on the 21-ID-D beamline of the Life Sciences Collaborative Access Team at the Advanced Photon Source located at Argonne National Laboratory (Argonne, IL, USA). Data were collected at a wavelength of 0.91833 Å at 100 K using a DECTRISEiger detector. Diffraction data were integrated, scaled, and merged using the program HKL2000 (39). Selected crystal data and data collection parameters are listed in Supplementary Table S4. The crystal with space group *P*6_1_ contains one RNA duplex per asymmetric unit. The structure was phased by molecular replacement with the program MOLREP (40) using a native RNA octamer (PDB ID 259D) as the search model (41). Refinements were carried out in Refmac5 (42,43) keeping aside 5% of the reflections to compute the R-free (44). The duplex model and electron density maps were inspected with the program COOT (45). After three cycles of refinement, methyl groups of (*R*)-5′-*C*-methyl-guanosine residues were built into the electron density, and refinement was continued in Refmac 5 using a dictionary generated using PRODRG (46). Cobalt hexamine and water molecules were added into overlapping positive peaks of Fourier 2*F*_o_ – *F*_c_ sum and *F*_o_ – *F*_c_ difference electron densities and accepted on the basis of standard distance criteria. Final refinement parameters and deviations from ideal geometries are listed in Supplementary Table S4. Coordinates and structure factors for the RNA duplex containing (*R*)-5′-*C*-methyl-guanosine have been deposited in the Protein Data Bank (http://www.rcsb.org) Deposition: D_1000246283; Accession code: PDB ID 6VEM.

### Modelling of exonuclease complexes

Coordinates for the crystal structure of the complex between the *Drosophila melanogaster* 5′-3′ exoribonuclease Xrn1 and a 5′-phosphorylated trinucleotide P-d(TTT) were downloaded from the Protein Data Bank (ID code 2Y35) (47). The 5′-terminal thymine base was replaced by guanine in UCSF Chimera (48) and methyl substituents in the (*R*) or (*S*) configurations were attached to the 5′ carbon of the terminal guanosine to create two models. Both models were energy minimized using the Amber 14 force field in combination with Gasteiger potentials as implemented in UCSF Chimera until changes in the distances between the inserted methyl substituent and nearest neighbours were deemed insignificant.

Coordinates for the crystal structure of the complex between the *Escherichia coli* DNA polymerase I Klenow fragment 3′-5′ exonuclease and a DNA tetramer with a single *S*p-PS moiety 3′-d(T_PS_TTT)-5′ were downloaded from the Protein Data Bank (ID code 1KSP) (49). Methyl substituents in the (*R*) and (*S*) configurations were attached to the 5′ carbon of the 3′-terminal residue to evaluate the distances between the methyl groups and metal ions (Zn^2+^) present at the active site of the exonuclease.

Coordinates for the crystal structure of the complex between the *B. halodurans* RNase H and a 12-mer RNA:DNA hybrid were downloaded from the Protein Data Bank (ID code 1ZBI) (50). Methyl substituents in the (*R*) and (*S*) configurations were attached to the 5′ carbon of the ribonucleotide in the enzyme active site using UCSF Chimera. Both models were energy minimized using the Amber 14 force field in combination with Gasteiger potentials as implemented in the Chimera suite until changes in the distances between the inserted methyl substituent and nearest neighbours were deemed insignificant.

## RESULTS AND DISCUSSION

### Synthesis of (*R*)- and (*S*)-Isomers of 5*′-C-*methyl-guanosine

Beigelman *et al.* used l-rhamnose, which yields a 5′-(*S*)-methyl ribofuranose sugar, as the starting material for the synthesis of 5′-*C*-methyl-guanosine (32). In this strategy, the 5′ position chirality is established by the chirality of the starting material. We synthesized 5′-*C*-methyl-guanosine using our previously reported Grignard alkylation strategy (27). The enantiomerically pure (*R*) and (*S*) isomers of 5′-*C*-methyl-guanosine were obtained using 2′, 3′-*O*-isopropylidene guanosine as starting material (Scheme 1). In this route, the methyl group is introduced on the 5′ position of 2′, 3′-*O*-isopropylidene guanosine using the Grignard methylation reagent. 2′,3′-*O*-Isopropylidene guanosine was treated first with TMSCl and then with isobutyryl chloride in pyridine to yield 2′,3′-*O*-isopropylidene N_2_-isobutyryl guanosine **1**. Dess–Martin oxidation of 2′,3′-*O*-isopropylidene guanosine afforded the unstable aldehyde **2**. Addition of CH_3_MgBr to the crude aldehyde **2** in DCM resulted in a stereoisomeric mixture of the (*R*) and (*S*) isomers **3** and **13**. Treatment of the mixture of **3** and **13** with TBDPSCl and imidazole in DMF afforded the corresponding 5′-*O*-*tert*butyldiphenylsilyl protected 5′-*C*-methyl-guanosines **4** and **14**, respectively (Scheme 1), which were separated by silica gel column chromatography. The separation of **4** and **14** was difficult resulting in the low isolated yields. After removal of acetonide with 80% TFA (aq.), 2′, 3′-diols **5** and **15** were obtained (Scheme 2). The diols were treated with benzoic anhydride in the presence of DMAP in pyridine to yield 2′,3′-*O*-dibenzoyls **6** and **16**, respectively. Deprotection of the 5′-*O*-TBDPS was carried out in 1 M TBAF in THF followed by tritylation in the presence of DMTrCl and AgNO_3_ in pyridine to afford the 5′-DMTr nucleosides **8** and **18**, respectively. The 5′-OH is secondary alcohol, which has low reactivity, and a Lewis acid is necessary for production of the active DMT cation. Treatment of **8** and **18** with 1 M NaOH deprotected the benzoyl and resulted in the 2′,3′-diols **9** and **19**. The free 2′-hydroxy groups were silylated using TBSCl in the presence of AgNO_3_ in THF/pyridine. Finally, 3′-*O*-phosphitylation of **10** and **20** using *i*-Pr_2_NP(Cl)O(CH_2_)_2_CN, DIPEA and 1-methylimidazole in DCM afforded the corresponding phosphoramidites **11** and **21**, respectively. Compounds **10** and **20** were succinylated at the 3′ positions by treatment with succinic anhydride in the presence of DMAP in pyridine to obtain the corresponding hemisuccinates, which were subsequently coupled to CPG support functionalized with LCAA under HBTU-mediated amide coupling conditions to afford the desired solid supports **12** and **22** with loadings of 70 and 97 μmol/g, respectively (Scheme 2).

### Assignment of (*R*) and (*S*) configurations of 5′-*C*-methyl using Mosher ester analysis

**Scheme 3.**
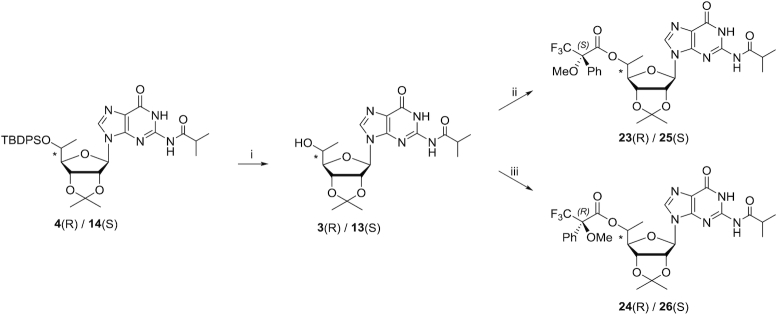
Reagents and conditions: (i) 1 M TBAF in THF/THF, room temperature, overnight; 82%. (ii) (*R*)-(–)-MTPACl/DCM/pyridine, 0°C to room temperature, 2 h; 72%. (iii) (*S*)-(+)-MTPACl/DCM/pyridine, 0°C to room temperature, 2 h; 38%.

**Figure 3.**
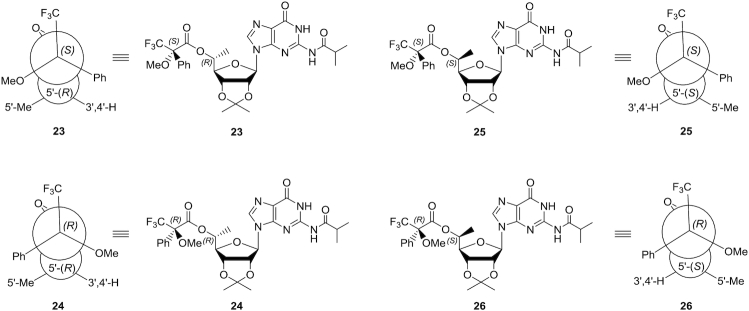
Conformations of the (*S*)- and (*R*)-MTPA ester pairs **23** and **25** and pairs **24** and **26**. Atomic structures and Newman projections are shown.

To determine the absolute configurations of the 5′ positions, we used an NMR-based Mosher ester analysis similar to that described previously (51). We coupled the 5′-hydroxyl groups of pure compounds **3** and **13** separately with (*R*)-(−)-MTPACl and (*S*)-(+)-MTPACl (Scheme 3). This resulted in the formation of Mosher esters **23** and **24** for compound **3** and Mosher esters **25** and **26** for compound **13**. The phenyl substituent of the MTPA ester imposes an anisotropic, magnetic shielding effect on protons residing above and below the plane of the phenyl ring. This shielding results in an upfield chemical shift for the affected protons in the NMR spectrum. The ^1^H NMR chemical shift differences (Δδ*^SR^*) for 1′, 2′, 3′, and 4′ protons and 5′-methyl protons in the Mosher ester pairs were obtained (Table 1). For the Mosher ester pair derived from compound **3** (compounds **23** and **24**) the Δδ*^SR^* values for 1′, 2′, 3′ and 4′ protons were negative, and the Δδ*^SR^* value for the 5′-methyl protons was positive. This result indicates that in compound **23**, the sugar protons are on the same side as the phenyl group, whereas the 5′-methyl group is on the opposite side. In compound **24**, the converse was observed: the 5′-methyl group is on the same side as the phenyl group, whereas the sugar protons are on the opposite side. These data indicate that the parent compound **3** has the 5′-(*R*)-methyl configuration (Figure 3). For the Mosher ester pair derived from compound **13** (compounds **25** and **26**), the Δδ*^SR^* value for the 1′, 2′, 3′ and 4′ protons were positive, and the Δδ*^SR^* value for the 5′-methyl protons was negative. This indicates compound **13** has the 5′-(*S*)-methyl configuration (Figure 3).

**Table 1.** Δδ*^SR^* data for the (*S*)- and (*R*)-MTPA esters of **23**–**26**

	**23**	**24**	Δδ*^SR^* ( = δ*_S_* – δ*_R_*)	
Proton	*d* (*S*)-Mosher ester	*d* (*R*)-Mosher ester	ppm	Hz (400 MHz)
3′H	4.98	5.12	−0.14	−56
4′H	4.03	4.09	−0.06	−24
1′H	6.02	6.07	−0.05	−20
2′H	5.35	5.37	−0.02	−8
5′H	5.24	5.24	0	0
5′Me	1.23	1.13	0.1	40
	**25**	**26**	Δδ*^SR^* ( = δ*_S_* – δ*_R_*)	
Proton	*d* (*S*)-Mosher ester	*d* (*R*)-Mosher ester	ppm	Hz (400 MHz)
3′H	5.24	4.80	0.44	176
4′H	4.10	4.13	−0.03	–12
1′H	6.19	6.03	0.16	64
2′H	5.33	5.08	0.25	100
5′H	5.33	5.29	0.04	16
5′Me	1.26	1.35	−0.09	−36

### Conformational analyses of the sugar rings in 5′-*C*-methyl-guanosine isomers

**Figure 4.**
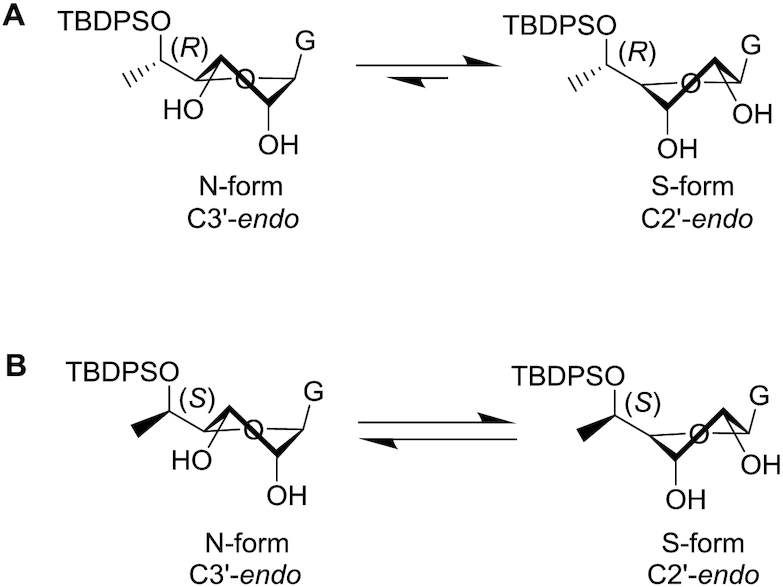
Conformations of 5′-*C*-methyl stereoisomers based on ^1^H NMR. (**A**) Approximate equilibrium between C3′-*endo* and C2′-*endo* conformations for 5′-(*R*)-*C*-methyl-guanosine **5**. (**B**) Approximate equilibrium between C3′-*endo* and C2′-*endo* conformations for 5′-(*S*)-*C*-methyl-guanosine **15**.

The sugar conformations of 5′-*O*-TBDPS-protected 2′,3′-diols of compounds **5** and **15**, the 5′-(*R*)- and 5′-(*S*)-*C-*methyl-guanosines, respectively, were analysed by ^1^H NMR. ^3^*J*_H–H_ couplings were compared with those of 5′-TBS-protected guanosine; the results are summarized in Table 2. We used Altona's empirical formula to calculate the percentage of the furanose rings of the nucleosides that adopt the C3′-*endo* sugar pucker (52). The percentage of C3′-*endo* sugar puckering of the (*S*) isomer was similar to that of the non-methylated guanosine nucleoside determined previously (53), whereas the (*R*) isomer had a lower percentage of C3′-*endo* sugar puckering indicative of a more DNA-like conformation than that of the (*S*) isomer (Figure 4). This agrees with previously reported conformational studies on 5′-*C*-methyl deoxyribonucleoside analogues (27).

**Table 2.** Percent C3′-*endo* conformers of 5′-*O*-TBDPS-protected 2′,3′-diols of 5′-(*R*)- and 5′-(*S*)-*C-*methyl-guanosine based on ^1^H NMR

Compound	5′-*O* protection	5′-*C* modification	^3^ *J*_H1′-H2′_ (Hz)	% C3′ *endo*^a^
Guanosine	TBS	H	5.3	∼50
(*R*) isomer, **5**	TBDPS	methyl	6.6	∼30
(*S*) isomer**, 15**	TBDPS	methyl	5.3	∼50

^a^The percent of molecules in the C3′-*endo* conformation was calculated as (100 – (10 × ^3^*J*_H1′-H2′_)) as previously described (52), where ^3^*J*_H1′–H2′_ is the ^1^H NMR coupling constant of the sugar H1′ and H2′ protons.

### Evaluation of conformations of 5′-(*R*)- and 5′-(*S*)-*C*-methyl-guanosine triphosphates

Nucleoside and nucleotide analogues can have adverse effects on mitochondrial function as triphosphorylated metabolites of these nucleosides have the potential to serve as substrates for DNA and RNA polymerases (54). Modified residues in oligonucleotides can, upon metabolism, lead to reduced mitochondrial DNA copy numbers and mitochondrial dysfunction if they serve as polymerase substrates (55,56). We therefore evaluated whether enantiomerically pure 5′-*C*-methyl-guanosine triphosphates were substrates for mitochondrial RNA polymerase POLRMT or the mitochondrial DNA polymerase POLG.

**Scheme 4.**
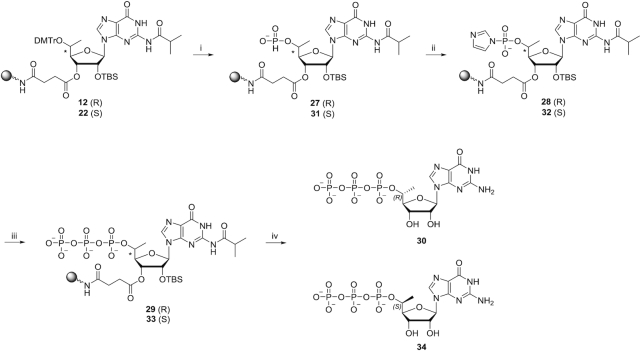
Reagents and conditions: steps (i), (ii) and (iii) were performed on an ABI-394 oligonucleotide synthesizer essentially as described (57),). (iv) 1) NH_4_OH/EtOH, room temperature, overnight. 2) TBAF/THF, room temperature, overnight. 3) AMA/H_2_O, room temperature, overnight; 28% for **30** and 39% for **34**.

We synthesized 5′-triphosphates of 5′-(*R*)- and 5′-(*S*)-*C*-methyl-guanosine on CPG supports (Scheme 4) as described previously (57). The 5′-DMTr groups of compound **12** and compound **22** were removed and *H*-phosphonate groups were introduced using diphenyl phosphite to yield compounds **27** and **31**, respectively. Reaction with imidazole after oxidation of the *H*-phosphonate resulted in intermediates **28** and **32**, which were further converted to the triphosphates **29** and **33** using pyrophosphate as described (58,59). After cleavage from the CPG support and removal of the TBS group using standard procedures, the isobutyryl protecting group was removed using a mixture of ammonia and methylamine at room temperature. This procedure was used to prepared monomer **30** and monomer **34**, the 5′-(*R*)- and 5′-(*S*)-*C*-methyl-guanosine triphosphates, respectively. For comparison purposes, we also synthesized the triphosphate of non-methylated guanosine using the same procedure. The formation of triphosphate was confirmed by ^31^P NMR spectroscopy.

Conformations of the furanose rings of triphosphates **30** and **34** were estimated based on ^1^H NMR ^3^*J*_H1′–H2′_ couplings and were compared with that of non-methylated guanosine (Table 3). The presence of a 5′-triphosphate shifted the equilibrium toward C2′-*endo* sugar puckering by 10% for non-methylated guanosine as well as for the (*S*) isomer of 5′-*C*-methyl guanosine compared to their 5′-*O*-TBDMS compounds. The percentage of sugars in the C3′-*endo* conformation for the (*R*) isomer was about 30% for both phosphorylated and non-phosphorylated forms. The percentage of (*S*) isomer molecules with C3′-*endo* conformation was similar to that of the non-methylated guanosine triphosphate, whereas a lower percentage of the molecules with the (*R*) configuration had a C3′-*endo* conformation.

**Table 3.** Percent C3′-*endo* conformers of 5′ triphosphates of (*R*)- and (*S*)-5′-*C-*methyl-guanosine based on ^1^H-NMR

Compound	5′-*O* group	5′-*C* group	^3^ *J*_H1′-H2′_ (Hz)	% C3′ endo ^a^
Guanosine	Triphosphate	H	6.0	∼40
**30**	Triphosphate	(*R*)-methyl	7.0	∼30
**34**	Triphosphate	(*S*)-methyl	6.1	∼40

^a^The percent of molecules in the C3′-*endo* conformation was calculated as (100 – (10 × ^3^*J*_H1′–H2′_)) as previously described (52), where ^3^*J*_H1′–H2′_ is the ^1^H NMR coupling constant of the sugar H1′ and H2′ protons.

### Crystal structure of an RNA octamer containing 5′-(*R*)-*C*-methyl-guanosine

We previously reported that thermal melting of 12-mer homo- and heteroduplexes containing (*R*) and (*S*) epimers of 5′-*C*-methyl pyrimidines have lower stability than unmethylated counterparts (30). The (*S*) epimers destabilize duplexes to a lesser degree than the corresponding (*R*) epimers. This suggests that the spatial orientation of the 5′-*C-*methyl plays an important role in duplex stability. The thermal melting analysis also revealed that the (*S*) epimer incorporated into DNA was less destabilizing than when an RNA strand was modified, irrespective of whether these strands were paired to DNA or RNA. Conversely, incorporation of the (*R*) epimer was destabilizing with all duplex types.

We also analysed crystal structures of RNA octamer duplexes containing either (*R*) or (*S*) isomers of 5′-*C*-methyl pyrimidines (27). A key insight gained from the crystallographic data is that the presence of the 5′-(*R*)-*C*-methyl group creates short intranucleotide contacts to OP1 and O3′ that apparently cannot be avoided by adjustments of the backbone conformation (i.e. the α *sc*^−^/β *ap*/γ *sc*^+^ torsion angle combination versus the stretched all-*ap* conformation). By contrast, the presence of the (*S*)-*C*-methyl group creates short internucleotide contacts to both O2′ and O3′ from the 5′-adjacent nucleotide. The crystal structure of an RNA duplex with (*S*) epimers demonstrated that these short contacts are avoided when the modified nucleotide adopts the stretched α to γ backbone conformation. Further, slight adjustments of the ζ (around O3′-P) and α (around P-O5′) torsion angles distance the methyl group from O2′ and O3′ of the preceding residue. However, the structure shows that this relief comes with local unstacking, consistent with the reduced stability.

To evaluate the effect of 5′-*C*-methyl guanosine on thermal melting of duplexes and to analyse the effects of the 5′-*C*-methyl modification on the backbone geometry of RNA, we characterized self-complementary RNA octamers, 5′-CCCCXGGG-3′, where X is either 5′-(*R*)- or 5′-(*S*)-*C*-methyl-guanosine. Crystals of the octamer modified with 5′-(*R*)-*C*-methyl-guanosine diffracted to 1.56-Å resolution, and the structure was phased by molecular replacement. Crystals of the octamer modified with 5′-(*S*)-*C*-methyl-guanosine diffracted to ∼1.2-Å resolution but did not yield to phasing by molecular replacement. Attempts to phase the structure by single anomalous dispersion (SAD) using octamers with the first, second, or third C replaced by 5-bromocytidine all failed because crystals of the brominated octamer either could not be grown or diffracted only to low resolution, thereby precluding the use of Br-SAD because of an insufficiently strong anomalous signal.

**Figure 5.**
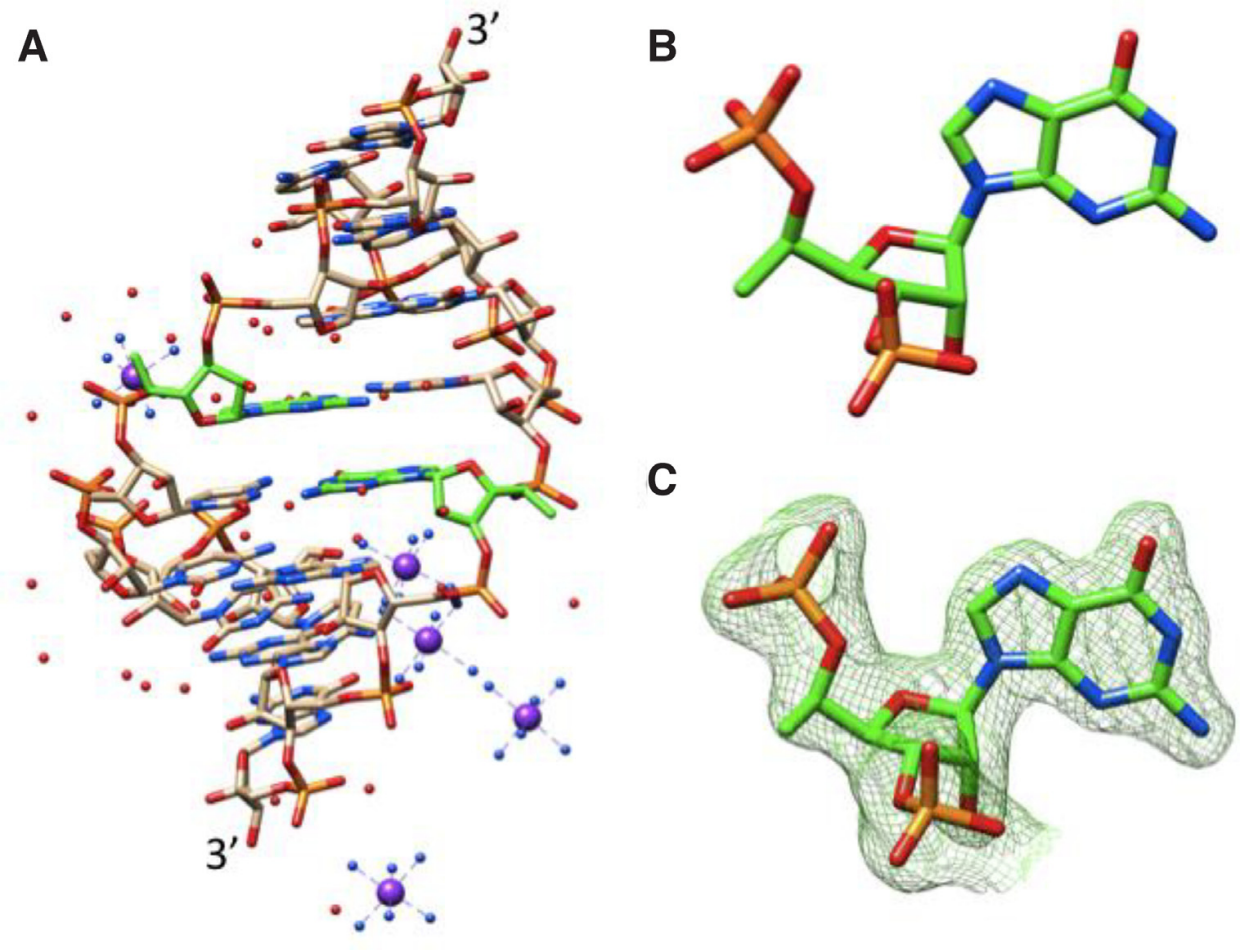
Crystal structure of the RNA octamer duplex with 5′-(*R*)-*C*-methyl-guanosines. (**A**) The duplex viewed into the minor groove. (**B**) Close-up view of 5′-(*R*)-*C*-methyl-guanosine at position 5 of the duplex. (**C**) Close-up view of 5′-(*R*)-*C*-methyl-guanosine at position 13 of the duplex with overlaid Fourier (2*F*o – *F*c) electron density (drawn at ∼1.1σ threshold). Carbon atoms of modified guanosines are highlighted in green, and cobalt ions are depicted as purple spheres.

Refinement of the structure of the octamer containing 5′-(*R*)-*C*-methyl-guanosine yielded a model with a root mean square deviation of 0.7 Å relative to the unmodified octamer (PDB ID: 259D) (41). The two duplexes, excluding the positions of modification, were superimposed using the match option in Chimera (38). In addition to the single RNA duplex per asymmetric crystallographic unit in the hexagonal unit cell, five Co(III) hexamine ions and 30 water molecules were included in the final model. The duplex viewed into the minor groove and close-up views of the two 5′-(*R*)-*C*-methyl-guanosines in the duplex are shown in Figure 5. Selected crystallographic data and data collection and refinement parameters are listed in Supplementary Table S4. The riboses of modified guanosines adopt the C3′-*endo* pucker and the 5′-*C*-methyl groups exhibit short intra-nucleotide 1^…^5 contacts to O3′ and OP1 atoms with distances of between 3.0 and 3.15 Å. The backbone torsion angles α to ζ for the modified guanosines fall into the standard *sc*^−^, *ap*, *sc*^+^, *sc*^+^, *ap* and *sc*^−^ ranges that are characteristic of A-form duplexes. In the reference structure, G13 has an extended backbone as a result of a crankshaft motion around torsion angle β that flips both α and γ into the *ap* range. However, this is not the case with the 5′-(*R*)-*C*-methyl-modified G13. A model with an (*R*)-configured methyl group attached to G13 of the parent duplex (stretched backbone) reveals contacts between methyl carbon and OP2 and guanine C8 of 2.7 and 2.4 Å, respectively. Thus, it appears that short contacts with O3′ and OP1 as a consequence of an (*R*) isomer are unavoidable.

**Figure 6.**
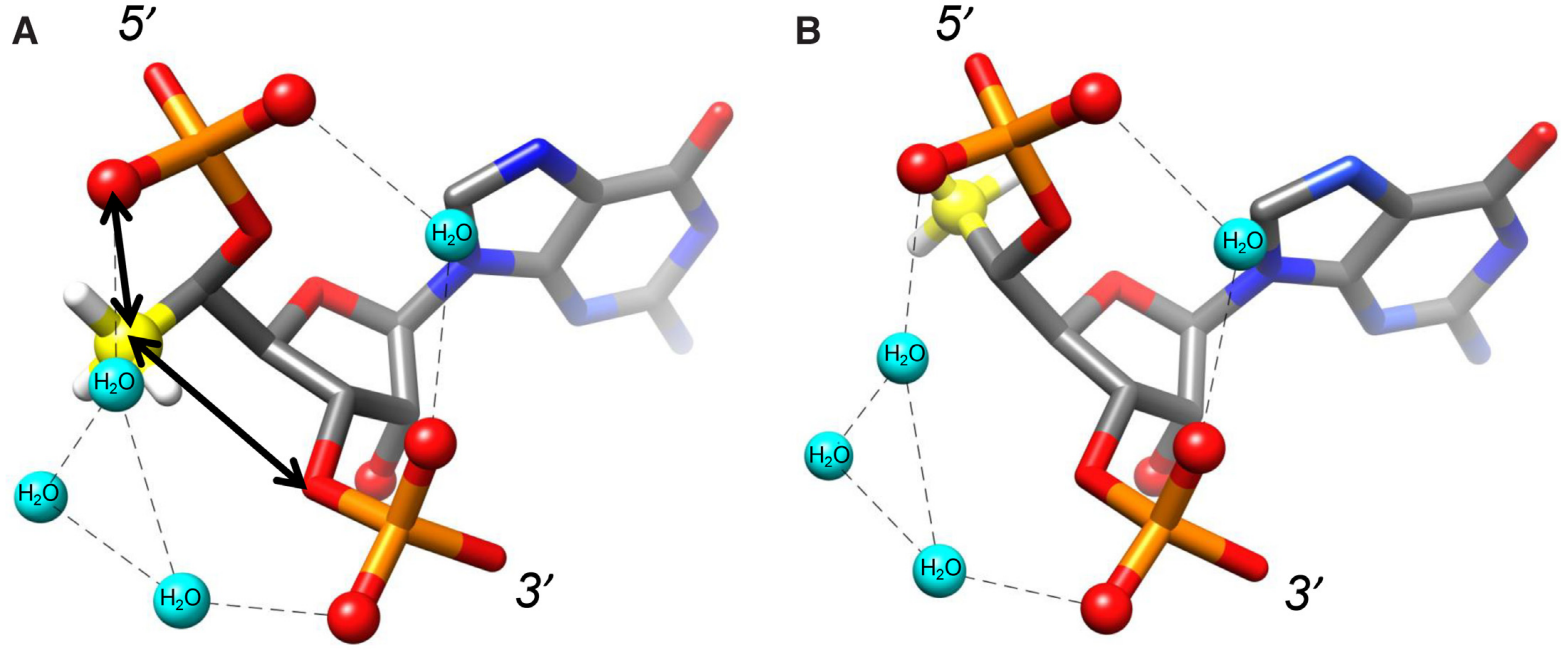
Steric and electrostatic consequences of the (**A**) 5′-(*R*)-*C-*methyl-guanosine and (**B**) 5′-(*S*)-*C-*methyl-guanosine modifications within an RNA duplex. The methyl substituent in the (*R*) configuration produces unfavourable contacts (arrows) and a hydrophobic patch in an electrostatically negatively polarized zone. Thus, the (*R*) methyl group likely interferes with water molecules (cyan spheres) that bridge adjacent phosphate groups. Hydrogen bonds are indicated by dashed lines.

A-form and B-form duplexes exhibit characteristic hydration patterns around the sugar-phosphate backbone as revealed by crystal structures at atomic resolution (60). Previous molecular modelling studies indicate that 5′-(*R*)*-C-* and 5′-(*S*)*-C*-methyl groups perturb the backbone water structure in distinct ways (60). The 5′-(*R*)-*C*-methyl interferes with hydration between adjacent phosphate groups (Figure 6A). In contrast, the 5′-(*S*)-*C*-methyl is directed away from the hydration network around the phosphate backbone (Figure 6B), consistent with the greater loss in duplex thermal stability previously reported for incorporation of the (*R*) isomer compared with the (*S*) isomer (27).

### Analysis of use of 5′-(*R*)- and 5′-(*S*)-*C*-methyl-guanosine triphosphates as substrates for mitochondrial RNA and DNA polymerases

**Figure 7.**
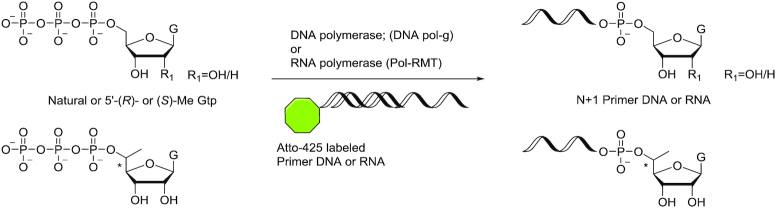
Schematic of polymerization assay. Nucleoside triphosphates were added to a solution containing template and Atto-425-labeled primer and polymerase. The amount of primer extension relative to unreacted primer was evaluated using analytical IEX-HPLC with fluorescence detection.

We next evaluated whether the modified nucleotide triphosphates could serve as substrates for polymerases using assays with Atto-425 labeled primers in a previously described single-nucleotide incorporation assay (17) (Figure 7). The control triphosphates of 5′-non-methylated guanosine and deoxyguanosine were efficiently incorporated into RNA by POLRMT and into DNA by POLG polymerase at 100 μM and 1 mM concentrations, respectively. Neither 5′-(*S*)- nor 5′-(*R*)-*C*-methyl-guanosine triphosphate was recognized as a substrate by the DNA polymerase POLG even at 1 mM concentration (Supplementary Figure S1). As the 5′-(*R*)-*C*-methyl-guanosine triphosphate had less of the RNA character as shown by the % C3′-endo population than guanosine triphosphate (Table 3), it was comforting to know it is still not a substrate for DNA polymerase POLG. The 5′-(*S*)-*C*-methyl-guanosine was incorporated into RNA by POL-RMT at 1mM concentration and to a small extent at 100uM concentration; 5′-(*R*)-*C*-methyl-guanosine triphosphate was not incorporated into RNA at either concentration tested (Supplementary Figure S2). This suggests that the configuration of the 5′ substitution influences the incorporation of nucleoside analogues by mitochondrial polymerases. The crystal structure of human POLRMT elongation complex with a nine base-pair duplex between DNA template and RNA transcript has been determined (PDB ID: 4BOC) (61). Future modelling efforts may shed light on the differences between the two isomers in terms of incorporation by POLRMT.

### Impact of 5′-*C*-methyl-guanosine on nuclease resistance

**Figure 8.**
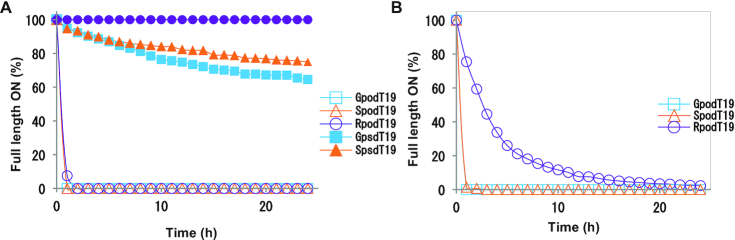
HPLC analysis of degradation of 5′-terminally modified oligonucleotides (0.1 mg/ml) in the presence of (**A**) 500 mU/ml and (**B**) 100 mU/ml PDE-II in 50 mM sodium acetate (pH 6.5) and 10 mM MgCl_2_.

To evaluate the effects of enantiomerically pure 5′-(*R*)- and 5′-(*S*)-*C*-methyl-guanosines on 5′-exonuclease digestion of oligonucleotides, 20-mer oligodeoxythymidylate oligonucleotides were synthesized with a single modified residue at the 5′ end linked via either a phosphodiester (PO) linkage (*R*_PO_dT_19_ or *S*_PO_dT_19_) or a PS linkage (*R*_PS_dT_19_ and *S*_PS_dT_19_). The control oligonucleotides had a single unmodified guanosine at 5′ end (G_PO_dT_19_ and G_PS_dT_19_). Oligonucleotides were incubated with PDE-II, and the half-lives (*t*_1/2_) were determined by HPLC-based quantification of the full-length oligonucleotide as a function of time. The results are summarized in Table 4, and HPLC traces are shown in Figure 8. In the presence of 500 mU/ml of PDE-II, all the oligonucleotides with PO linkages were degraded by the first time point. To enable differences in stability to be distinguished, the oligonucleotides were incubated in the presence of 100 mU/ml of PDE-II enzyme. Oligonucleotides with the PO linkages G_PO_dT_19_ and *S*_PO_dT_19_ were degraded by the first time point. *R*_PO_dT_19_ had a *t*_1/2_ of 4.3 h. Oligonucleotides with a PS linkage were more stable: There was no degradation of *R*_PS_dT_19_ at 24 h even in the presence of 500 mU/ml of PDE-II. The *t*_1/2_ values for the control G_PS_dT_19_ and for the S_PS_dT_19_ were 41 and 72 h, respectively.

**Table 4.** Half-lives of oligonucleotides modified at the 5′ termini with 5′-(*R*)- or 5′-(*S*)-*C*-methyl-guanosines in the presence of PDE-II

	500 mU/ml PDE-II	100 mU/ml PDE-II
Oligonucleotide	*t*_1/2_ (h)	*t*_1/2_ (h)
G_PO_dT_19_	<1	0.1
*S*_PO_dT_19_	<1	0.2
*R*_PO_dT_19_	<1	4.3
G_PS_dT_19_	41	ND
*R*_PS_dT_19_	No degradation	ND
*S*_PS_dT_19_	72	ND

**Figure 9.**
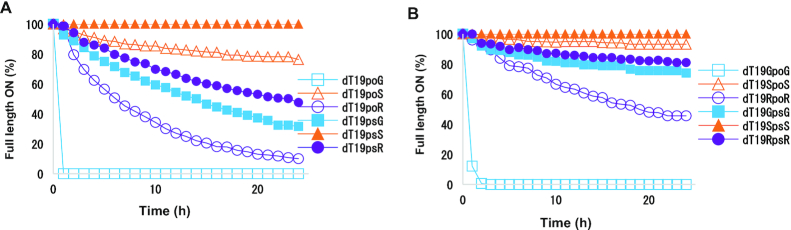
HPLC analysis of degradation of oligonucleotides (0.1 mg/ml) 3′-terminally modified with (**A**) one or (**B**) two non-methylated guanosines or 5′-(*R*)- or 5′-(*S*)-*C*-methyl-guanosines in the presence of 75 mU/ml SVPD in 50 mM Tris (pH 7.2) and 10 mM MgCl_2_.

To evaluate the stability of oligonucleotides with 3′-terminal 5′-(*R*)- or 5′-(*S*)-*C*-methyl-guanosines to 3′ exonuclease, 20-mer oligodeoxythymidylate oligonucleotides modified at the 3′ end with either one or two modified nucleotides linked via PO or PS were synthesized; control oligonucleotides had one or two unmodified guanosine at the 3′ end. Oligonucleotides were incubated with SVPD, and the *t*_1/2_ values were determined. The results are summarized in Table 5, and HPLC traces are shown in Figure 9. When the 3′ terminus was a single guanosine attached via a PO linkage, the oligonucleotide modified was degraded within 1 h. The oligonucleotide modified with the (*S*) isomer (dT_19PO_*S*) was more stable than that modified with the (*R*) isomer (dT_19PO_*R*) with *t*_1/2_ values of 70.7 and 7.1 h, respectively. Replacing the PO linkage with a PS linkage significantly increased resistance to SVPD-mediated cleavage, but the stability trend was the same. The *t*_1/2_ values for control dT_19PS_G and dT_19PS_*R* were 14.4 and 22.6 h, respectively, whereas dT_19PS_*S* was not degraded at all even at the 24 h time point. Attachment of two 5′-*C*-methyl-guanosines at 3′ end of the oligonucleotide linked through either PO or PS resulted in a remarkable increase in resistance to SVPD-mediated degradation relative to singly modified oligonucleotides. The control dT_18_G_PO_G was degraded within 1 h. The oligonucleotide modified with two (*S*)-5′-*C*-methyl-guanosine residues was very stable with an estimated *t*_1/2_ value of 260 h. The oligonucleotide modified with two (*R*)-5′-*C*-methyl-guanosine residues had a *t*_1/2_ value of 20.8 h. These data demonstrate that (*R*)-5′-*C*-methyl-guanosine imparts considerable resistance to 5′ exonuclease, whereas the (*S*) isomer confers stability toward 3′ exonucleases.

**Table 5.** Half-lives of oligonucleotides modified at the 3′ termini with 5′-(*R*)- or 5′-(*S*)-*C*-methyl-guanosines in the presence of 75 mU/ml SVPD

Oligonucleotide	*t*_1/2_ (h)	Oligonucleotide	*t*_1/2_ (h)
dT_19PO_G	<1	dT_18_G_PO_G	<1
dT_19PO_*S*	70.7	dT_18_*S*_PO_*S*	260
dT_19PO_*R*	7.1	dT_18_*R*_PO_*R*	20.8
dT_19PS_*G*	14.4	dT_18_G_PS_G	65.5
dT_19PS_*S*	No degradation	dT_18_*S*_Ps_*S*	No degradation
dT_19PS_*R*	22.6	dT_18_*R*_PS_*R*	89

### Structural basis for nuclease resistance conferred by 5′-*C*-methyl isomers

**Figure 10.**
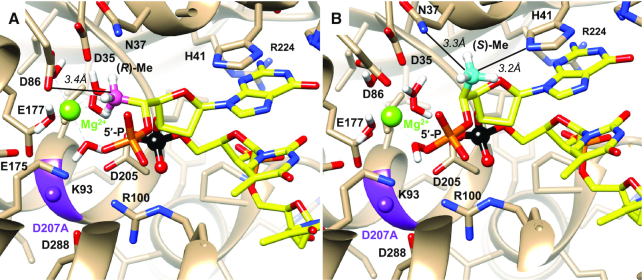
Crystal structure of the *D. melanogaster* Xrn1 5′ exonuclease active site bound to 5′-P-d(TTT)-3′ in which the 5′-terminal base was replaced with (**A**) 5′-(*R*)-*C*-methyl-guanine (pink) and (**B**) 5′-(*S*)-*C*-methyl-guanine (cyan). Models were energy minimized. Selected side chains are labeled, a bound Mg^2+^ is shown as a green sphere, the position of D207, which was mutated to alanine to inactivate the nuclease by preventing binding of the second metal ion to facilitate crystallization, is indicated in purple, and the scissile phosphate is highlighted in black. The shortest distances between methyl carbons and side chain atoms are shown as thin solid lines.

The (*R*) isomer provided better protection than the (*S*) isomer from 5′ exonuclease, whereas the (*S*) isomer provided better protection than (*R*) isomer from 3′ exonuclease. To gain insight into these chirality-dependent protection patterns, we turned to crystal structures of oligonucleotide complexes of Xrn1 5′ exonuclease (47) and DNA polymerase I Klenow fragment 3′ exonuclease (KF exo) (49). In both complexes, we constructed models in which 5′-*C*-methyl substituents were added to the terminal nucleotides to assess potential steric and electrostatic conflicts with protein side chains and metal ions bound at the active site. These models allowed a qualitative rationalization for experimentally observed differences afforded by 5′-(*R*)- and 5′-(*S*)-*C*-methyl-guanosines. The 5′-(*R*)-methyl substituent at the active site of Xrn1 5′ exonuclease is directed toward the metal ion binding site and will most likely interfere with metal ion binding (Figure 10A). In contrast, the 5′-(*S*)-methyl substituent is directed away from side chains involved in metal ion coordination (Figure 10B). Thus, the structural data are consistent with the protection from 5′ exonuclease afforded by the 5′-(*R*)-methyl-guanosine at the 5′ end of the RNA.

**Figure 11.**
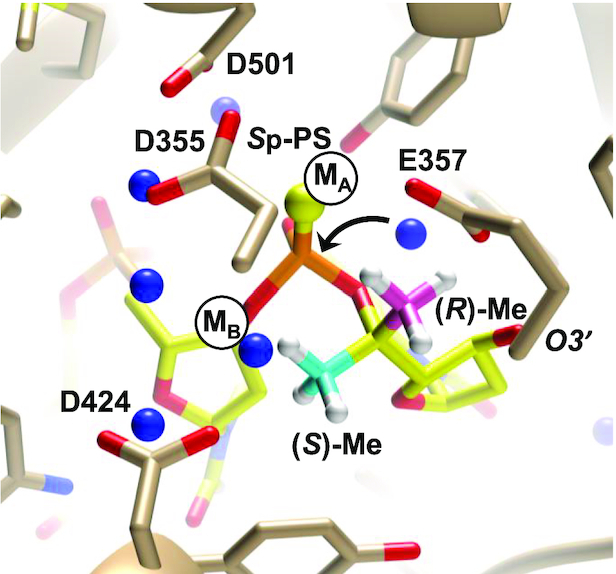
Crystal structure of the KF exo active site bound to 3′-d(T_PS_TTT)-5′, where PS indicates an *S*p phosphorothioate linkage in which 5′-(*R*)-*C-*methyl (pink) and 5′-(*S*)-*C*-methyl (cyan) groups were inserted on the 3′-terminal nucleotide. Positions of Zn^2+^ ions in the crystal structure of KF exo with native DNA but absent in the structure with a *S*p-PS-modified oligo are indicated by black circles labeled *M*_A_ and *M*_B_, water molecules are blue spheres, and an arrow indicates attack by a water molecule at the 3′-terminal phosphate group.

Based on models of KF exo:DNA complexes in which 5′-(*R*)- and 5′-(*S*)-*C*-methyl substituents were incorporated on the 3′-terminal nucleotide of the crystal structure, it is clear that there is a shorter distance from the (*S*)-*C*-methyl group to one of the active site metal ions than there is from the (*R*)-*C*-methyl group (Figure 11). The distance between the (*S*)-*C*-methyl carbon and the metal ion is ∼3 Å. The (*R*)-*C*-methyl group points in the direction of the *S*p sulfur atom of the PS linkage and thus likely interferes with binding of the metal ion. This is consistent with our observations that a combination of a phosphorothioate linkage and the 5′-(*S*)-*C*-methyl modification effectively blocks 3′-exonuclease cleavage (Table 5, Figure 9). We previously reported that modifications that directly interfere with or prevent binding of either catalytically essential metal ion protect oligonucleotides against degradation by KF exo (62).

**Figure 12.**
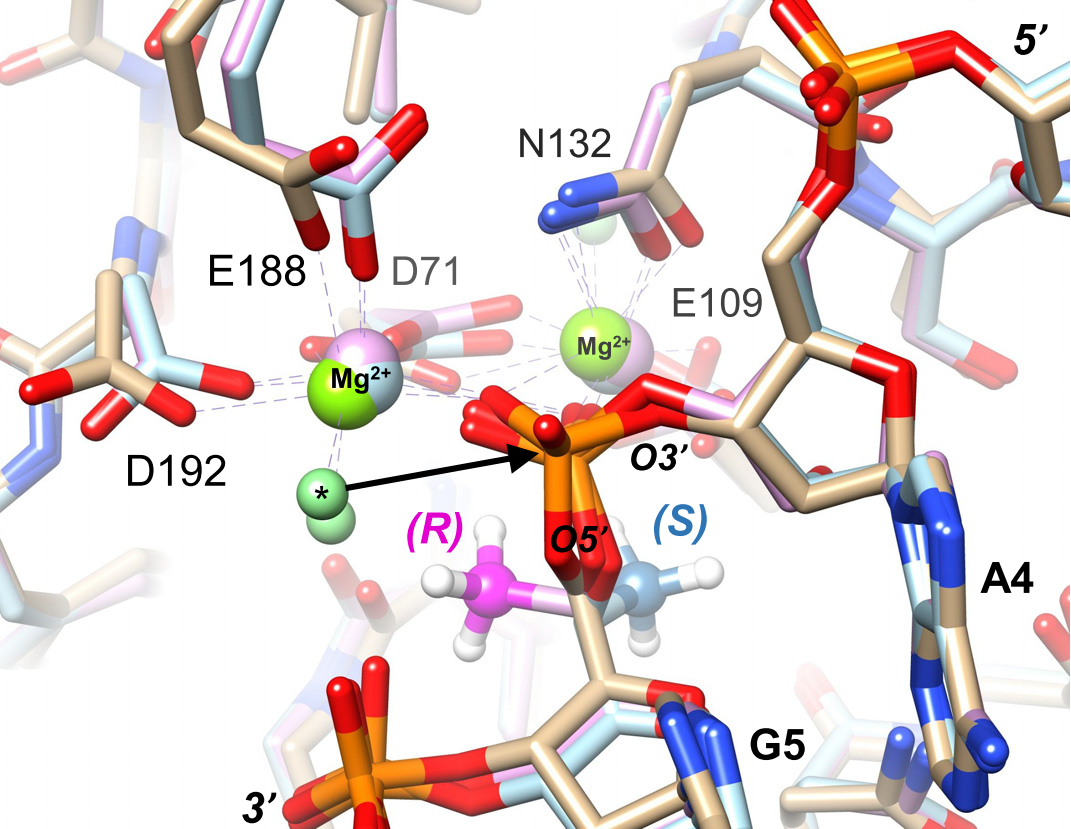
Model of effect of 5′-*C*-methylation on endoribonuclease action. Views of the active sites in the crystal structure of *B. halodurans* RNase H bound to an RNA:DNA hybrid duplex (PDB ID: 1ZBI). The enzyme carbon atoms are in beige. Metal ion A, foreground, and metal ion B, background, are shown as light green spheres. Selected protein side chains are labeled. The D132N mutation prevents cleavage of the RNA in the experimentally determined structure. The coordination spheres of Mg^2+^ ions are indicated by dashed lines, and a water molecule coordinated to metal ion A and poised for attack at the phosphate is marked by an asterisk. In the model of the complex with 5′-(*R*)-*C-*methyl modification, the carbon atoms are pink, and the methyl carbon is highlighted in magenta. In the model with 5′-(*S*)-*C-*methyl modification, the carbon atoms are in light blue, and the methyl carbon is highlighted in darker blue.

We further examined the effects of the 5′-*C*-methyl modification on the action of an RNA endonuclease by modelling *Bacillus halodurans* RNase H in complex with an RNA:DNA hybrid with either 5′-(*R*)-*C*-methyl-guanosine or 5′-(*S*)-*C*-methyl-guanosine at the active site (Figure 12). In both cases, the presence of the methyl group results in a reorientation of the phosphate group with concomitant alterations in the positions of aspartate and glutamate side chains that are coordinated to the two metal ions (Figure 12). RNase H cleaves the RNA strand of an RNA:DNA duplex to yield products with a free 3′-hydroxyl and a 5′-phosphate group. A misaligned phosphate at the active site as a result of steric interference by the 5′-*C*-methyl moiety is expected to hamper activity. The 5′-(*R*)-*C*-methyl group is positioned halfway between metal ions A and B (distances of 4.0 and 4.1 Å, respectively). By comparison, the 5′-(*S*)-*C*-methyl group sits closer to metal ion B than to metal ion A (distances of 3.6 and 5.5 Å, respectively). Both methyl groups drastically alter the electrostatic environment of the phosphate and given their proximity to Mg^2+^ in the models, it is likely that they will promote displacement of either one or both metal ions resulting in resistance to cleavage by the endonuclease.

### *In vitro* siRNA efficacy

We investigated the impact of 5′-(*R*)- and 5′-(*S*)-*C*-methyl-guanosine on the activity of an siRNA transfected into cells in culture. siRNAs tested were designed to target either the mouse *TTR* mRNA, which encodes transthyretin, or the mouse *F12* mRNA, which encodes the coagulation factor FXII. The sequences and chemical modifications in addition the 5′-(*S*)-*C*-methyl guanosine were those previously described (3,5,7–9,16). The siRNAs were transfected into primary mouse hepatocytes using Lipofectamine RNAiMAX, and mRNA levels were quantified by RT-PCR. IC_50_ values are listed in Table 6. The 5′-(*R*)-*C*-methyl-guanosine substitutions at positions 5 and 7 of the sense strand of the *TTR*-targeted siRNA resulted in slight changes in gene silencing activity compared to that of the parent siRNA. The (*S*) isomer also slightly improved the potency when incorporated at either of these positions. In the antisense strand of *TTR*-targeted siRNA, incorporation of the (*S*) isomer at either position 6 or 8 resulted in no change in potency relative to the parent siRNA. Incorporation of the (*R*) isomer at position 8 enhanced potency, but incorporation at position 6 compromised activity slightly compared to the activity of the parent, although pM activity was maintained. When incorporated into the sense strand of the *F12*-targeted siRNA, neither isomer altered potency to a large extent. That 5′-*C*-methyl modifications had little effect on gene silencing indicates that the 2′-OH-mediated interactions with Ago2 are not disrupted by this modification. Thus, despite minor perturbations of hydration observed in the crystal structures (Figure 6), these experiments demonstrate that the 5′-*C*- methyl-guanosine does not cause distortions to the nucleobase or to the normal *anti* configuration that alter interactions with Ago2.

**Table 6.** mTTR (top) and F12 (bottom) parent sequences with chemical modifications and IC_50_ values for 5′-(*R*)- and 5′-(*S*)-*C*-methyl-guanosine modified siRNAs in cell-based *mTTR* and *F12* silencing assays

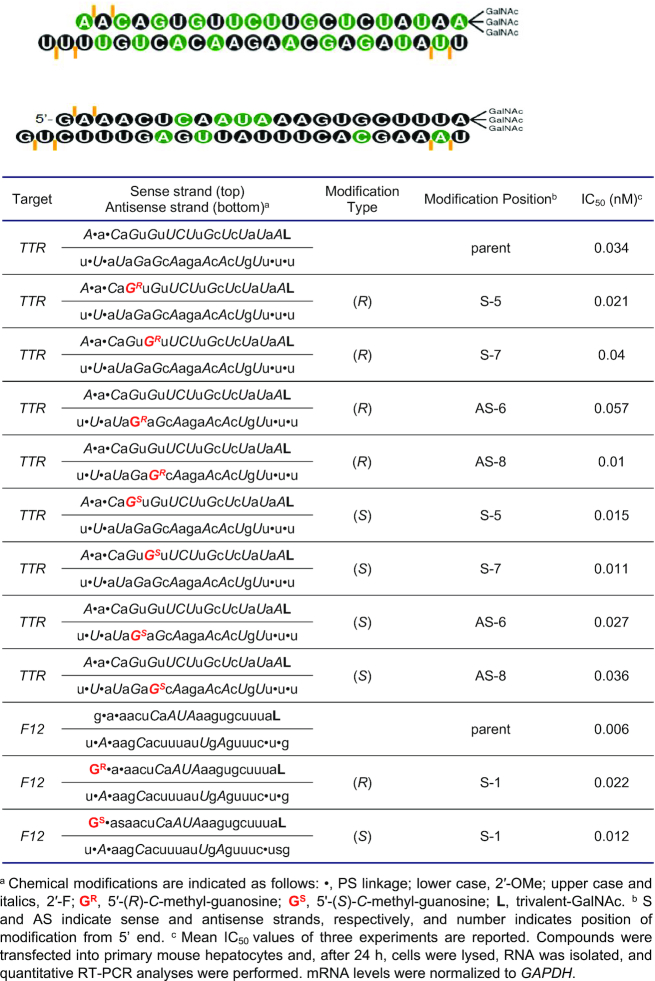

## CONCLUSIONS

Chemical modification of siRNAs used in RNAi-based applications is necessary for metabolic stability and for tissue delivery. Our group has evaluated a number of ribosugar modifications to optimize the interaction of the siRNA with Ago2, the catalytic component of the RISC. It is important to protect such modifications from nucleolytic degradation and to ascertain the safety of such chemical modifications. We developed an efficient route for the synthesis of 5′-(*R*)- and 5′-(*S*)-*C*-methyl-guanosine, starting from the nucleoside guanosine, and evaluated interactions of RNA oligonucleotides containing these monomers with polymerases, nucleases and for RNAi activity.

1. Neither isomer was recognized by the DNA polymerase POLG. The 5′-(*S*)-*C*-methyl-guanosine triphosphate did serve as a substrate for human mitochondrial RNA polymerase POLRMT, although only at 1 mM concentration but not at 100 μM concentration.
2. Terminal modification with (*R*) and (*S*) isomers had opposite effects on resistance of oligonucleotides to 3′ and 5′ exonucleases: the (*R*) isomer provided better protection than (*S*) isomer from 5′ exonuclease, whereas the (*S*) isomer provided better protection than (*R*) isomer from 3′ exonuclease.
3. Analysis of *in vitro* gene silencing by siRNAs modified at a single position with 5′-(*S*)- or 5′-(*R*)-*C*-methyl-guanosine demonstrated that neither isomer interfered with the RNAi machinery at the positions tested.
4. These biological properties were rationalized based on crystal structures of RNA octamers containing 5′-(*S*)- or 5′-(*R*)-*C*-methyl-guanosine and available enzyme structures.

To fully understand the therapeutic potential of the modification, evaluation of siRNAs containing all the four 5′-*C*-methyl-modified ribonucleotides will be necessary, and experiments toward this goal are in progress. Modifications of the guanosine ribonucleoside is expected to be the most synthetically challenging among the nucleosides and the present study has addressed this challenge. The chirality-dependant properties of 5′-(*S*)- and 5′-(*R*)-*C*-methyl-guanosine observed here may also be useful in other nucleoside-, nucleotide- and nucleic acid-based therapeutic applications beyond RNAi. For example, cyclic dinucleotides composed of guanosine and adenosine linked via two phosphodiester linkages can activate the cGAS-STING pathway to induce production of type I interferon and other immune-modulatory cytokines (63,64). These cyclic dinucleotides have the potential for use in therapies to control viral, bacterial, metabolic and oncology-related diseases. The synthetic route developed here will facilitate evaluation of dinucleotides containing 5′-(*S*)- and 5′-(*R*)-*C*-methyl-guanosine as antagonists of the cGAS-STING pathway as well.

## DATA AVAILABILITY

Coordinates and structure factors for the octamer RNA duplex have been deposited in the Protein Data Bank (http://www.rcsb.org.) and the PDB Accession ID code is 6VEM. Deposition: D_1000246283.

## Supplementary Material

gkaa750_Supplemental_File

## Notes

Present address: Atsushi Mikami, Bioorganic Chemistry, Graduate School of Pharmaceutical Sciences, Osaka University 1-6 Yamadaoka, Suita, Osaka 565-0871, Japan.
