# Supplementary material for: Synthesis, chirality-dependent conformational and biological properties of siRNAs containing 5′-(R)- and 5′-(S)-C-methyl-guanosine
Source: Nucleic Acids Res. 2020 Sep 29;48(18):10101–24. doi: 10.1093/nar/gkaa750 (PMC7544225; doi:10.1093/nar/gkaa750)

Supplementary Material

Synthesis, Chirality-dependent Conformational and Biological Properties of siRNAs Containing 5'-(*R*)- and 5'-(*S*)-*C*-Methyl-Guanosine

Atsushi Mikami[1,2], Namrata Erande[1], Shigeo Matsuda[1], Alexander Kel'in, Lauren Blair Woods[1], Tyler Chickering[1], Pradeep S. Pallan[3], Mark K. Schlegel[1], Ivan Zlatev[1], Martin Egli[3,] *, and Muthiah Manoharan[1,]*

[1] Alnylam Pharmaceuticals, 300 Third Street, Cambridge, Massachusetts 02142, USA, [2]Current Address: Bioorganic Chemistry, Graduate School of Pharmaceutical Sciences, Osaka University 1-6 Yamadaoka, Suita, Osaka 565-0871, Japan, [3] Department of Biochemistry Vanderbilt University, School of Medicine Nashville, TN 37232, USA

*To whom correspondence should be addressed. Tel: 617-551-8319; Email: mmanoharan@alnylam.com; martin.egli@vanderbilt.edu

CONTENT

- **Supplementary Table 1.** MS (*m/z*) analysis of modified oligonucleotides for exonuclease assay
- **Supplementary Table 2.** MS (*m/z*) analysis of modified oligonucleotides for *In Vitro* assay
- **Supplementary Table 3.** Primer and template sequences used in the mitochondrial polymerase incorporation and inhibition assays.
- **Supplementary figure 1:** Incorporation of native and 5′-*C*-methyl guanosine triphosphates by POLRMT *in vitro*.
- **Supplementary Figure 2:** Incorporation of native and 5′-*C*-methyl guanosine triphosphates by POLG *in vitro*.
- **Supplementary Table 4.** Selected crystal data, data collection and refinement parameters.
- **^{1}H, ^{13}C and ^{31}P NMR spectra for the new compounds**

Supplementary Table 1. MS (*m/z*) analysis of modified oligonucleotides for exonuclease assay

Sr. No.	Code	[a]Sequence	Mass Calculated	Mass Observed
1	$G_{PO}dT_{19}$	GdTdTdTdTdTdTdTdTdTdTdTdTdTdTdTdTdTdTdT	6062.9	6076.9
2	$S_{PO}dT_{19}$	G^{S}dTdTdTdTdTdTdTdTdTdTdTdTdTdTdTdTdTdTdT	6076.9	6076.3
3	$R_{PO}dT_{19}$	G^{R}dTdTdTdTdTdTdTdTdTdTdTdTdTdTdTdTdTdTdT	6076.9	6076.3
4	$G_{PS}dT_{19}$	G●dTdTdTdTdTdTdTdTdTdTdTdTdTdTdTdTdTdTdT	6078.9	6078.4
5	$R_{PS}dT_{19}$	G^{R}●dTdTdTdTdTdTdTdTdTdTdTdTdTdTdTdTdTdTdT	6093.0	6092.4
6	$S_{PS}dT_{19}$	G^{S}●dTdTdTdTdTdTdTdTdTdTdTdTdTdTdTdTdTdTdT	6093.0	6092.4
7	$dT_{19PO}G$	dTdTdTdTdTdTdTdTdTdTdTdTdTdTdTdTdTdTdTG	6062.9	6062.3
8	$dT_{19PO}S$	dTdTdTdTdTdTdTdTdTdTdTdTdTdTdTdTdTdTdTG^{S}	6093.0	6092.4
9	$dT_{19PO}R$	dTdTdTdTdTdTdTdTdTdTdTdTdTdTdTdTdTdTdTG^{R}	6093.0	6092.4
10	$dT_{19PS}G$	dTdTdTdTdTdTdTdTdTdTdTdTdTdTdTdTdTdTdT●G	6078.9	6080.3
11	$dT_{19PS}S$	dTdTdTdTdTdTdTdTdTdTdTdTdTdTdTdTdTdTdT●G^{S}	6093.0	6092.3
12	$dT_{19PS}R$	dTdTdTdTdTdTdTdTdTdTdTdTdTdTdTdTdTdTdT●G^{R}	6093.0	6092.3
13	$dT_{18}G_{PO}G$	dTdTdTdTdTdTdTdTdTdTdTdTdTdTdTdTdTdTGG	6103.9	6103.3
14	$dT_{18}S_{PO}S$	dTdTdTdTdTdTdTdTdTdTdTdTdTdTdTdTdTdT$G^{S}G^{S}$	6131.9	6131.4
15	$dT_{18}R_{PO}R$	dTdTdTdTdTdTdTdTdTdTdTdTdTdTdTdTdTdT$G^{R}G^{R}$	6131.9	6131.4
16	$dT_{18}G_{PS}G$	dTdTdTdTdTdTdTdTdTdTdTdTdTdTdTdTdTdTG●G	6119.9	6119.3
17	$dT_{18}S_{PS}S$	dTdTdTdTdTdTdTdTdTdTdTdTdTdTdTdTdTdTG^{S} ●G^{S}	6148.0	6147.4
18	$dT_{18}R_{PS}R$	dTdTdTdTdTdTdTdTdTdTdTdTdTdTdTdTdTdTG^{R} ●G^{R}	6148.0	6147.4

[a] Modifications are indicated as follows: •, phosphorothioate (PS) linkage; dT, deoxy thymidine; **G^{R}**, 5′-(*R*)-*C*-methyl-guanosine; and **G^{S}**, (*S*)-*C*-methyl-guanosine.

Supplementary Table 2. MS (*m/z*) analysis of modified oligonucleotides for *in vitro* assay

Sr.No.	Target	Sense strand (top)<br>Antisense strand (bottom)[a]	Modification position[b]	Calculated Mass	Observed Mass
Si-1	*TTR*	A•a•CaGuGuUCUuGcUcUaUaA**L**		8590.2	8590.3
		u•U•aUaGaGcAagaAcAcUgUu•u•u		7595.9	7597.6
Si-2	*TTR*	A•a•CaG^{R}uGuUCUuGcUcUaUaA**L**	S-5	8602.2	8602.8
		u•U•aUaGaGcAagaAcAcUgUu•u•u		7595.9	7597.7
Si-3	*TTR*	A•a•CaGuG^{R}uUCUuGcUcUaUaA**L**	S-7	8602.2	8602.9
		u•U•aUaGaGcAagaAcAcUgUu•u•u		7595.9	7596.9
Si-4	*TTR*	A•a•CaGuGuUCUuGcUcUaUaA**L**		8590.2	8590.7
		u•U•aUaG^{R}aGcAagaAcAcUgUu•u•u	AS-6	7608	7609.1
Si-5	*TTR*	A•a•CaGuGuUCUuGcUcUaUaA**L**		8590.2	8590.8
		u•U•aUaGaG^{R}cAagaAcAcUgUu•u•u	AS-8	7608	7608.8
Si-6	*TTR*	A•a•CaG^{S}uGuUCUuGcUcUaUaA**L**	S-5	8602.2	8603.2
		u•U•aUaGaGcAagaAcAcUgUu•u•u		7595.9	7597.5
Si-7	*TTR*	A•a•CaGuG^{S}uUCUuGcUcUaUaA**L**	S-7	8602.2	8603
		u•U•aUaGaGcAagaAcAcUgUu•u•u		7595.9	7596.5
Si-8	*TTR*	A•a•CaGuGuUCUuGcUcUaUaA**L**		8590.2	8591
		u•U•aUaG^{S}aGcAagaAcAcUgUu•u•u	AS-6	7608	7608.2
Si-9	*TTR*	A•a•CaGuGuUCUuGcUcUaUaA**L**		8590.2	8591.3
		u•U•aUaGaG^{S}cAagaAcAcUgUu•u•u	AS-8	7608	7608.4
Si-10	*F12*	g•a•aacuCaAUAaagugcuuua**L**		8756.6	8757.3
		u•A•aagCacuuuauUgAguuuc•u•g		7610	7610.8
Si-11	*F12*	G^R•a•aacuCaAUAaagugcuuua**L**	S-1	8756.6	8757.4
		u•A•aagCacuuuauUgAguuuc•u•g		7610	7610.6
Si-12	*F12*	G^S•asaacuCaAUAaagugcuuua**L**	S-1	8756.6	8757.3
		u•A•aagCacuuuauUgAguuuc•usg		7610	7611

[a] Chemical modifications are indicated as follows: •, PS linkage; lower case, 2′-OMe; upper case and italics, 2′-F; **G^R**, 5′-(*R*)-*C*-methyl-guanosine; **G^S**, 5′-(*S*)-*C*-methyl-guanosine; **L**, trivalent GalNAc. [b] S and AS indicates sense and antisense strand, and number indicates position of modification from 5′ end. ; **L** indicate tri-GalNAc it is hydroxyprolynyl trivalent N-acetyl galactosamine ligand.[16]

Supplementary Table 3. Primer and template sequences used in the mitochondrial polymerase incorporation and inhibition assays.

		POLRMT	POLG
Primer		5'-Atto-425-UUUUGCCGCGCC-3'	5'-Atto-425-d[CGATATTCACAAAG]-3'
**Template**	**G+**	5'-d[GGGAATGTACGGCGCGGC]-3'	5'-d[CATGCTCTAACCCGCCTTTGTGAATATCG]-3'

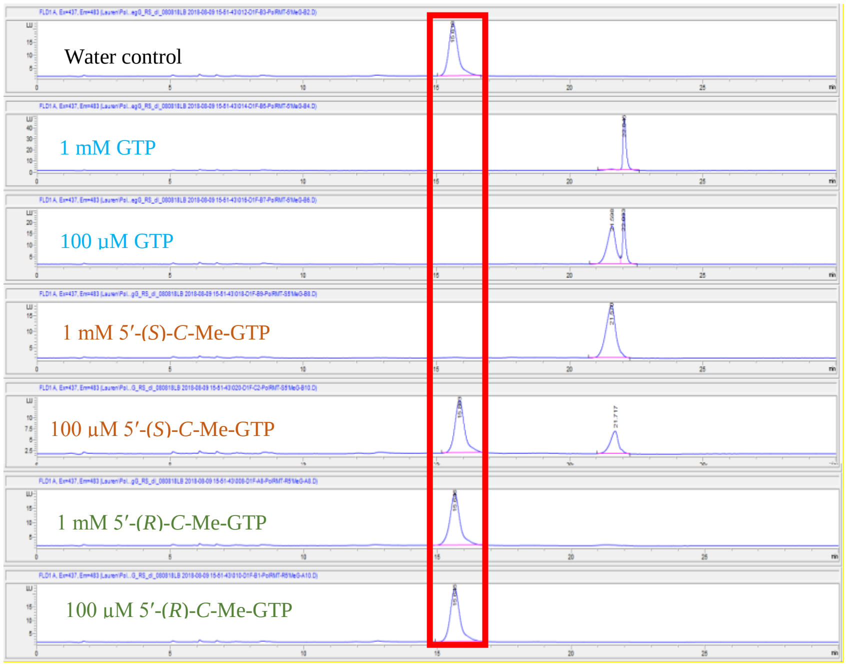

Figure S1: Incorporation of native and 5'-C-methyl-guanosine triphosphates by POLRMT *in vitro*. Reaction conditions: 200 nM template, 50 nM primer, 300 nM enzyme, and 1 mM or 100 µM NTP of interest, 30 min, 35 °C. Samples were run in duplicate and analysed using analytical HPLC using fluorescence detection. The red box marks the location of the non-extended primer.

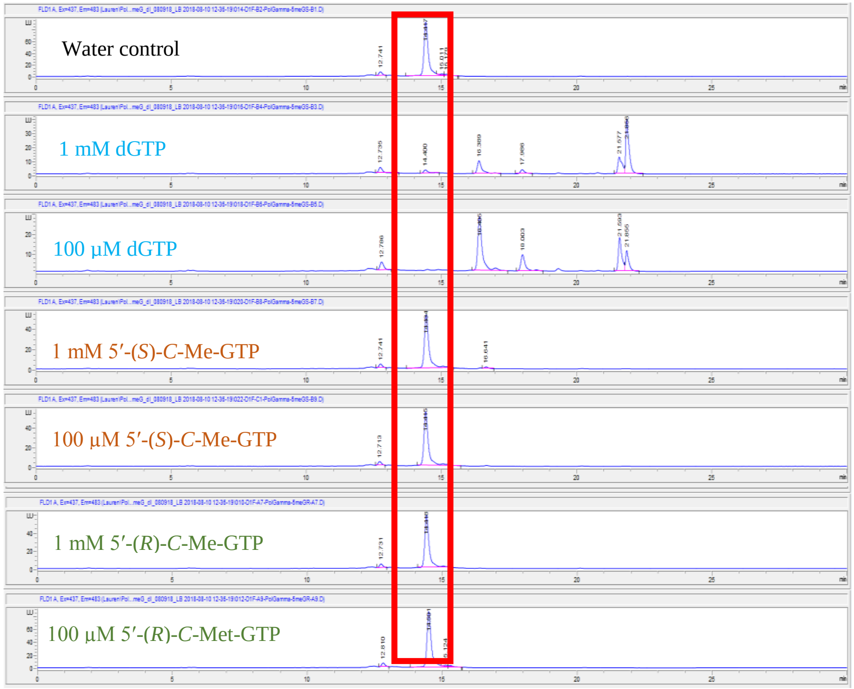

Figure S2: Incorporation of native and 5'-C-methyl guanosine triphosphates by POLG *in vitro*. Reaction conditions: 200 nM template, 50 nM primer, 300 nM enzyme, and 1 mM or 100 µM NTP of interest, 30 min, 35 °C. Samples were run in duplicate and analysed using analytical HPLC using fluorescence detection. The red box marks the location of the non-extended primer.

Supplementary Table 4 Selected crystallographic data and data collection and refinement parameters for 5′-CCCCXGGG-3′ (X=(*R*)-5′-*C*-Me-guanosine)

Space group	P61
Unit cell constants a, b, c [Å]; α, β, γ [°]	37.92, 37.92, 57.85, 90, 90, 120
Data Collection	
Wavelength [Å]	0.91833
Resolution [Å]	21.72-1.56 (1.62-1.56) [a]
No. of unique reflections	6,721 (627)
Redundancy	18.5 (12.1)
I/σ(I)	63.3 (1.33)
Completeness [%]	99.3 (92.4)
R-merge	0.063 (0.869)
R-pim	0.015 (0.243)
Refinement	
No. of reflections	6,392 (295)
R-work [%]	0.177 (0.259)
R-free [%]	0.244 (0.268)
No. of RNA atoms	340
No. of ions (Co3+)/waters	5/30
R.m.s. deviations bonds [Å]	0.029
R.m.s. deviations angles [°]	1.85
Avg. B-factor, DNA atoms [Å2]	37.6
Avg. B-factor, ions/water [Å2]	36.4/48.9
PDB entry code	6VEM

a Values in parentheses refer to the highest resolution shell

^{1}H, ^{13}C and ^{31}P NMR spectra of the new compounds

^{1}H NMR spectrum of compound **3** in DMSO-d6

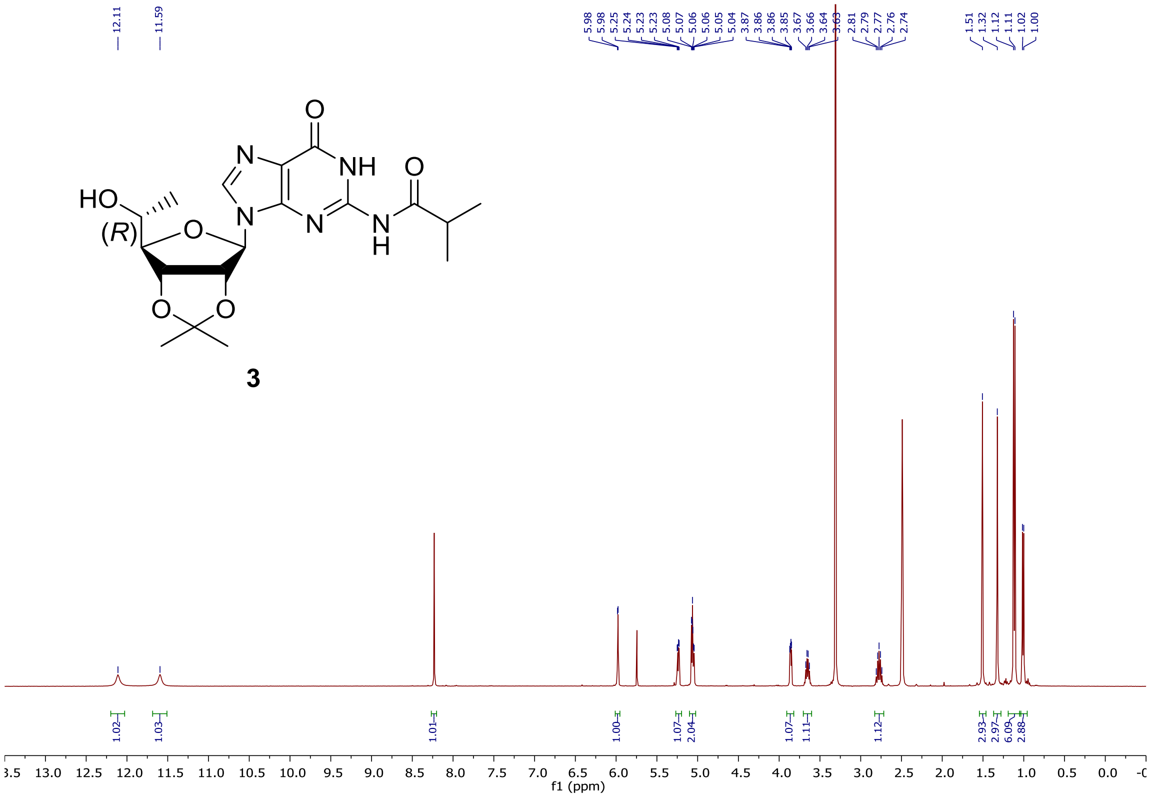

^{13}C NMR spectrum of compound **3** in DMSO-d6

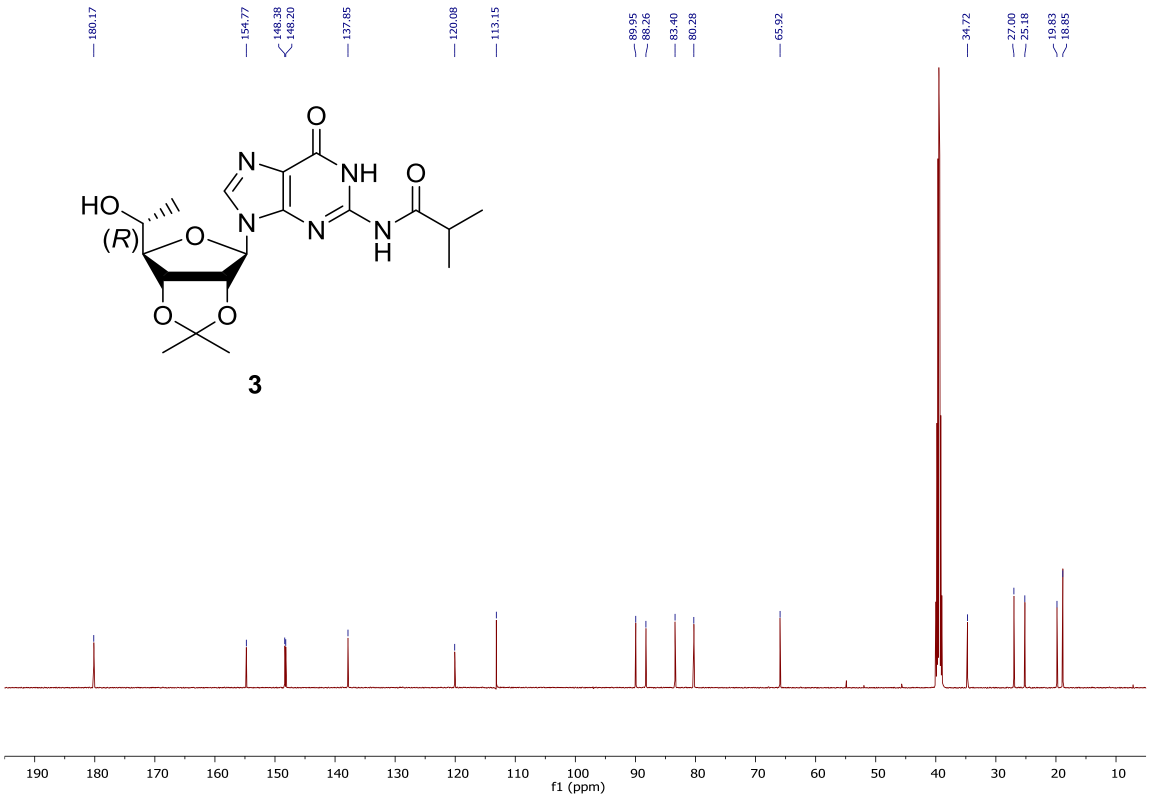

^{1}H NMR spectrum of compound **4** in DMSO-d6

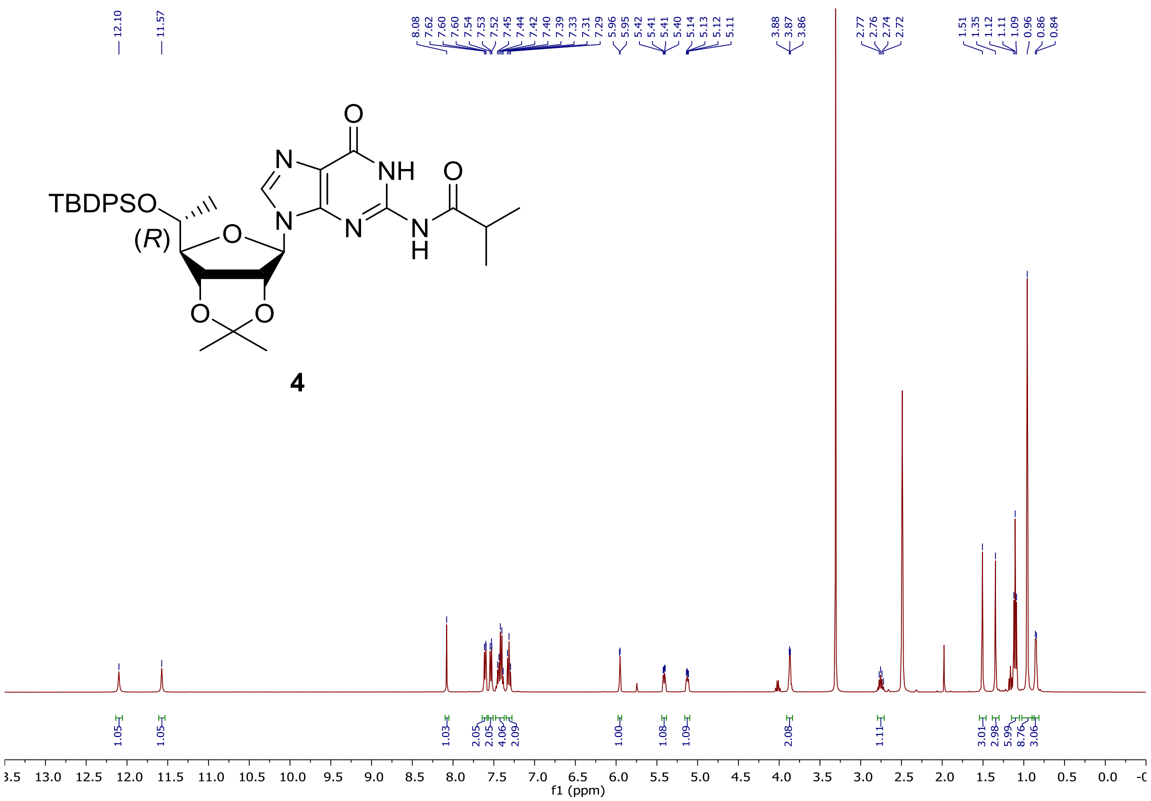

^{13}C NMR spectrum of compound **4** in DMSO-d6

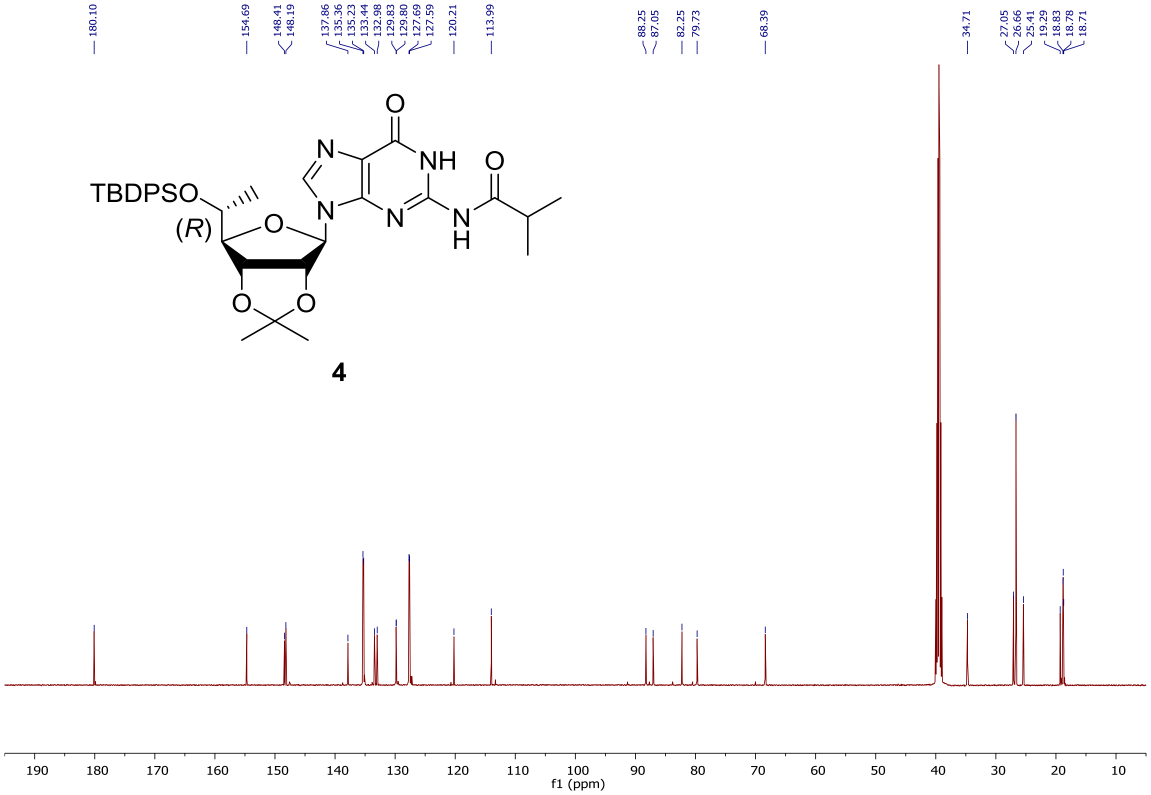

^{1}H NMR spectrum of compound **5** in DMSO-d6

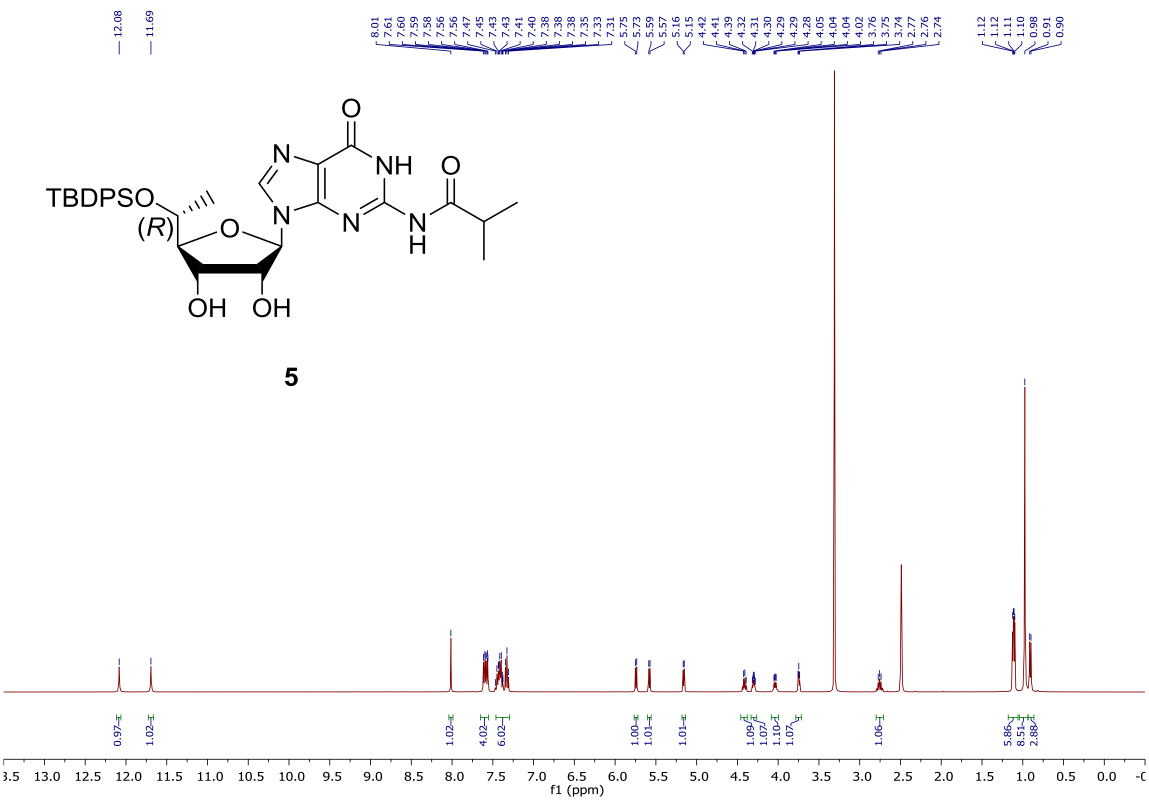

^{13}C NMR spectrum of compound **5** in DMSO-d6

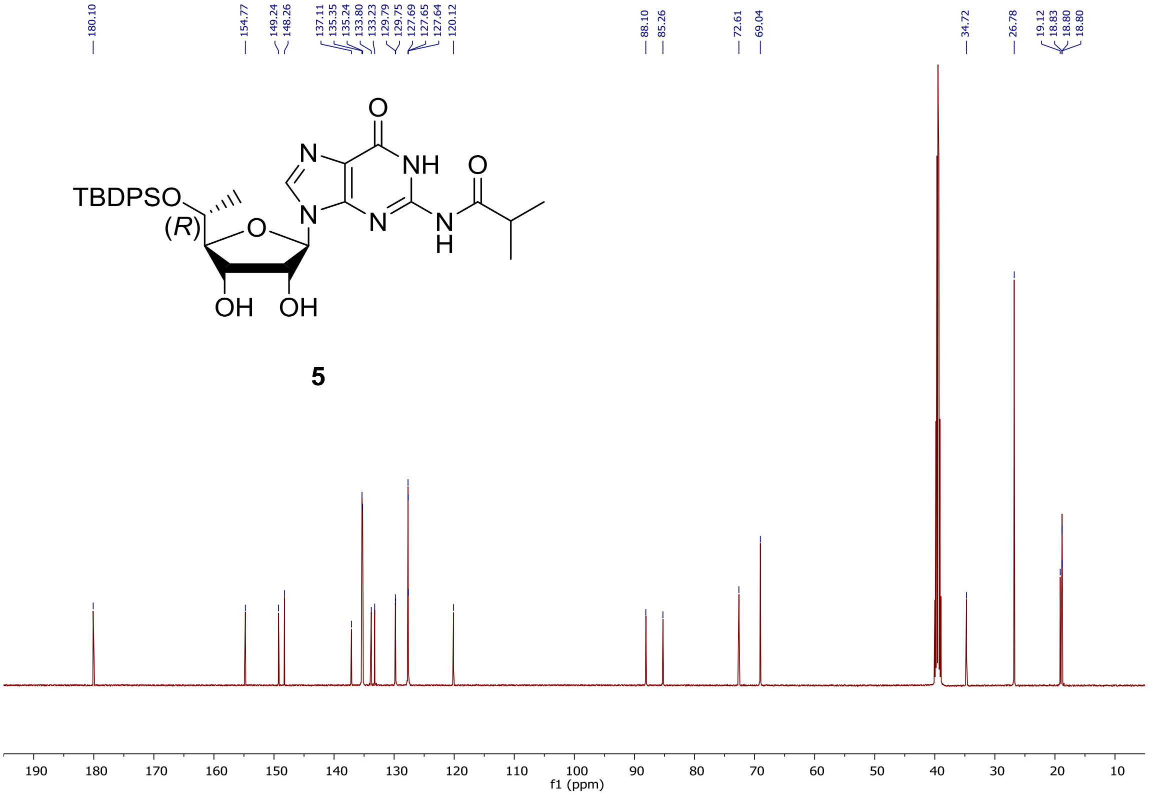

^{1}H NMR spectrum of compound **6** in DMSO-d6

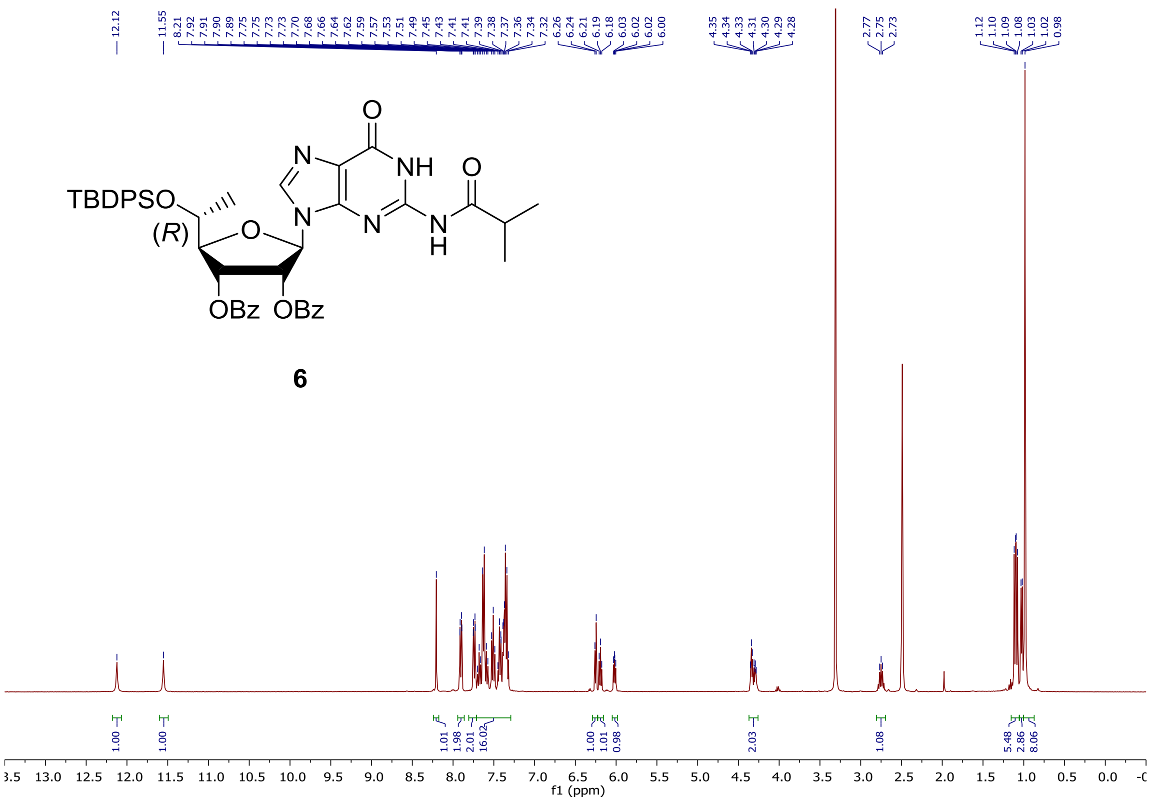

^{13}C NMR spectrum of compound **6** in DMSO-d6

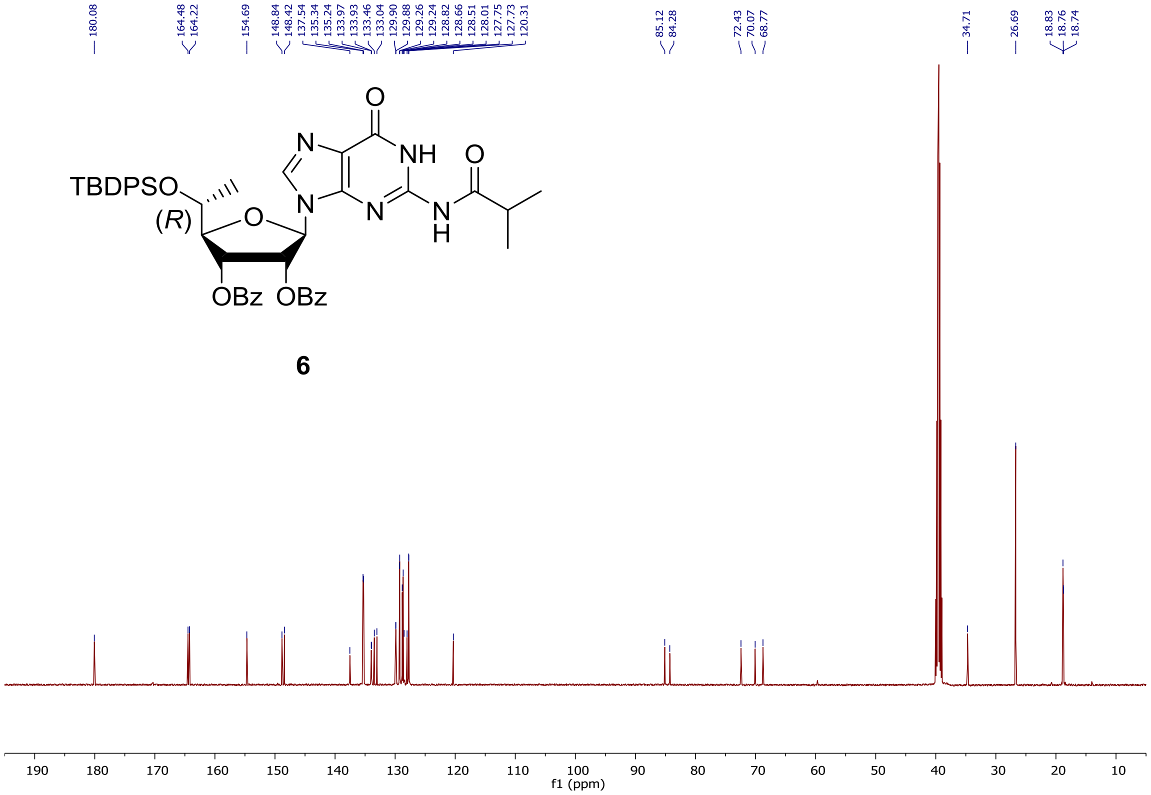

^{1}H NMR spectrum of compound **7** in DMSO-d6

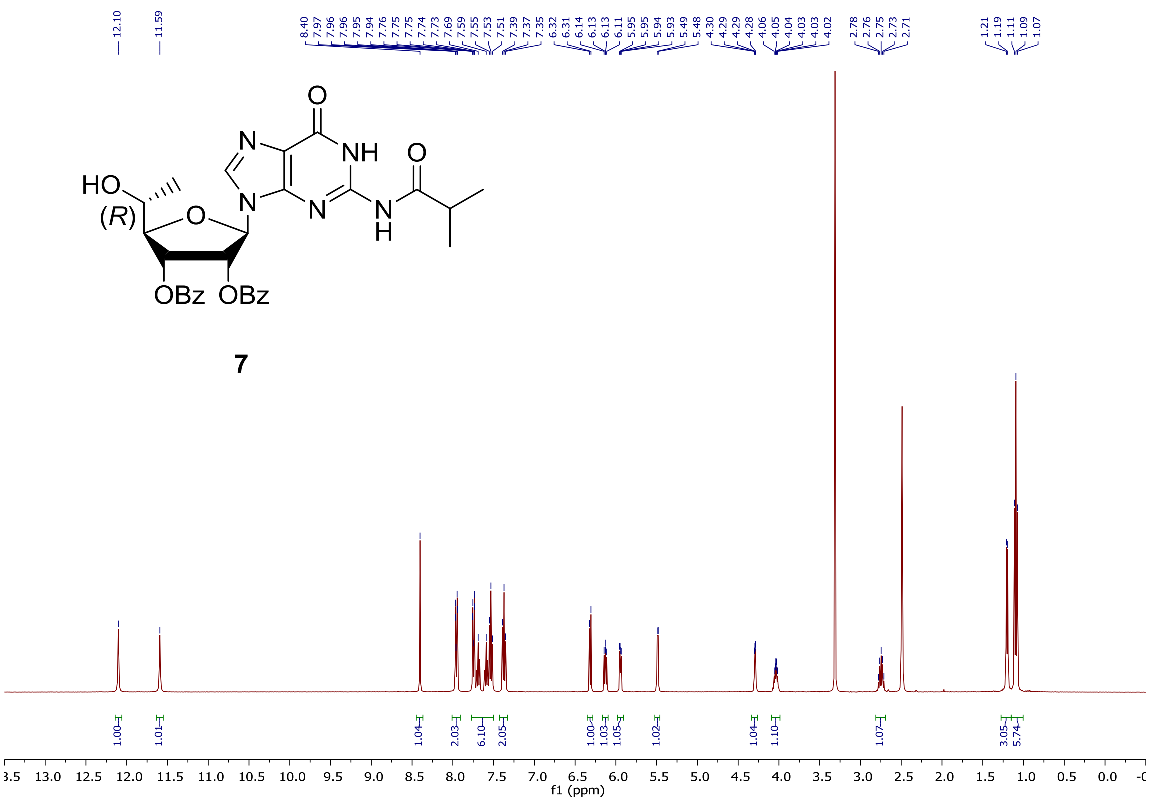

^{13}C NMR spectrum of compound **7** in DMSO-d6

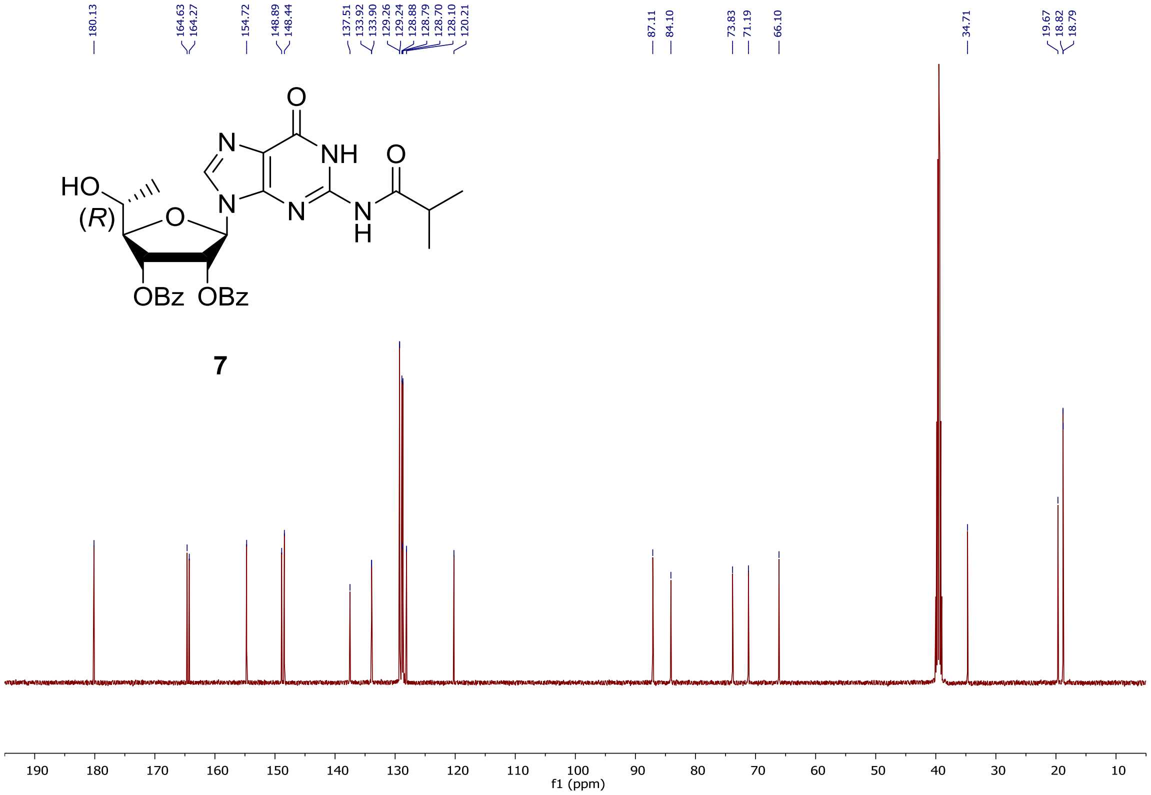

^{1}H NMR spectrum of compound **8** in DMSO-d6

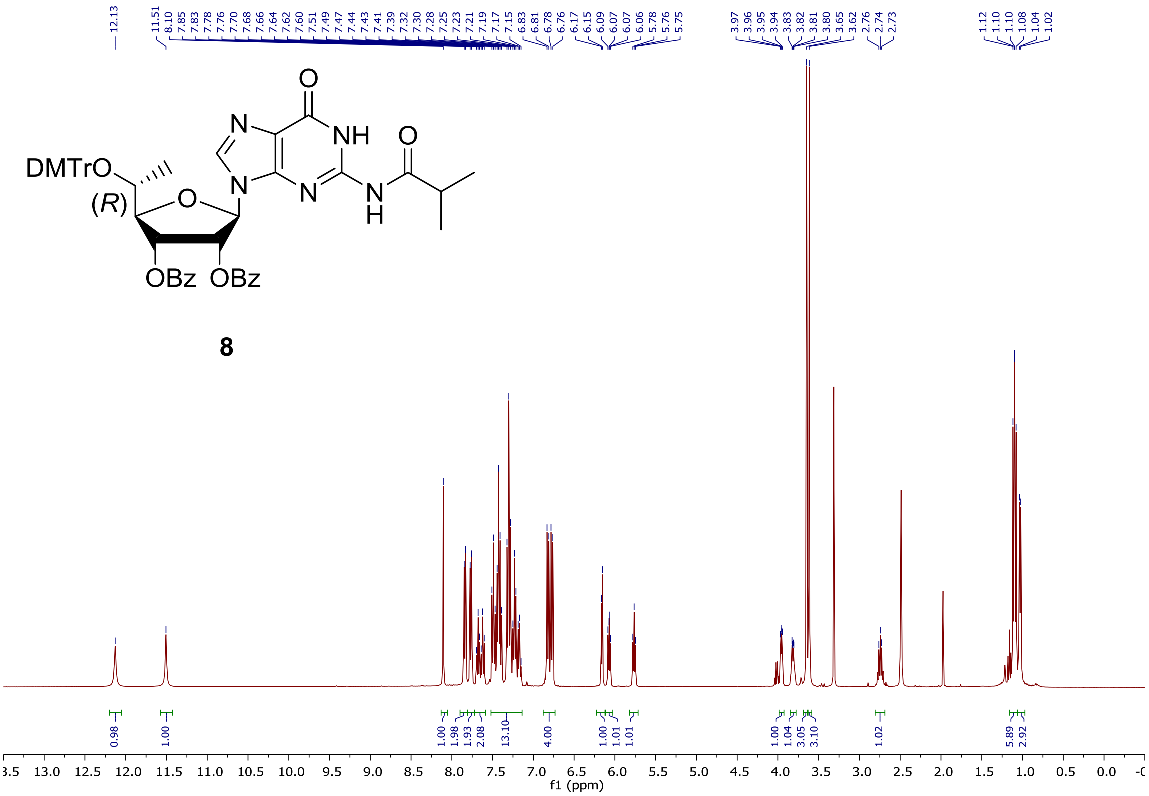

^{13}C NMR spectrum of compound **8** in DMSO-d6

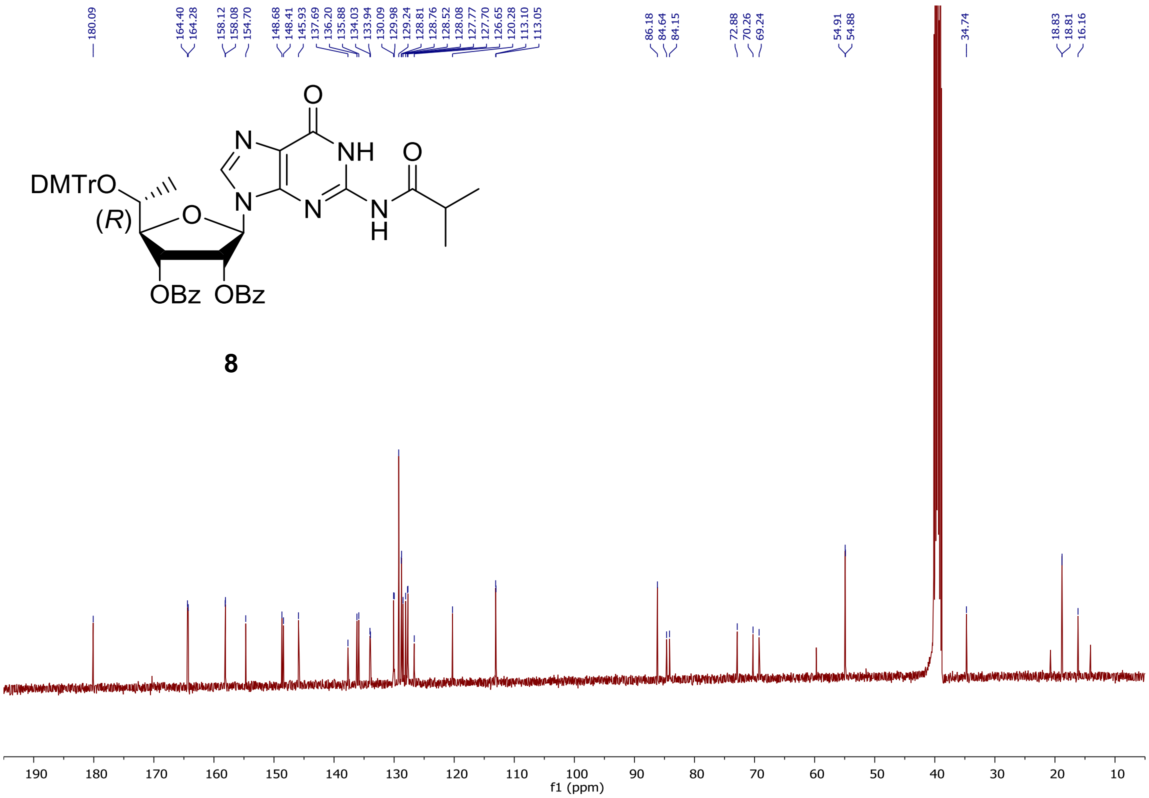

^{1}H NMR spectrum of compound **9** in DMSO-d6

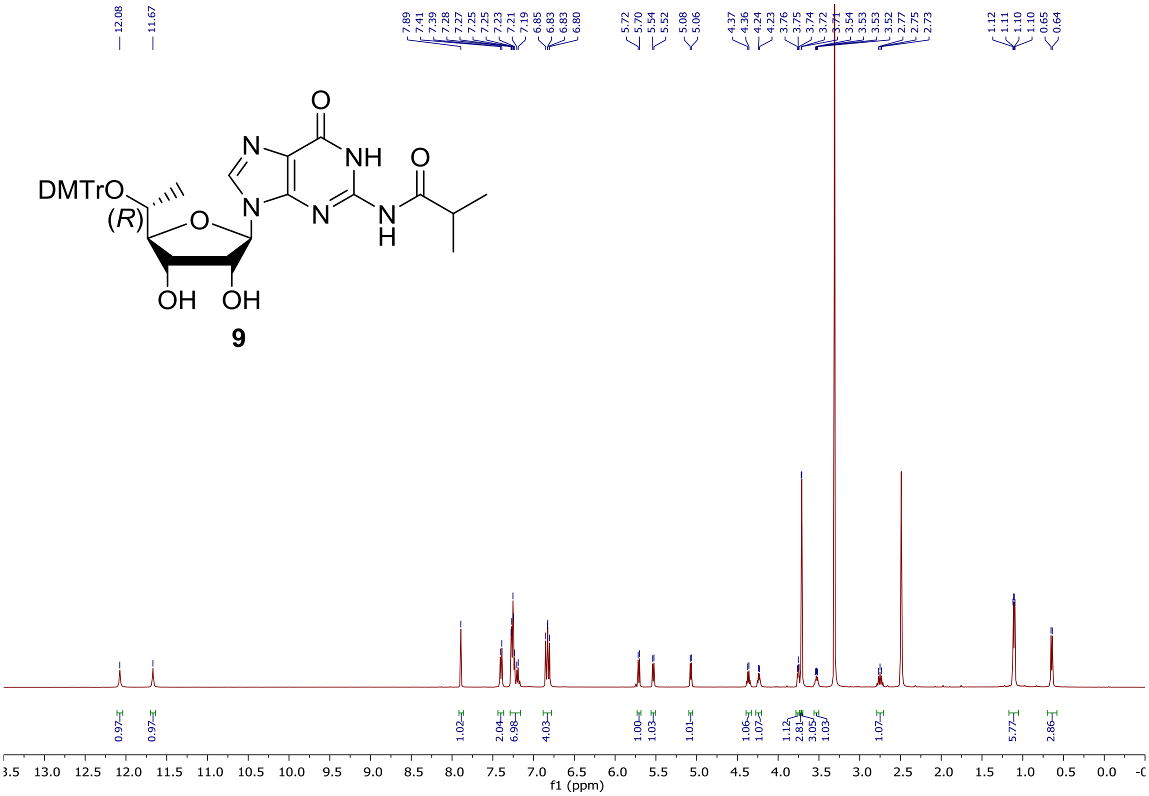

^{13}C NMR spectrum of compound **9** in DMSO-d6

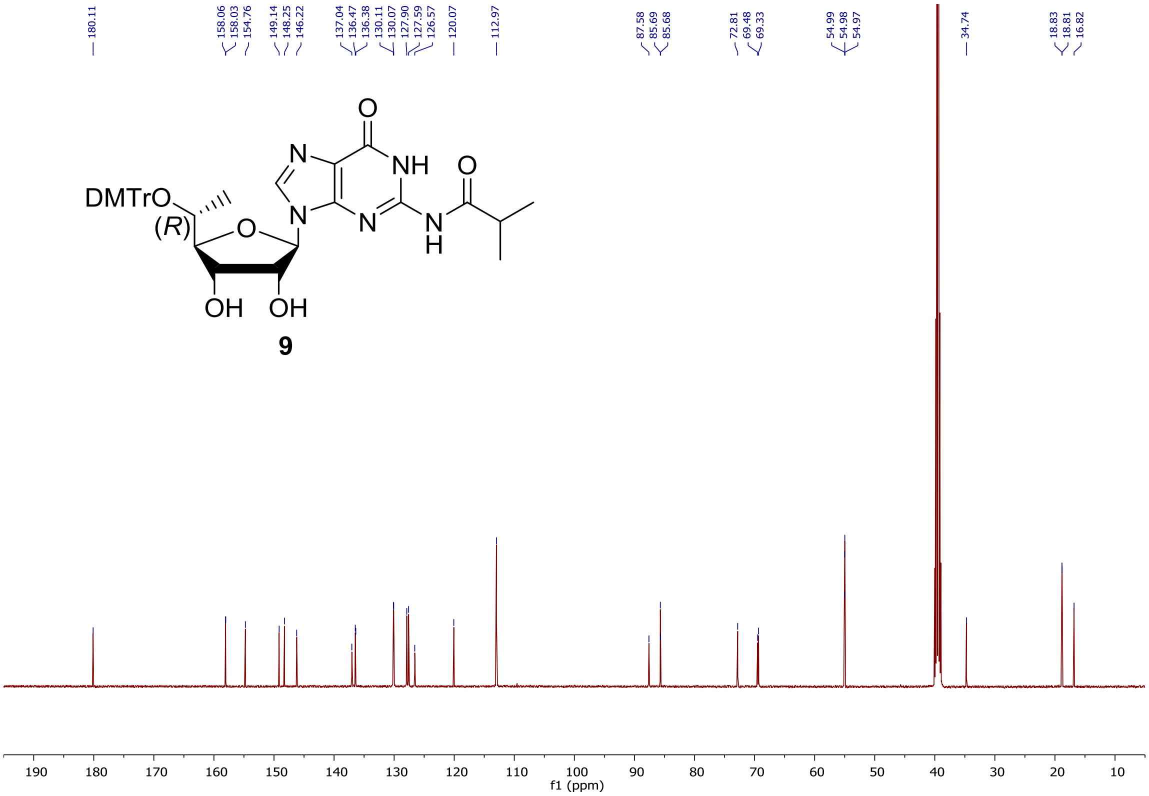

^{1}H NMR spectrum of compound 10 in DMSO-d6

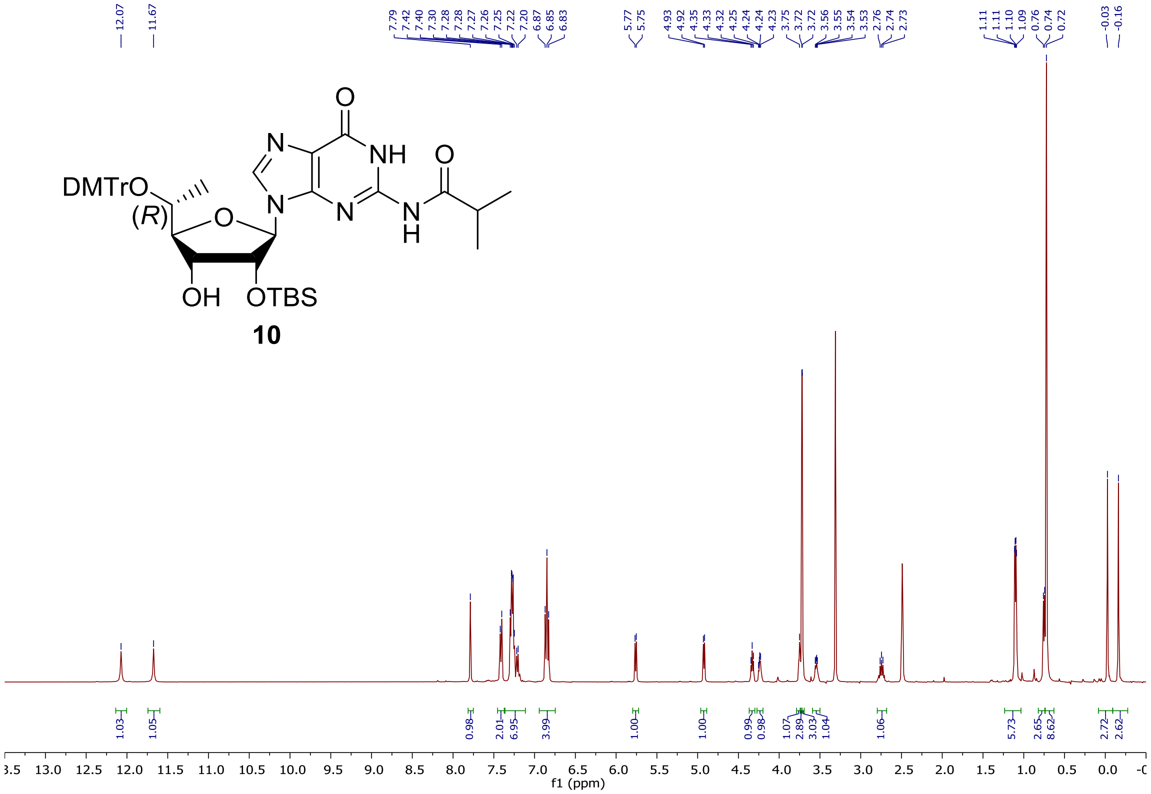

^{13}C NMR spectrum of compound 10 in DMSO-d6

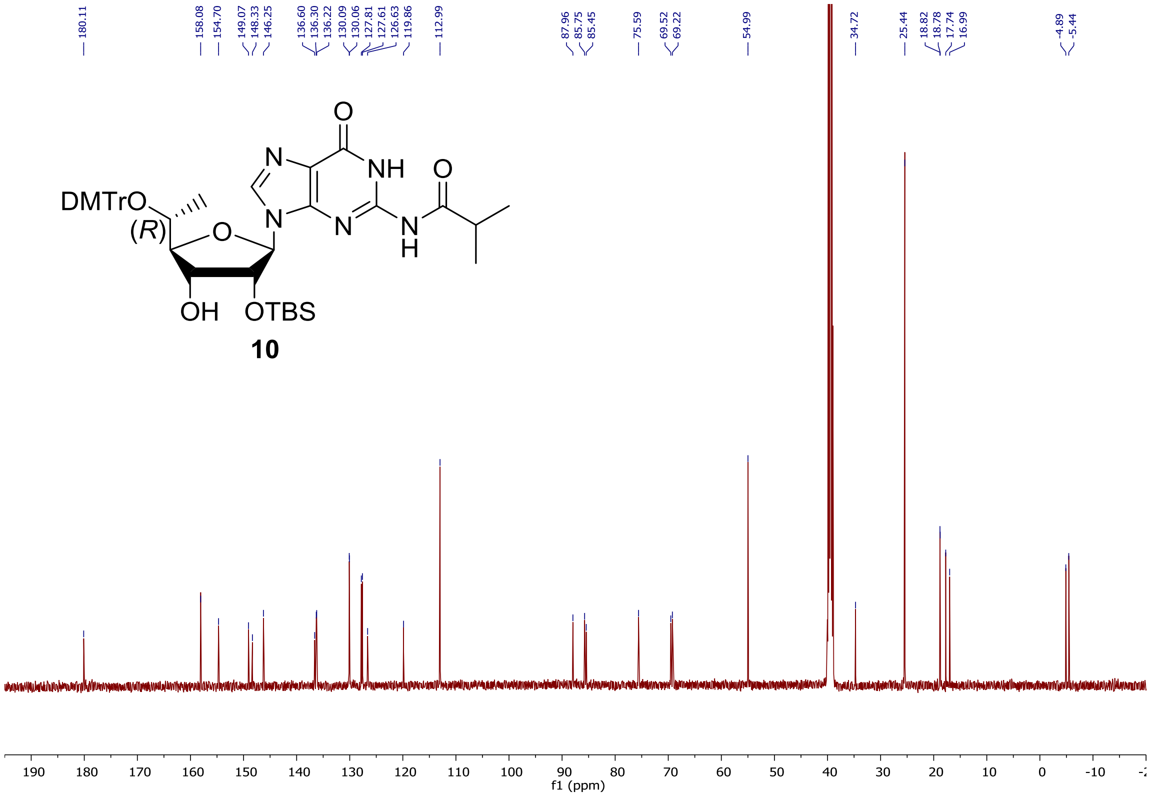

^{1}H NMR spectrum of compound 11 in $CDCl_3$

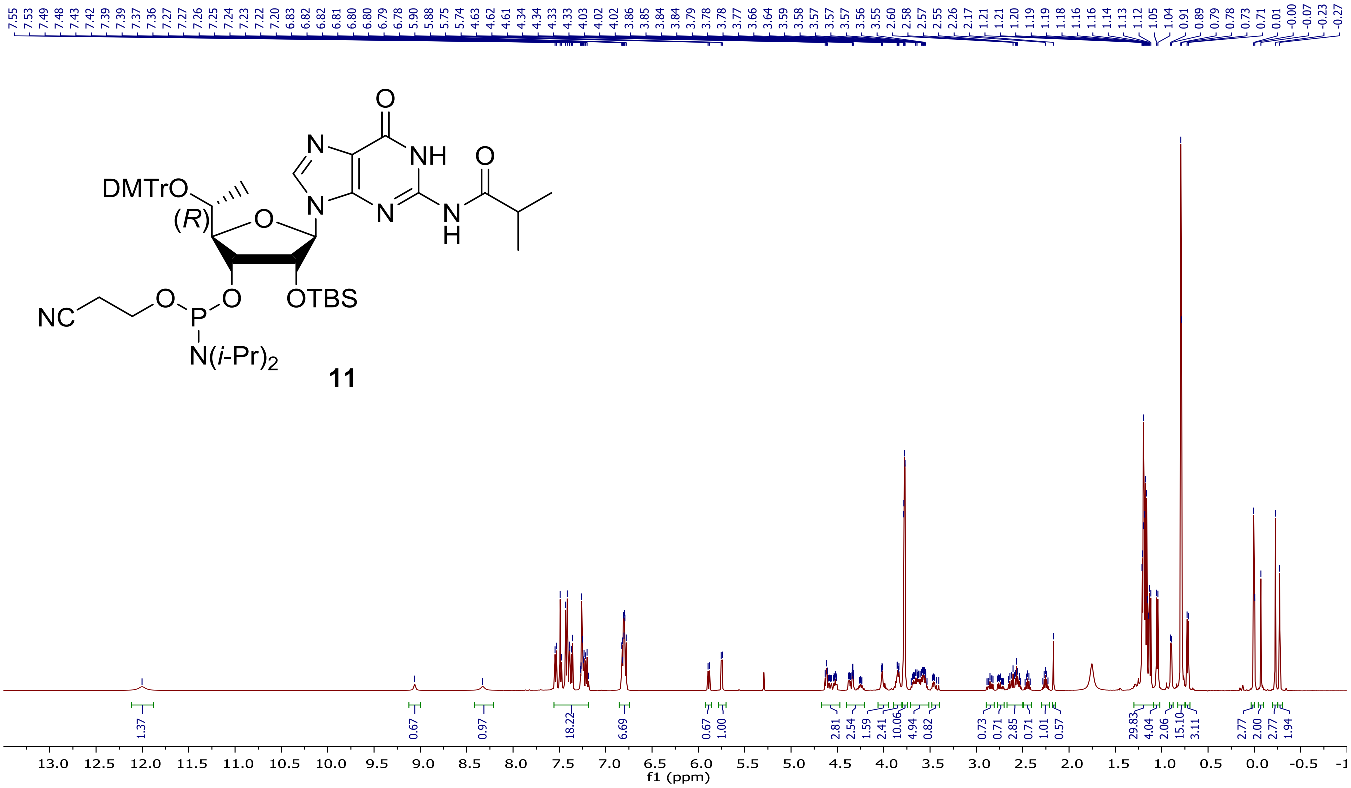

^{31}P NMR spectrum of compound 11 in $CDCl_3$

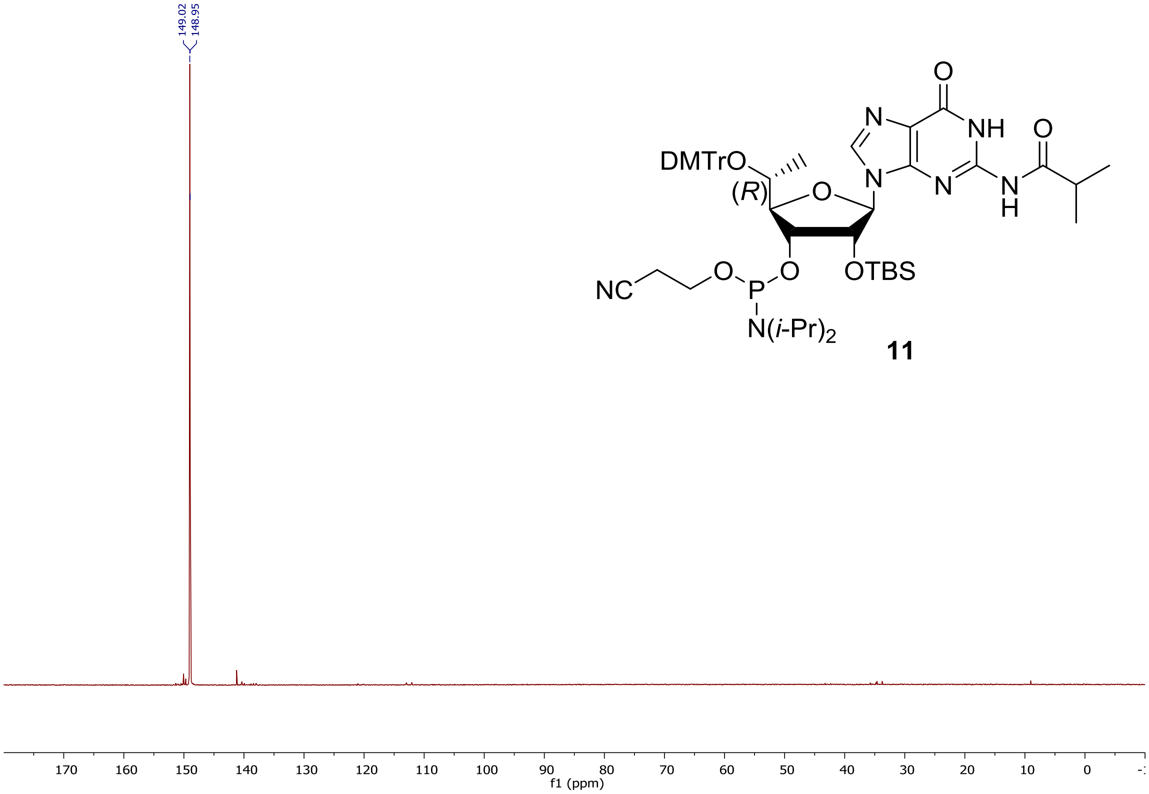

^{13}C NMR spectrum of compound 11 in $CDCl_3$

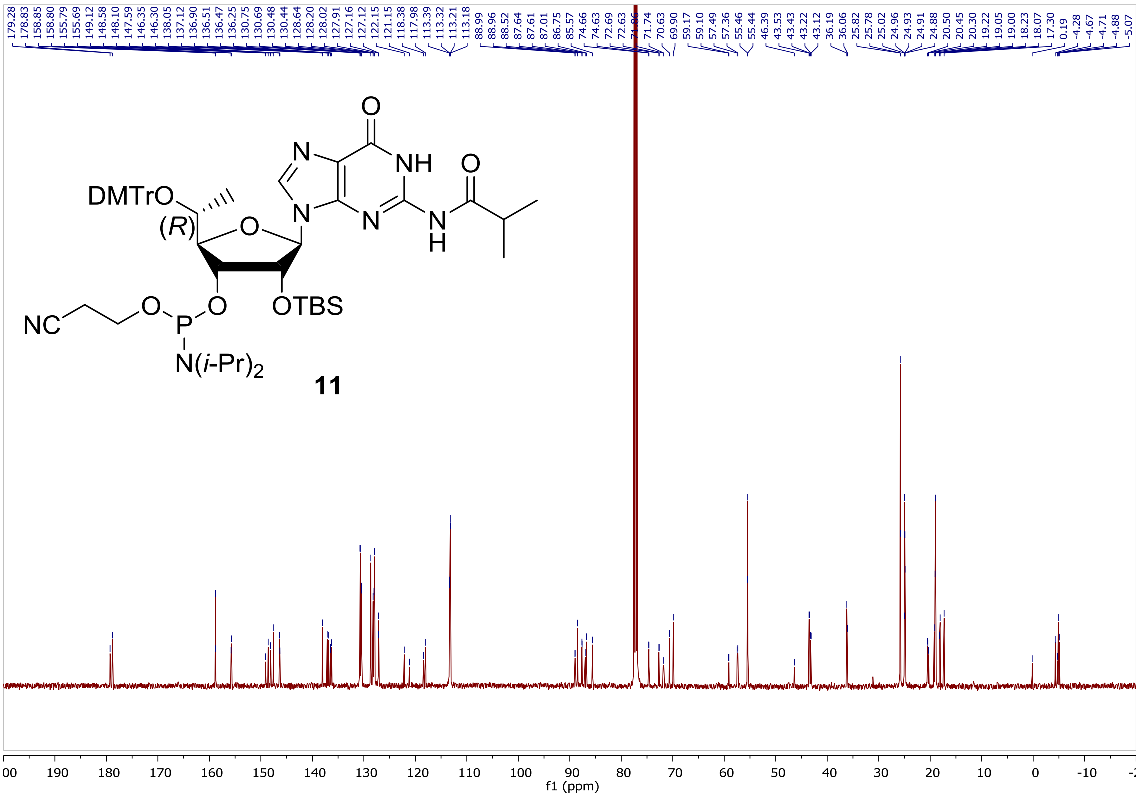

^{1}H NMR spectrum of compound 13 in DMSO-d6

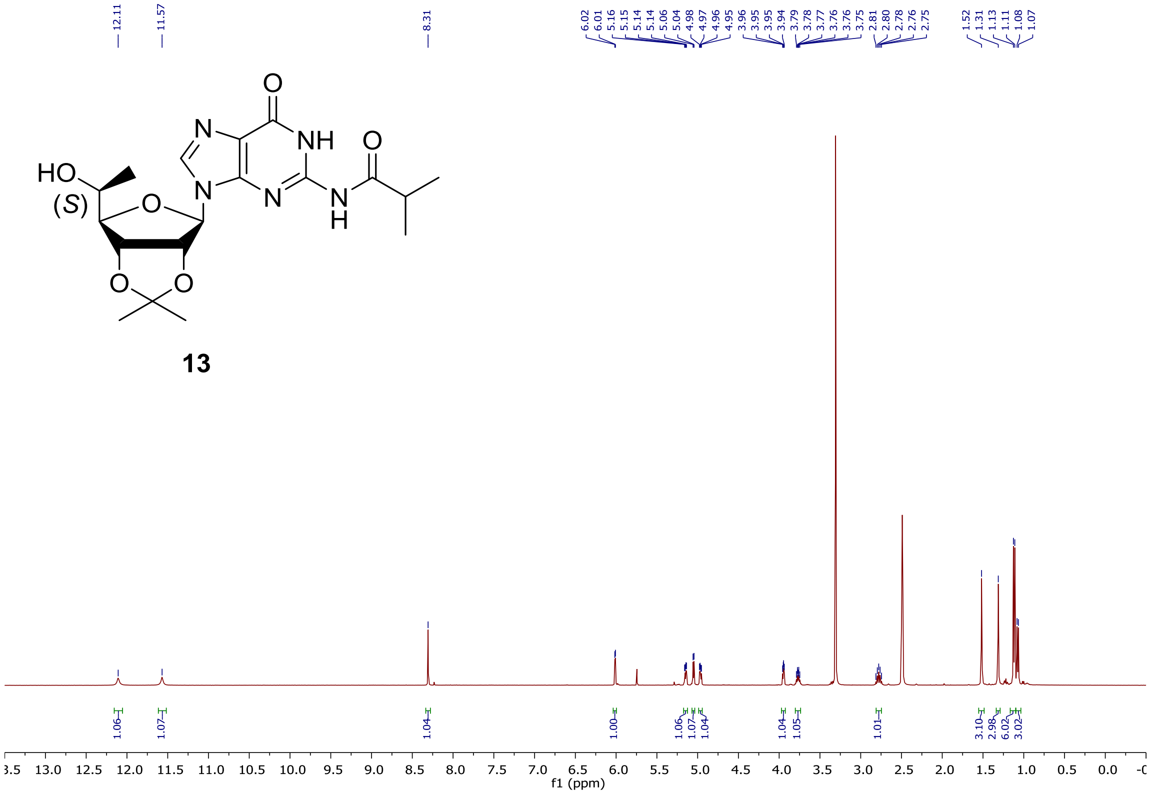

^{13}C NMR spectrum of compound 13 in DMSO-d6

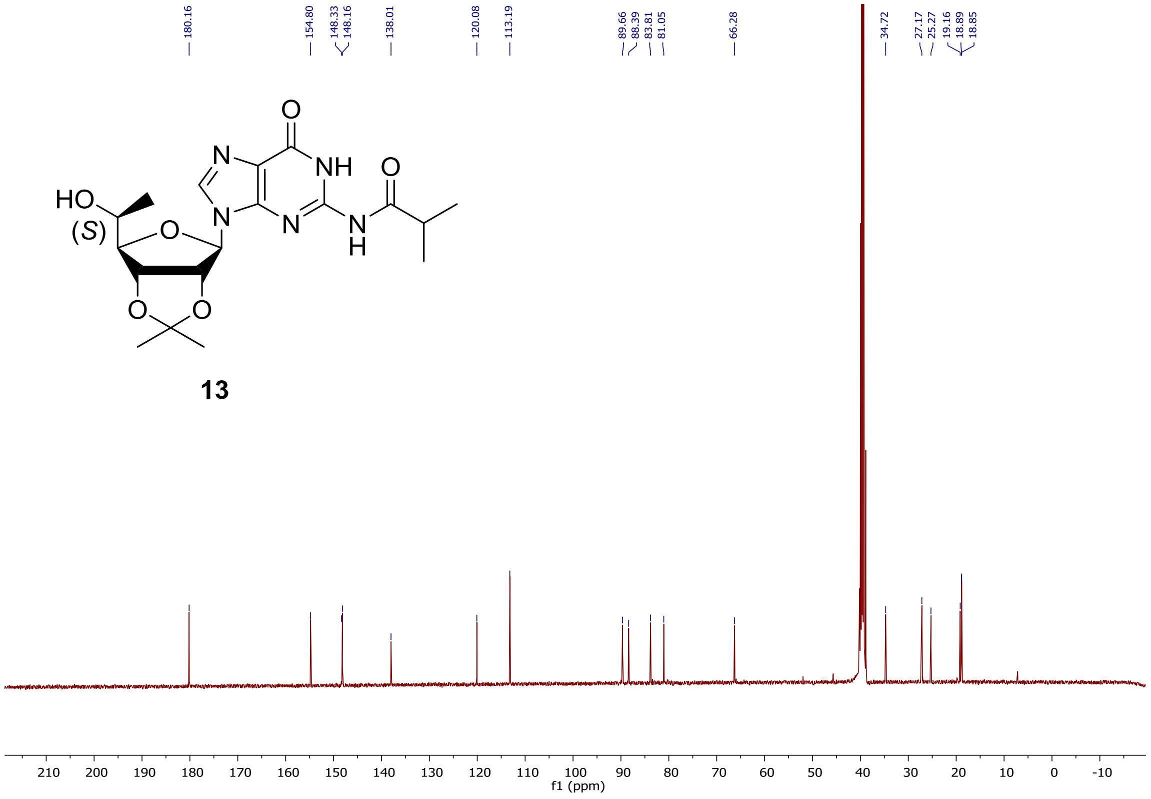

^{1}H NMR spectrum of compound 14 in DMSO-d6

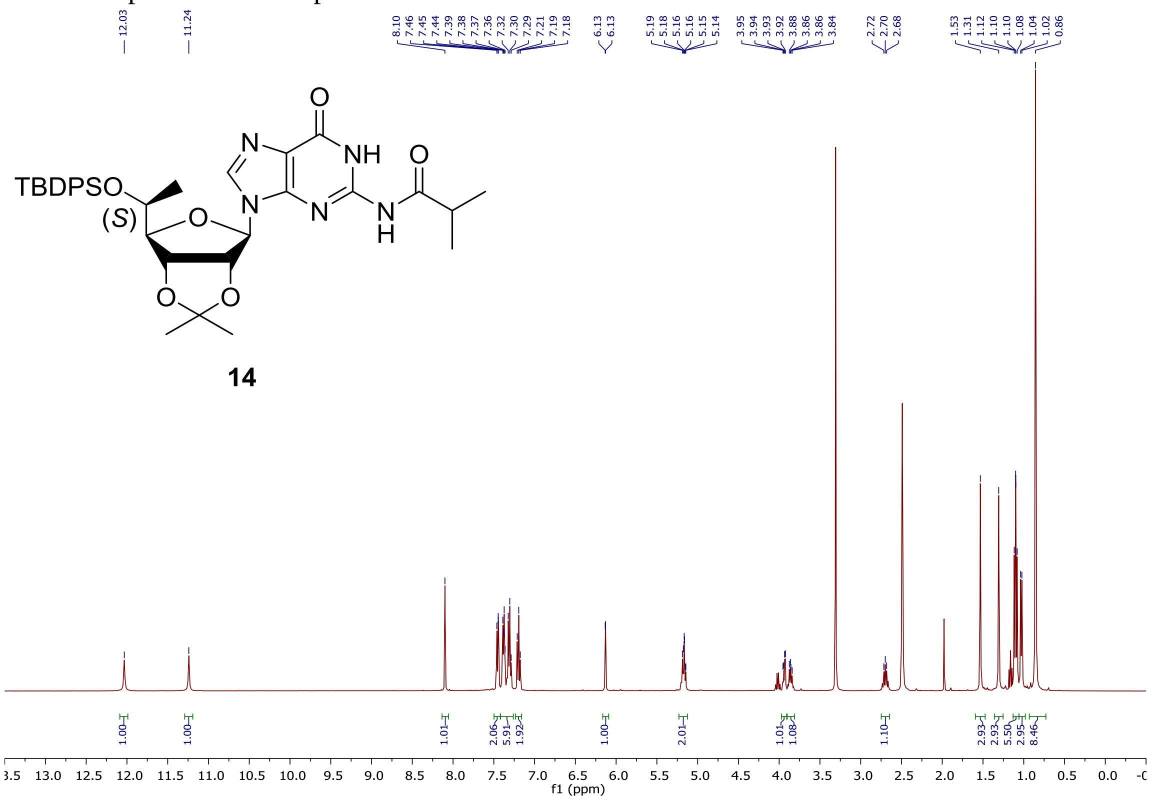

^{13}C NMR spectrum of compound 14 in DMSO-d6

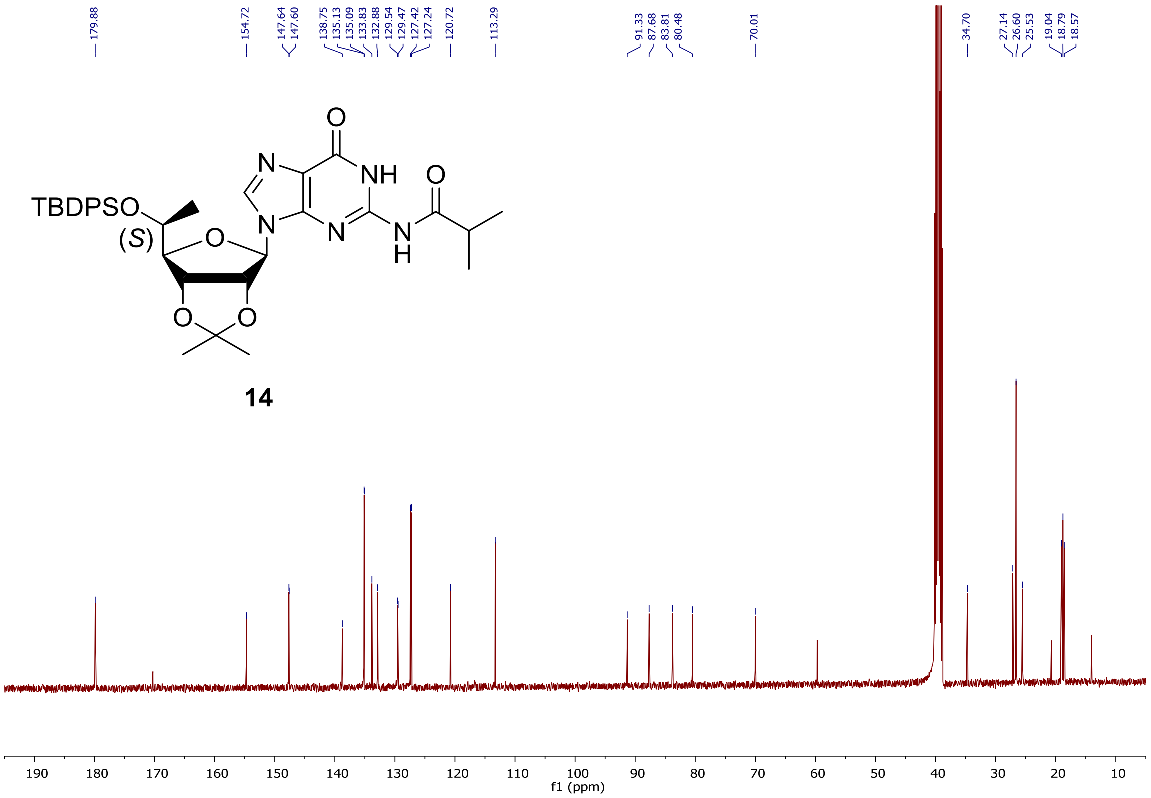

^{1}H NMR spectrum of compound 15 in DMSO-d6

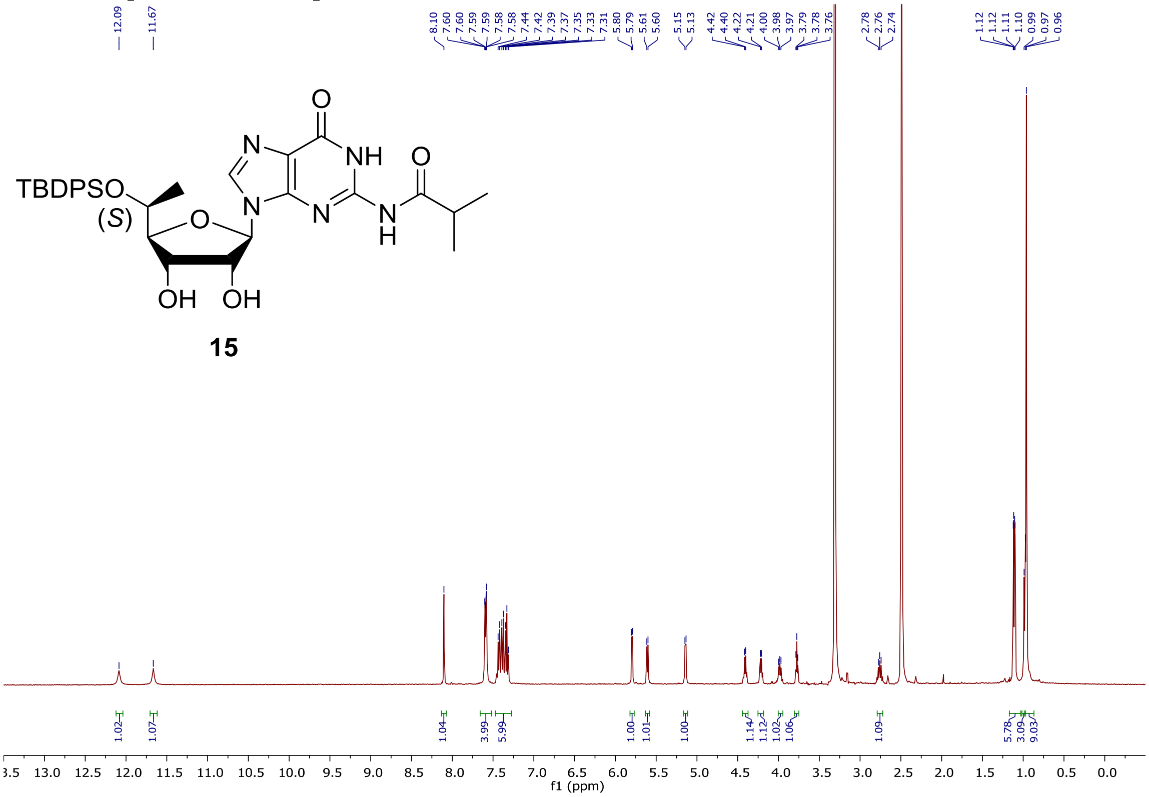

^{13}C NMR spectrum of compound 15 in DMSO-d6

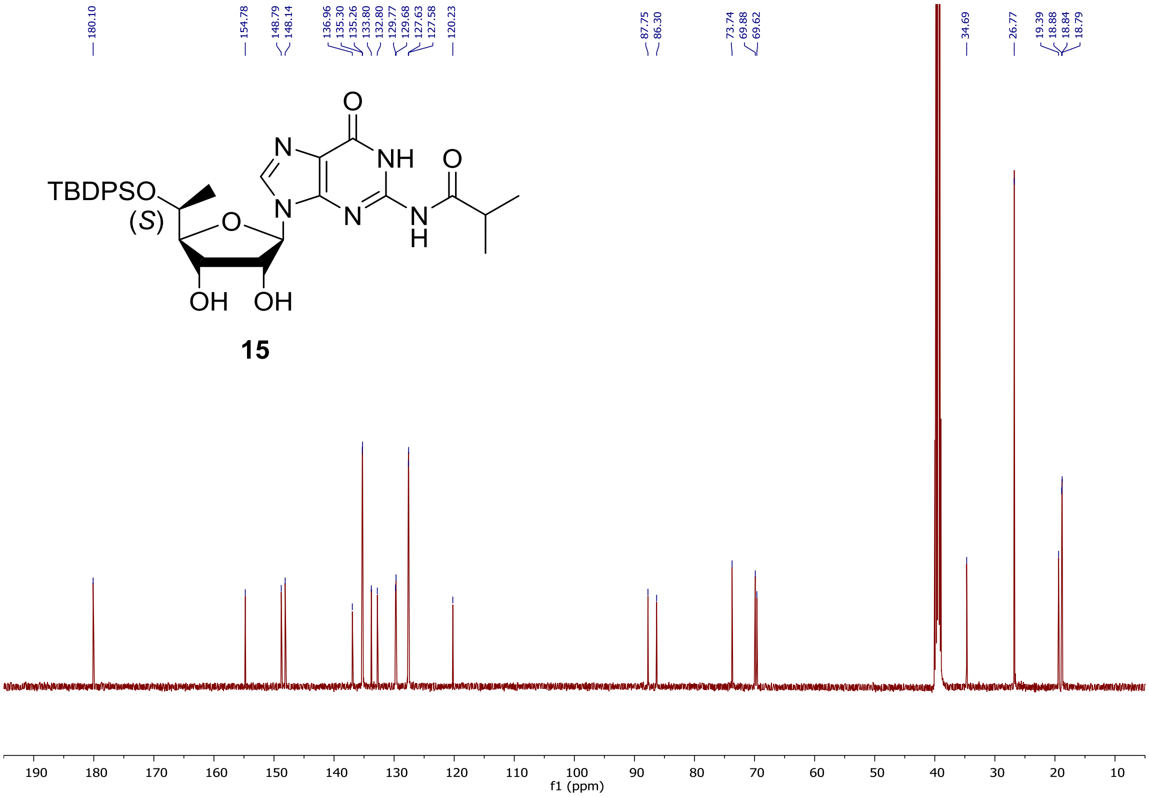

^{1}H NMR spectrum of compound 16 in DMSO-d6

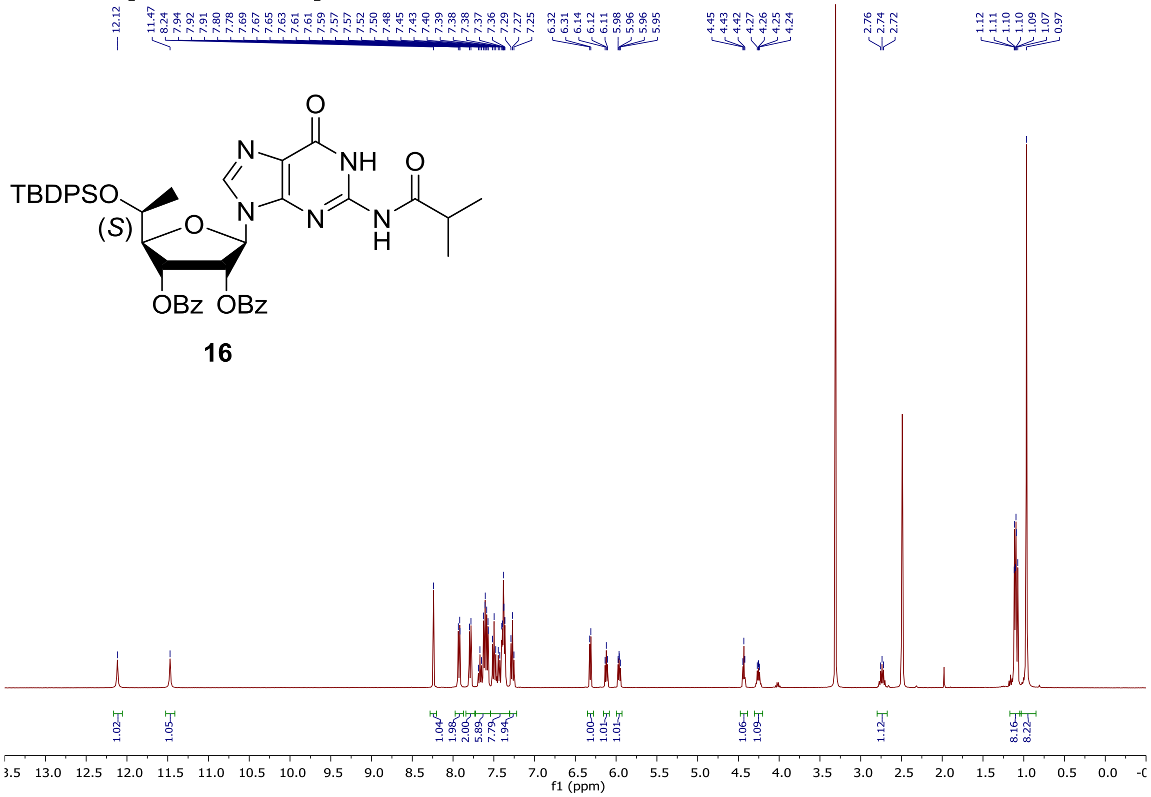

^{13}C NMR spectrum of compound 16 in DMSO-d6

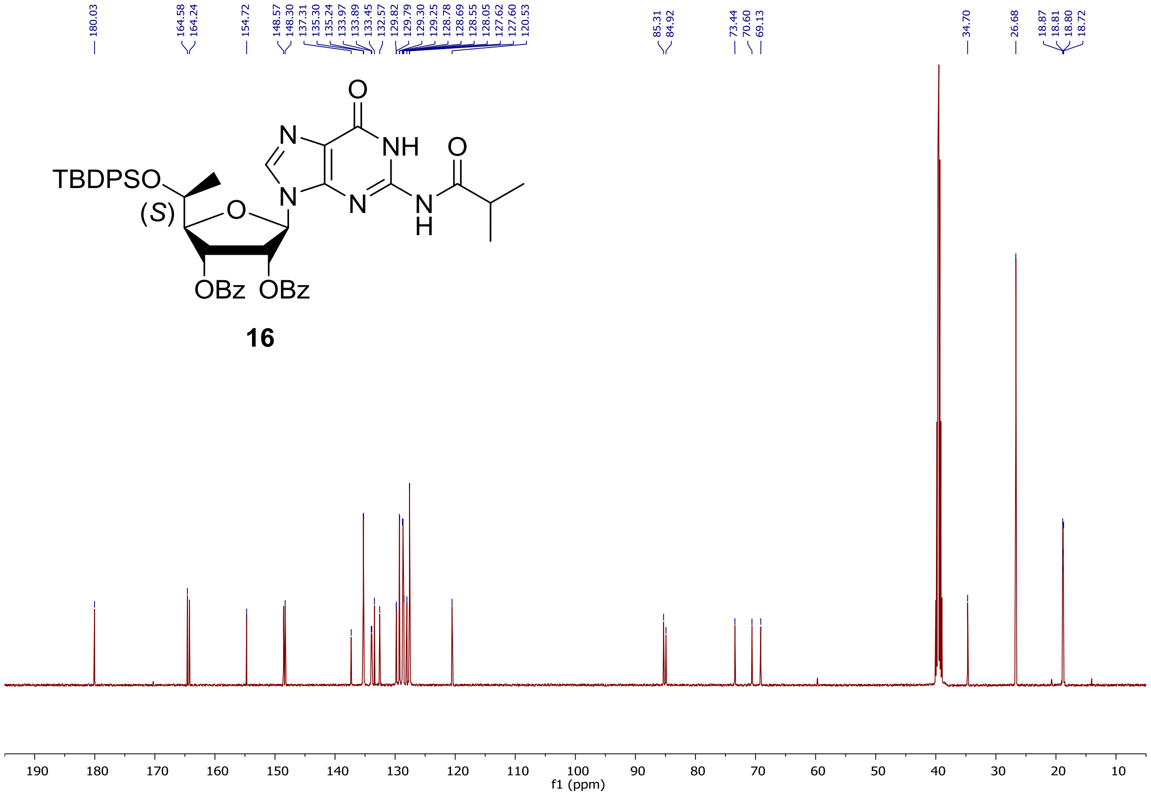

^{1}H NMR spectrum of compound 17 in DMSO-d6

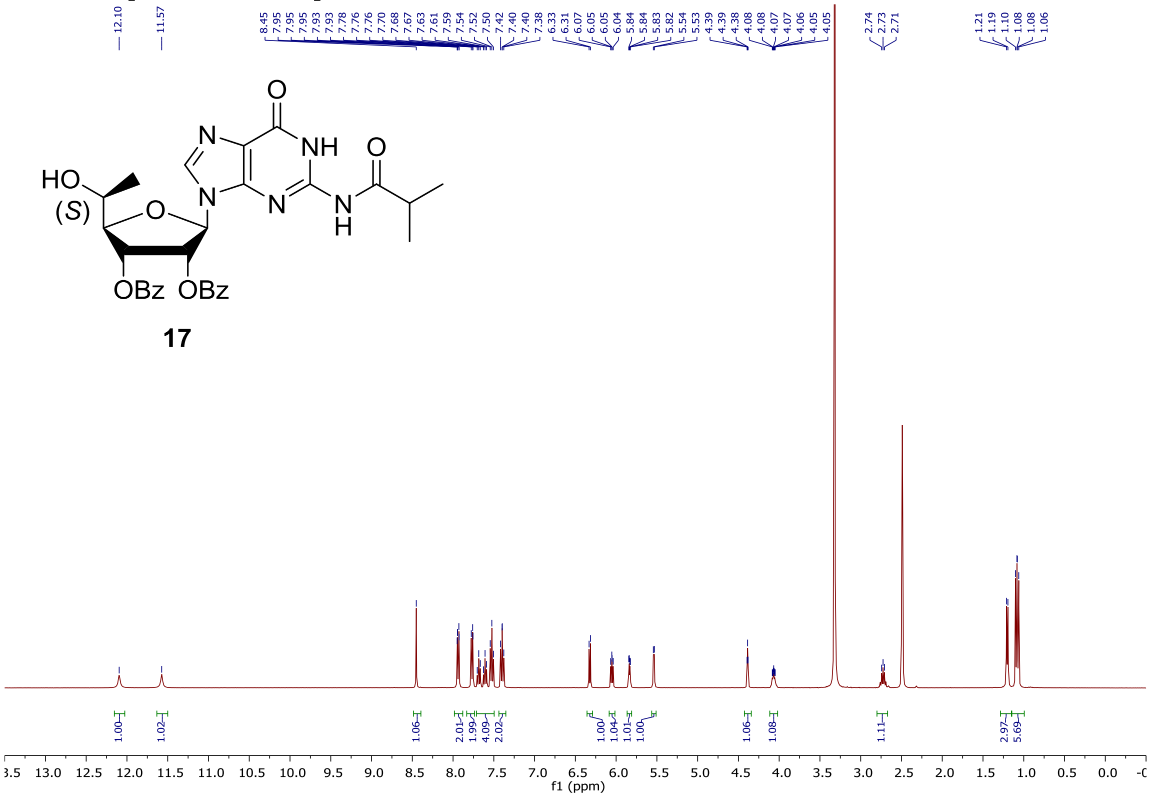

^{13}C NMR spectrum of compound 17 in DMSO-d6

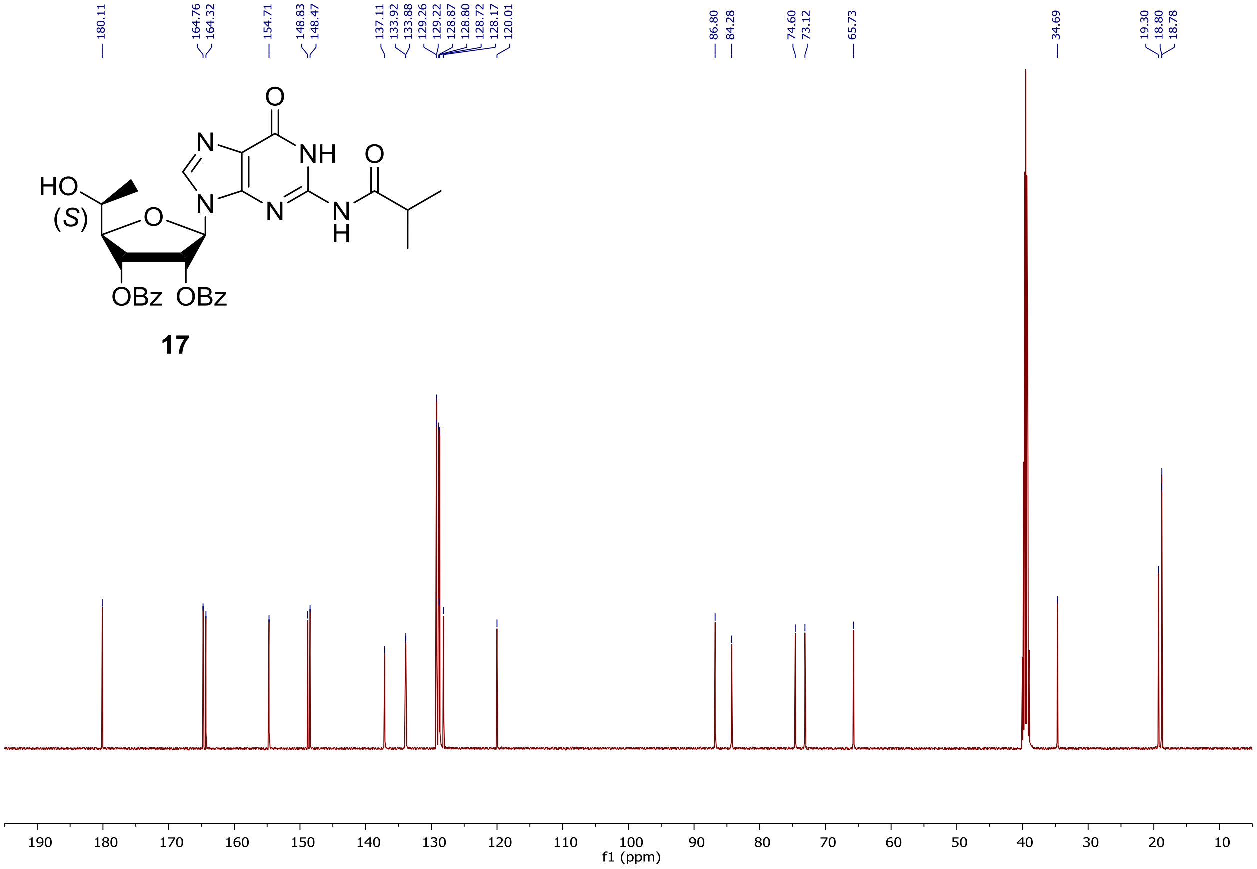

^{1}H NMR spectrum of compound 18 in DMSO-d6

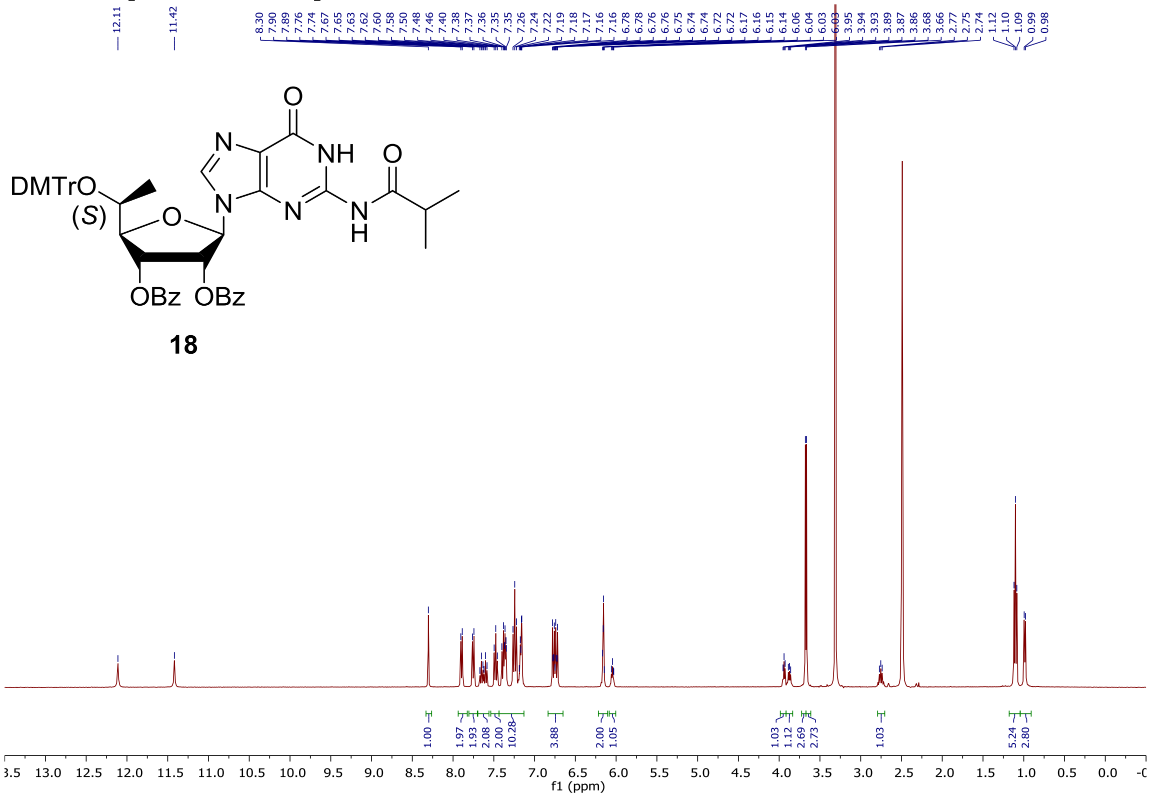

^{13}C NMR spectrum of compound 18 in DMSO-d6

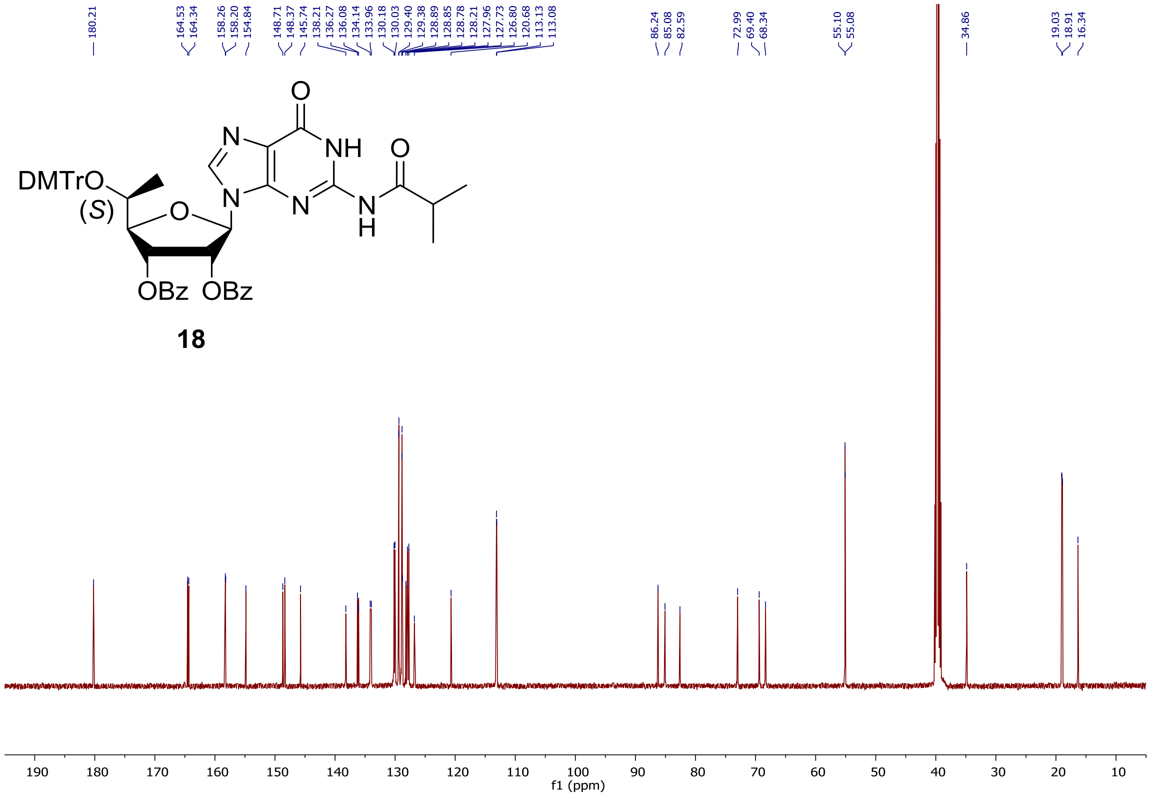

^{1}H NMR spectrum of compound 19 in DMSO-d6

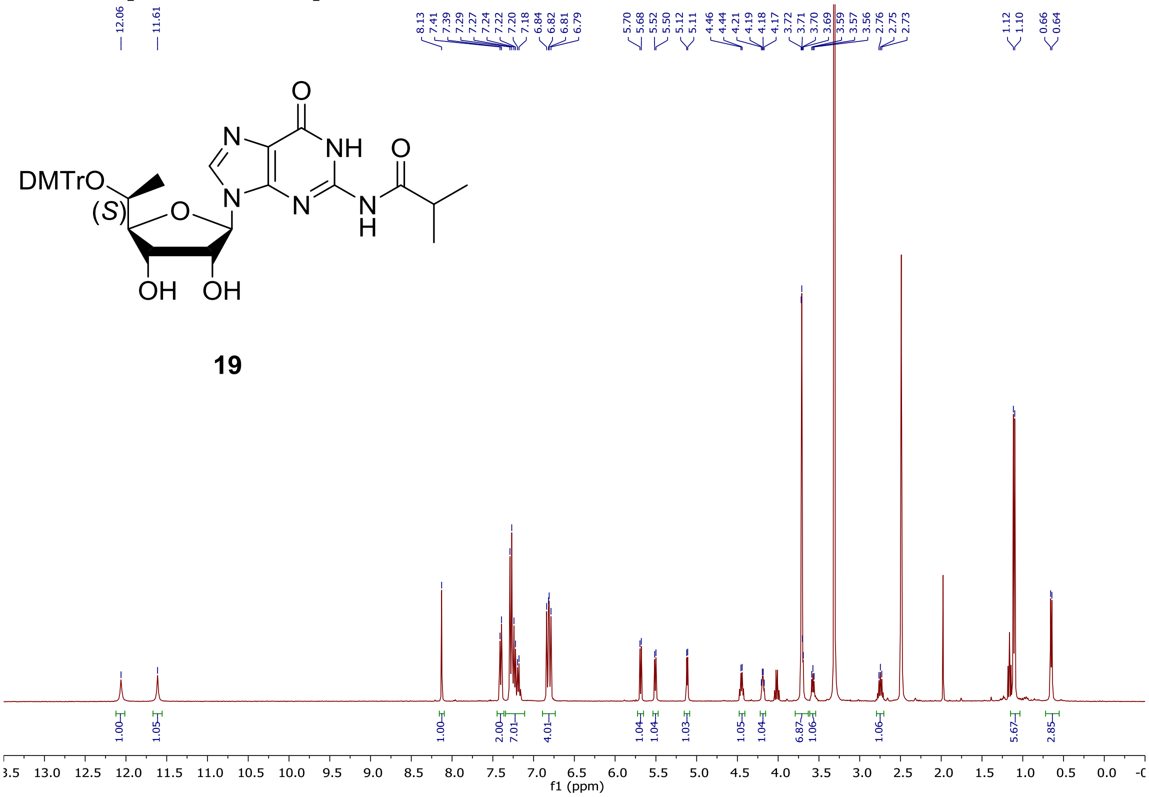

^{13}C NMR spectrum of compound 19 in DMSO-d6

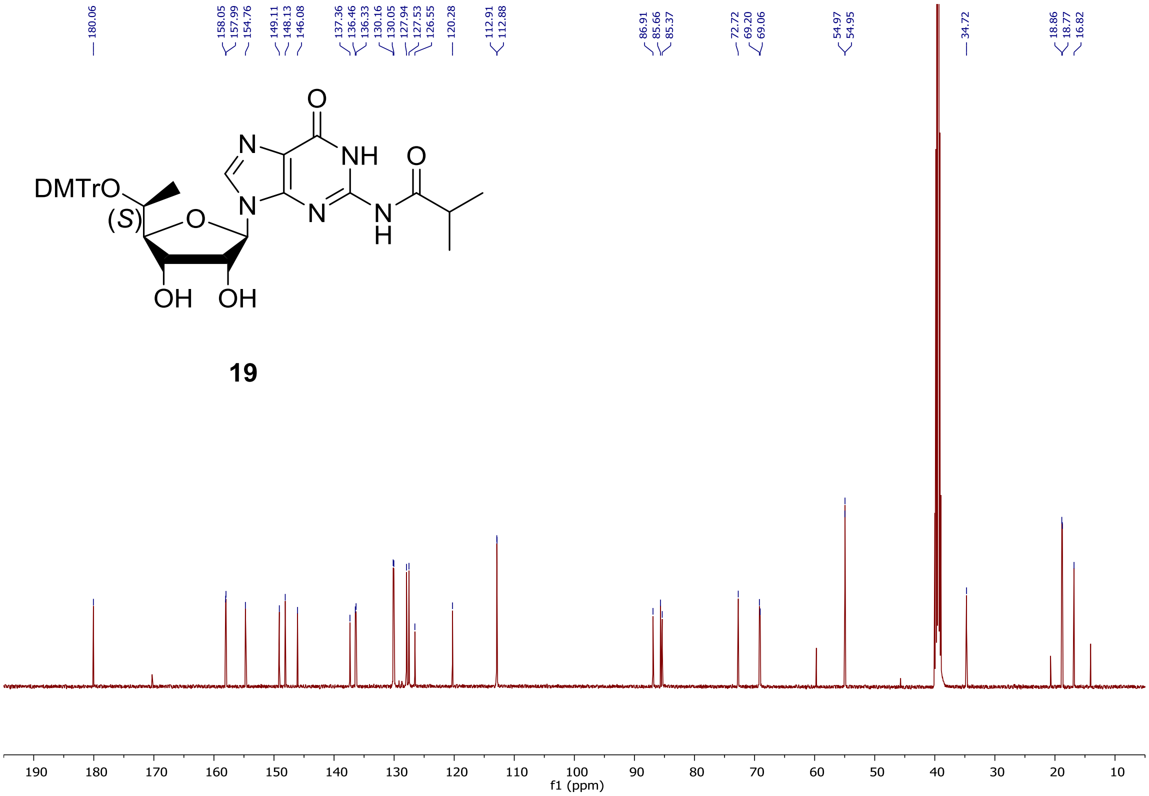

^{1}H NMR spectrum of compound 20 in DMSO-d6

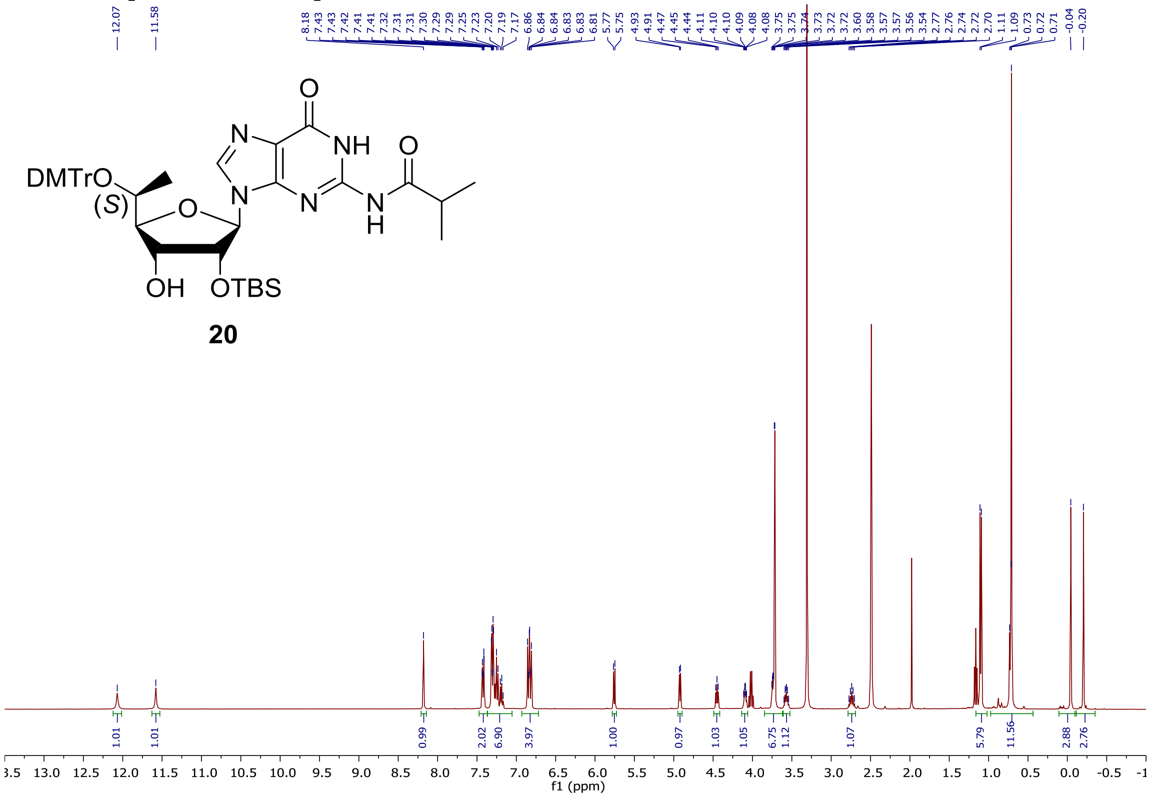

^{13}C NMR spectrum of compound 20 in DMSO-d6

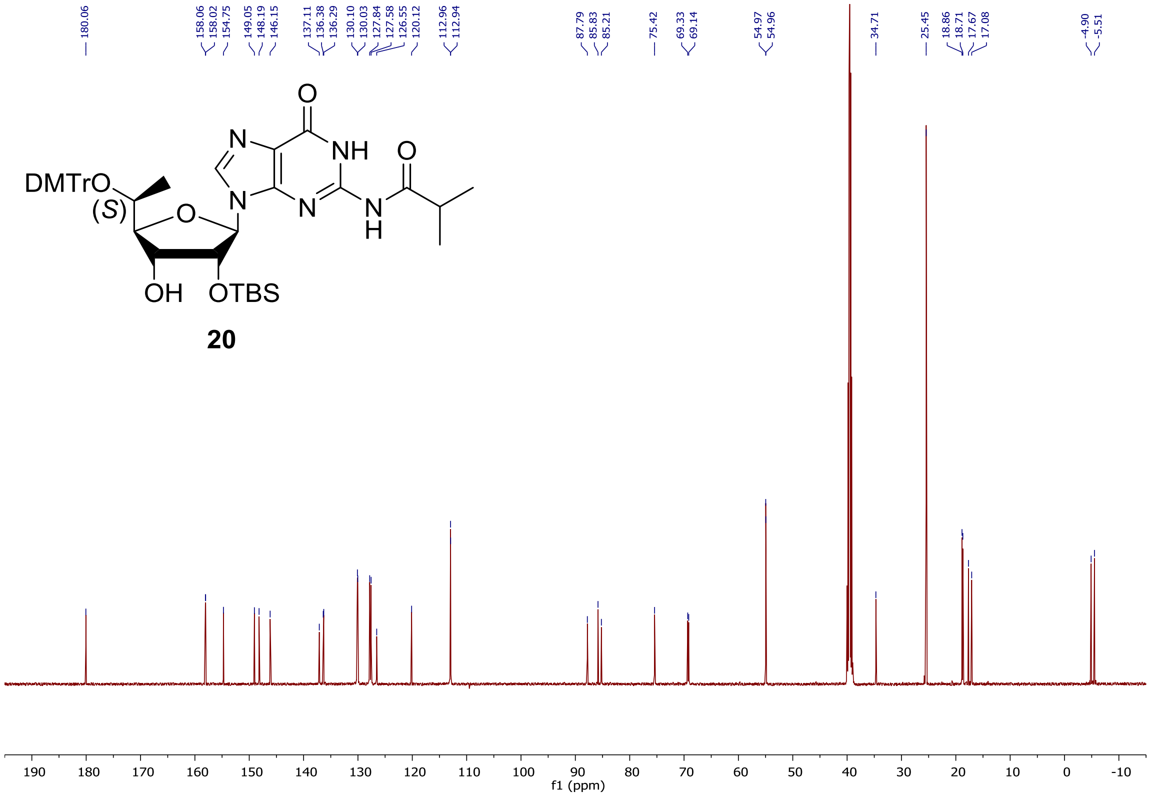

^{1}H NMR spectrum of compound 21 in $CDCl_3$

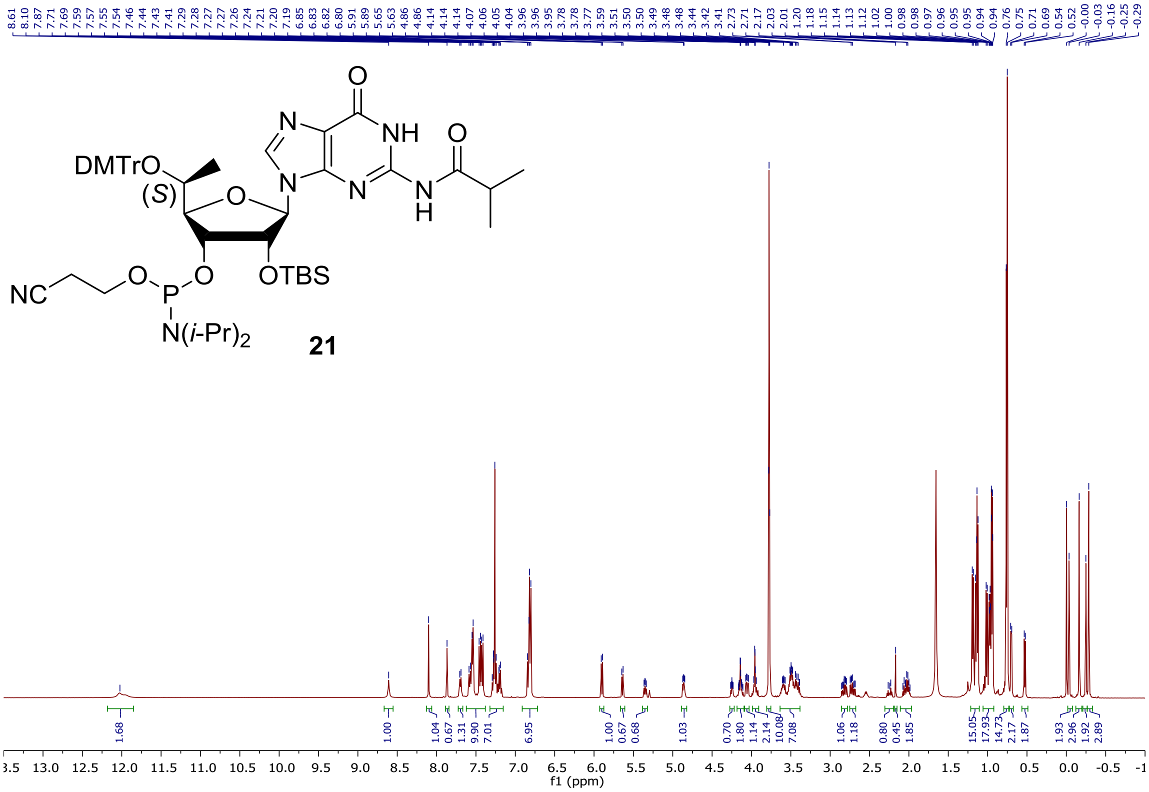

^{31}P NMR spectrum of compound 21 in $CDCl_3$

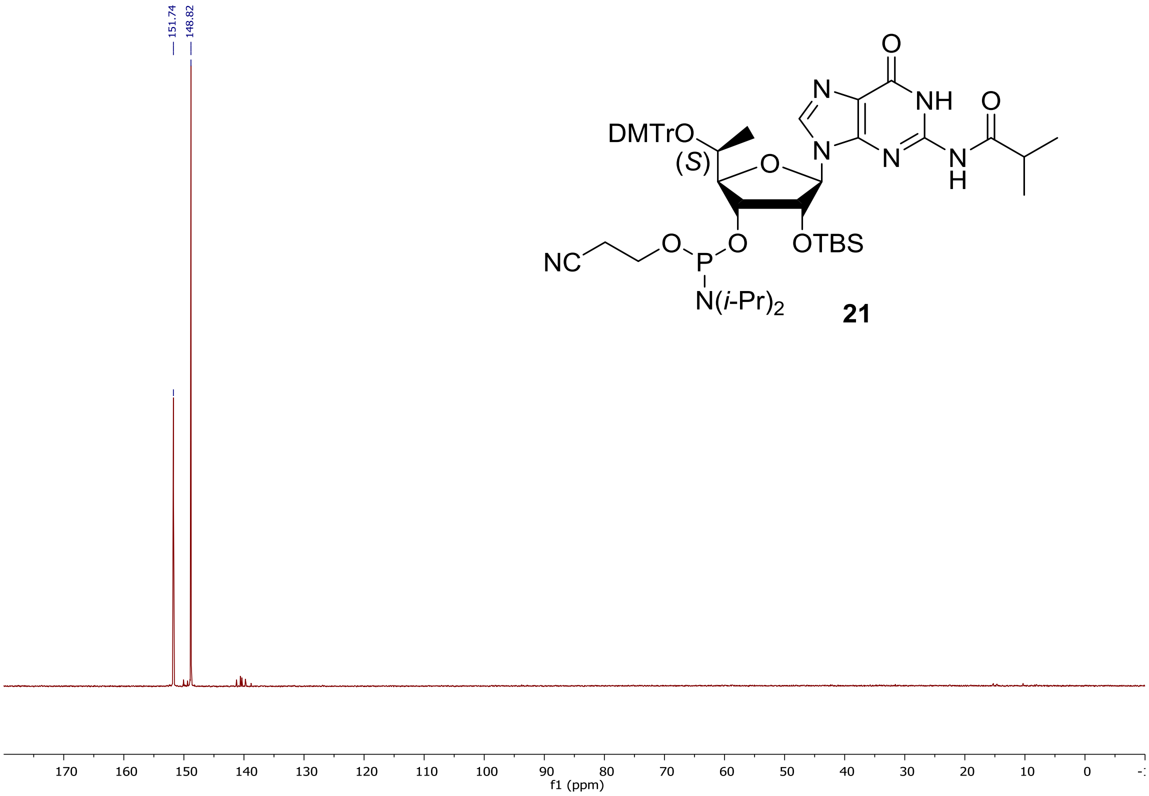

^{13}C NMR spectrum of compound 21 in $CDCl_3$

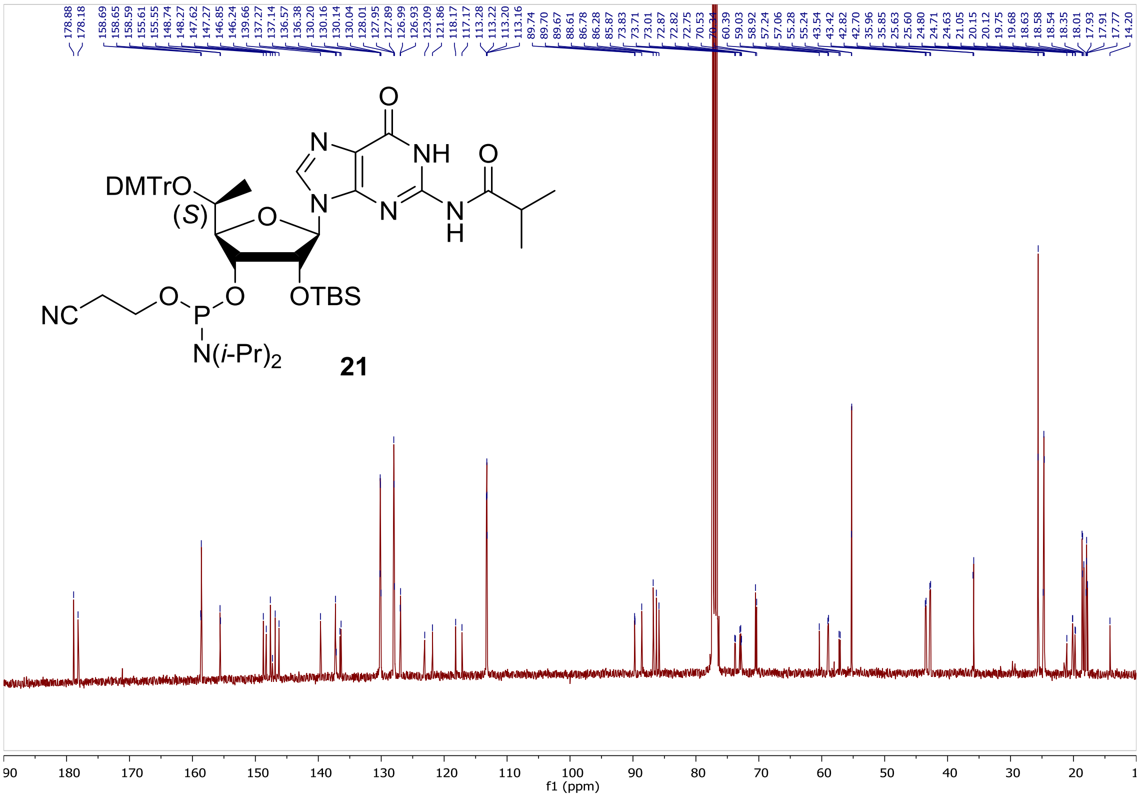

^{1}H NMR spectrum of compound 23 in DMSO-d6

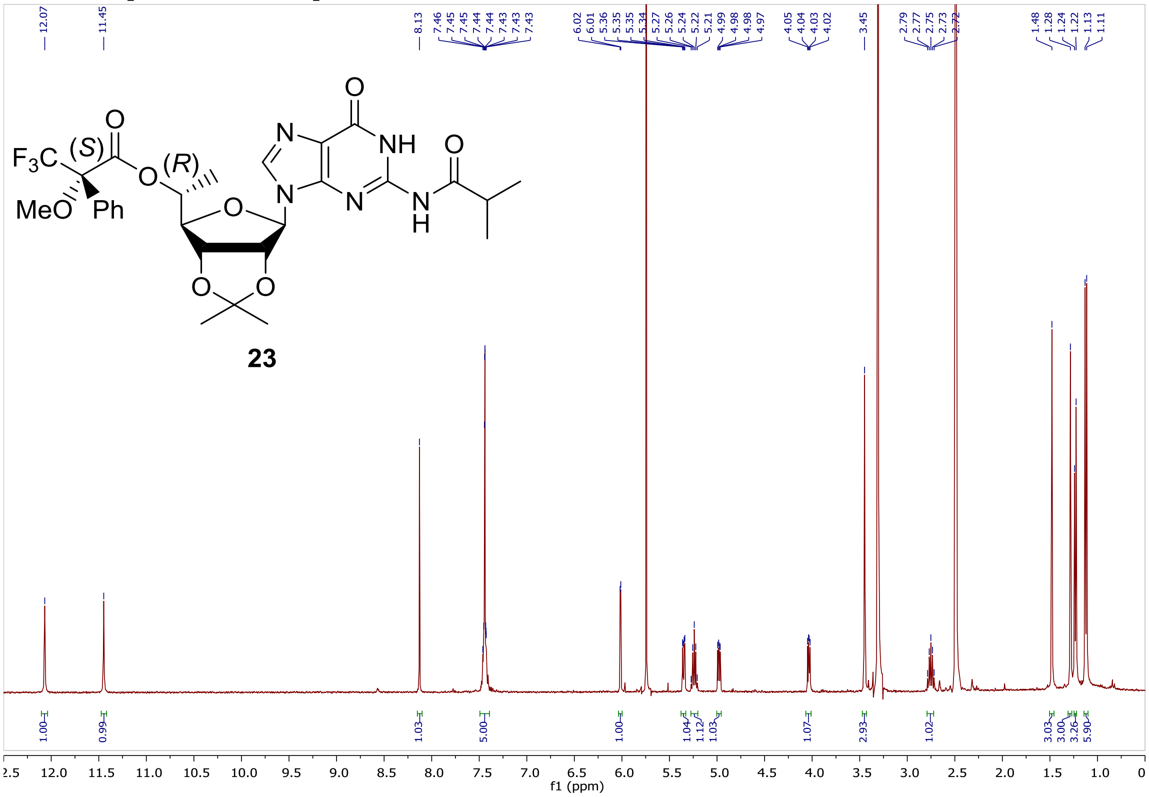

^{1}H NMR spectrum of compound 24 in DMSO-d6

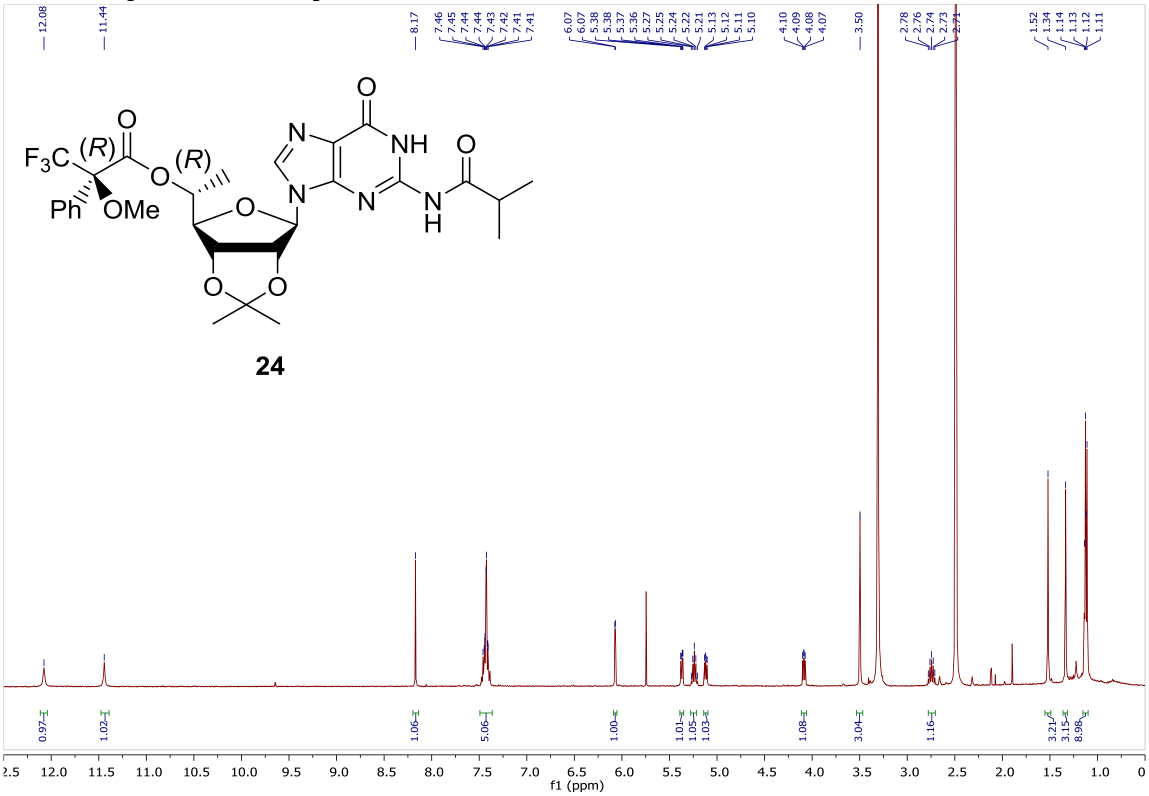

^{1}H NMR spectrum of compound 25 in DMSO-*d6*

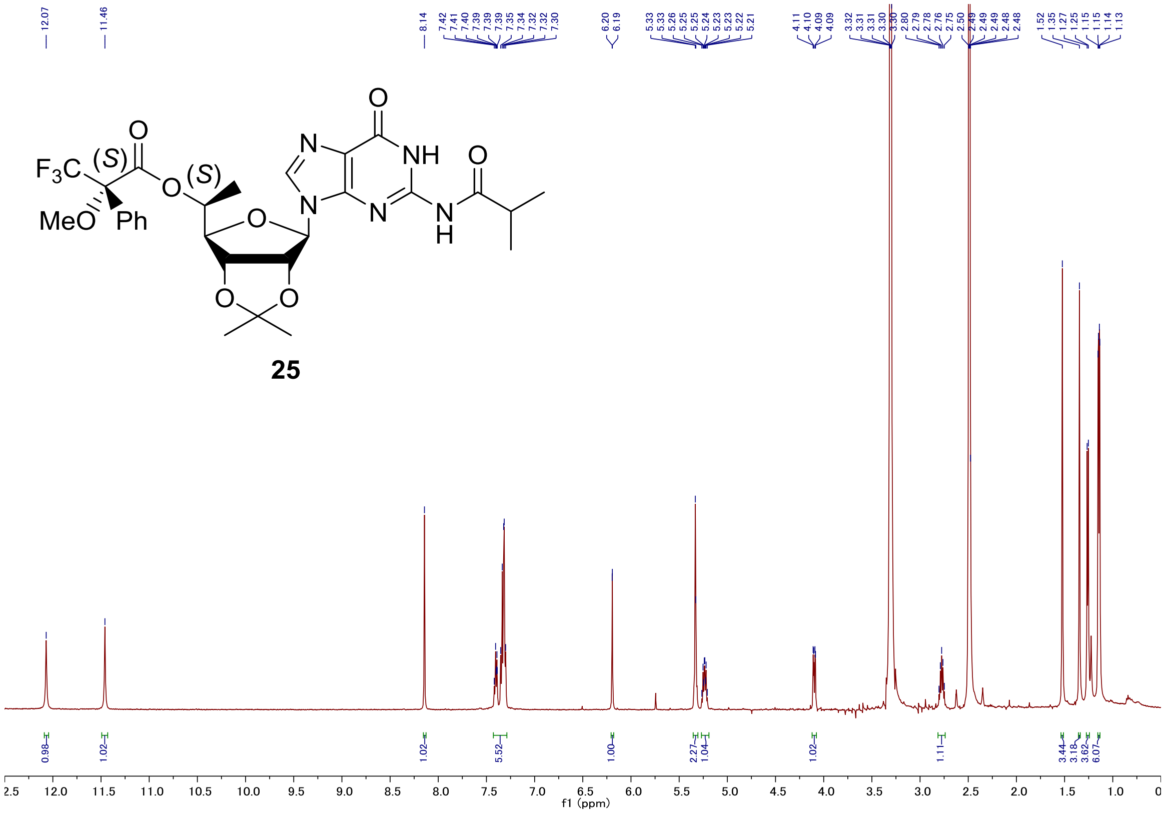

^{1}H NMR spectrum of compound 26 in DMSO-*d6*

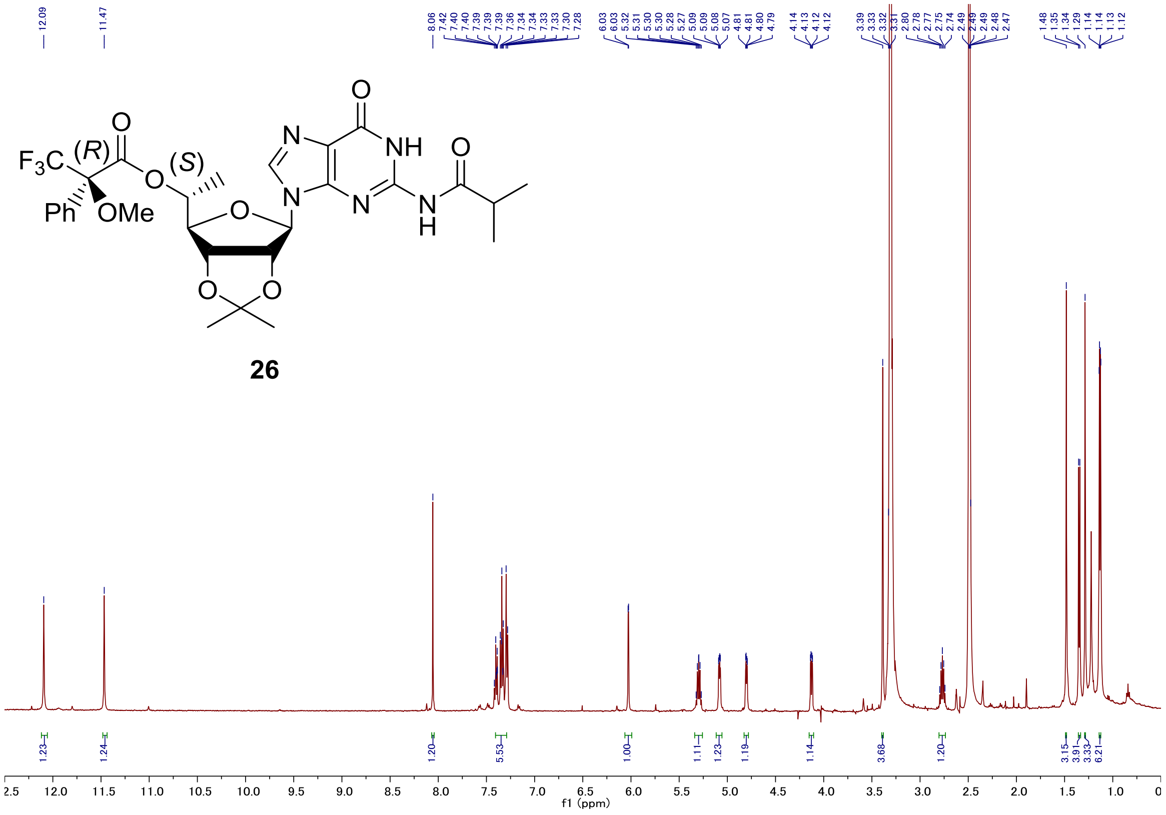

^{1}H NMR spectrum of compound 30 in D_2O

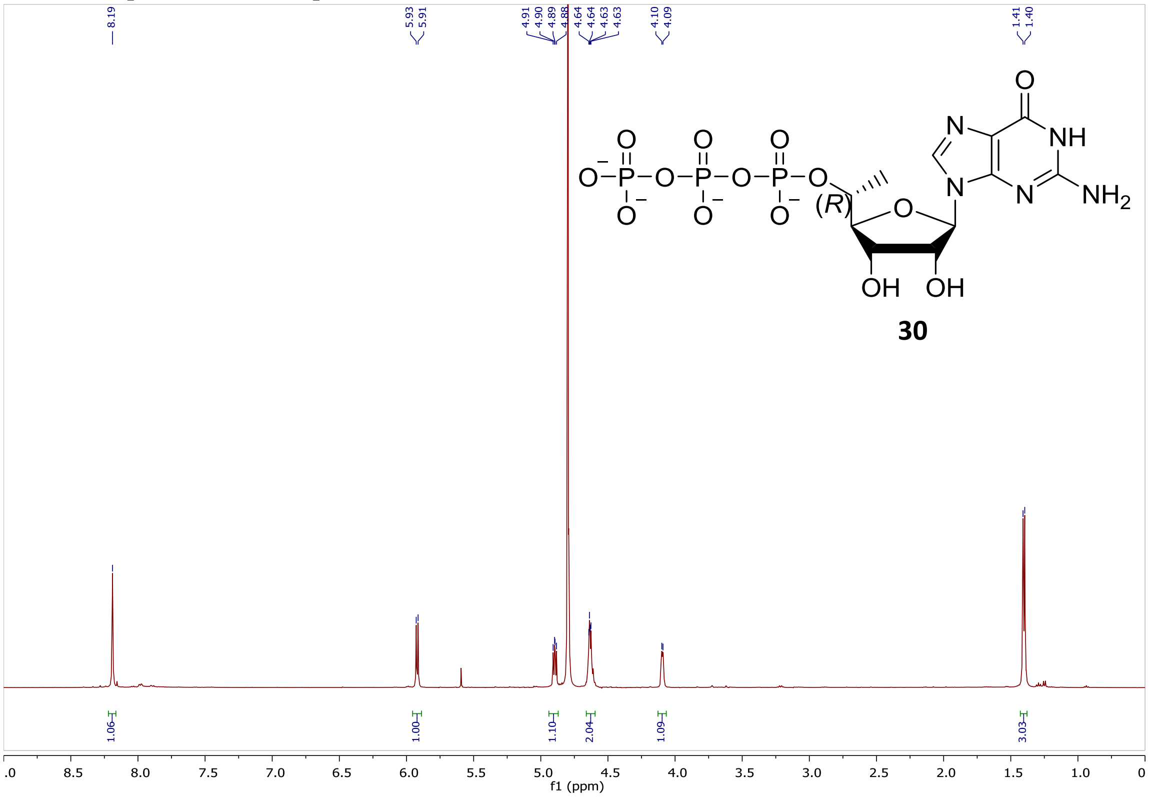

^{31}P NMR spectrum of compound 30 in D_2O

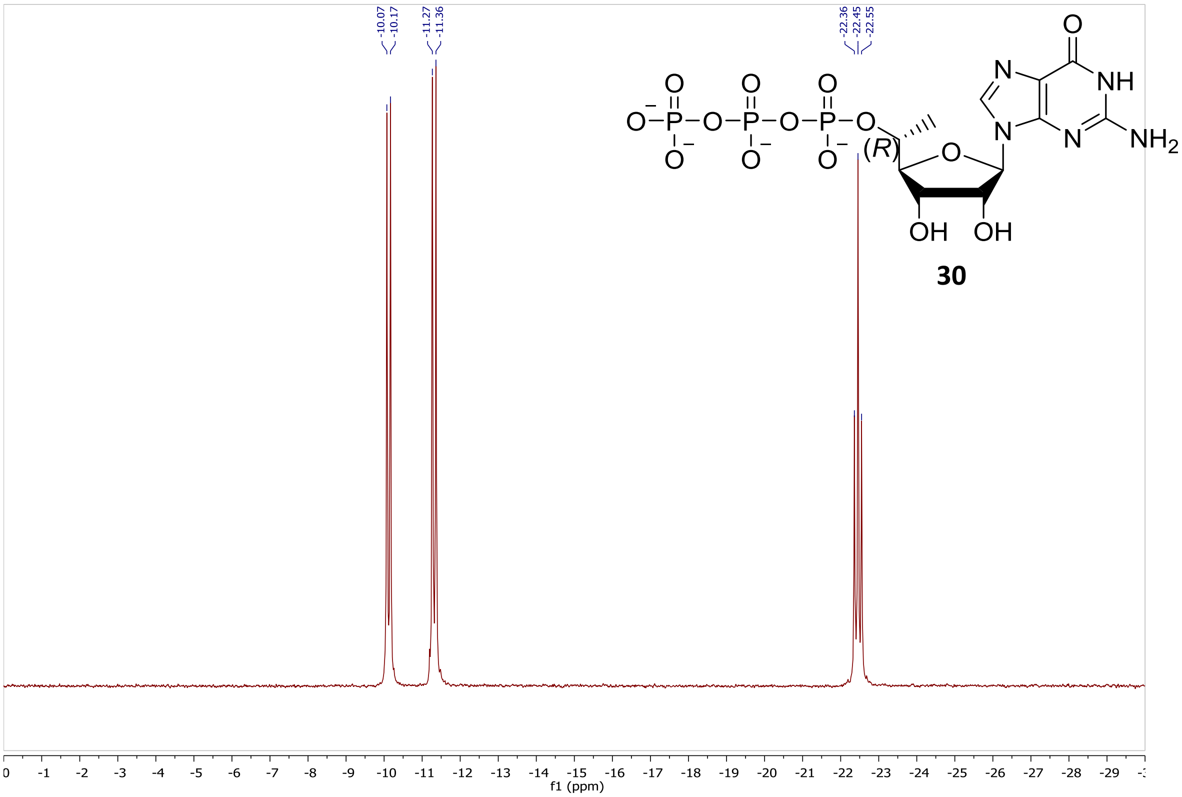

^{1}H NMR spectrum of compound 34 in D_2O

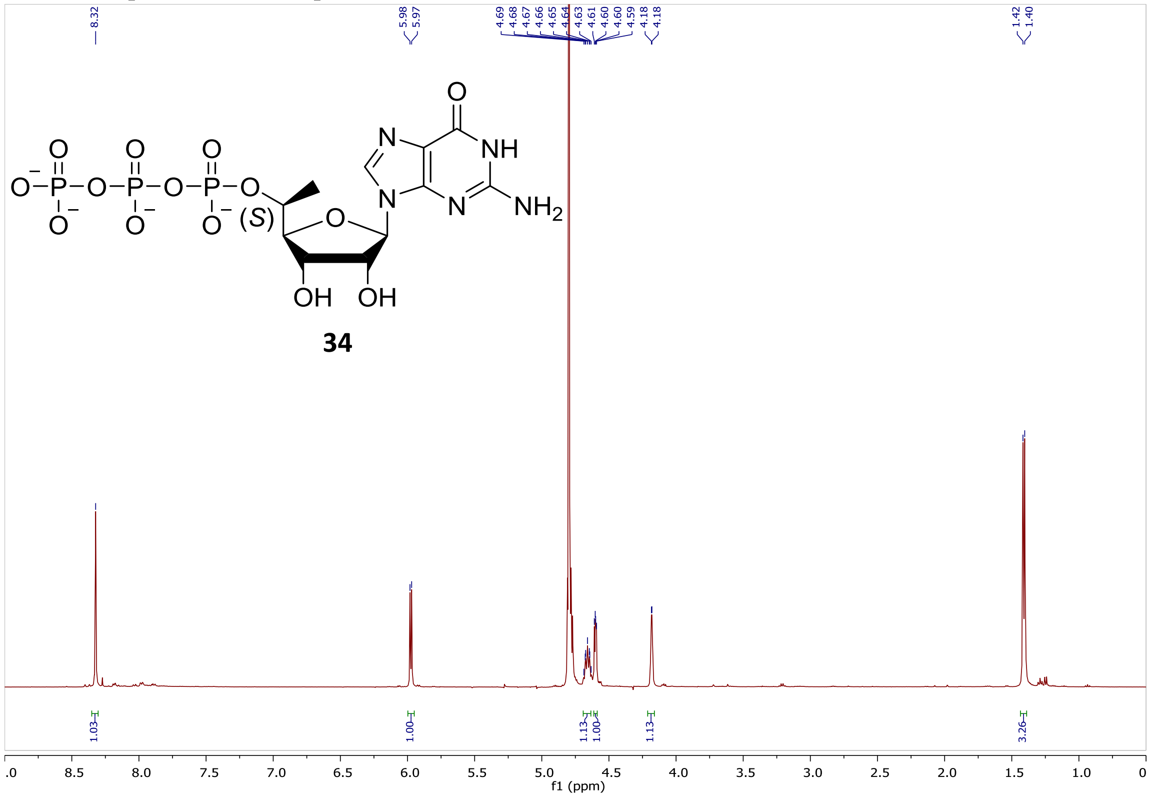

^{31}P NMR spectrum of compound 34 in D_2O

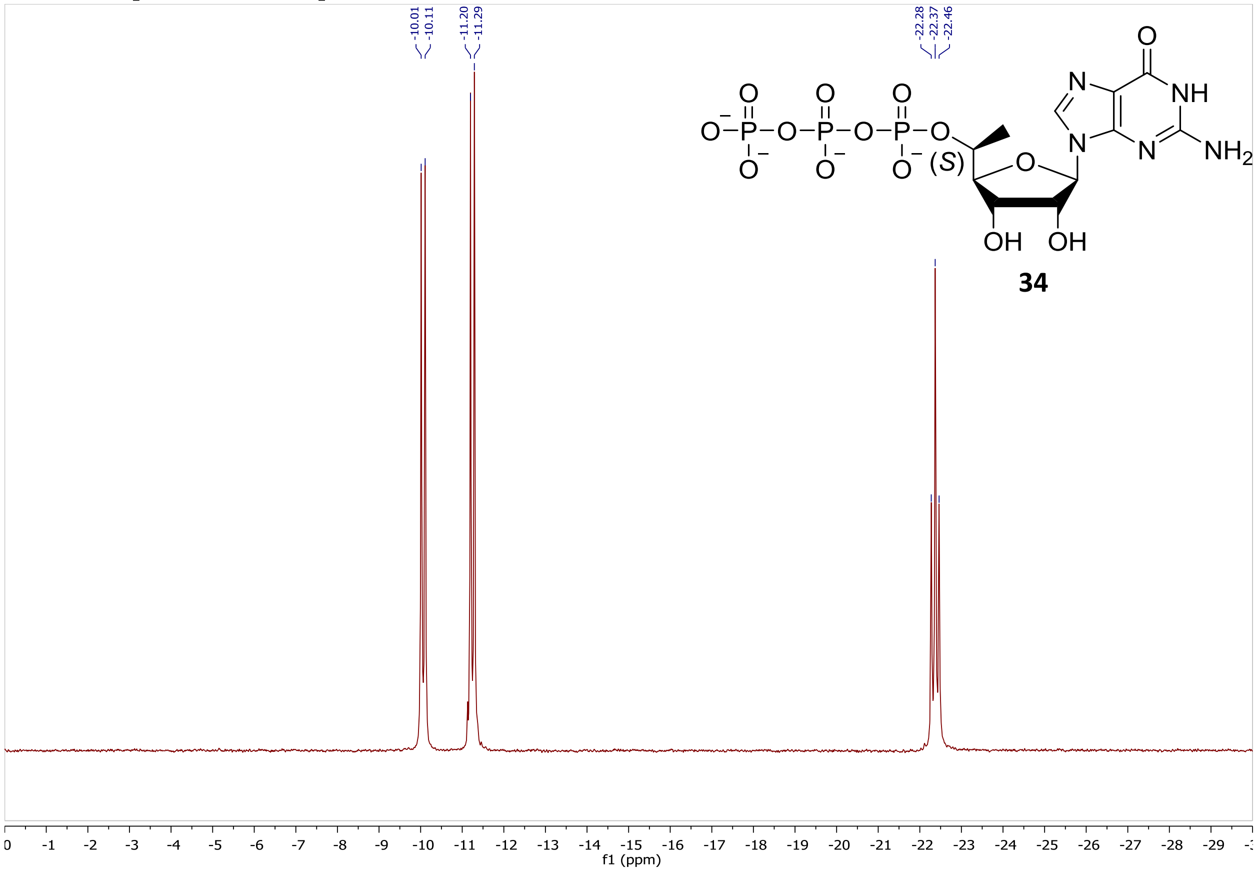

Supplement: gkaa750_Supplemental_File [file gkaa750_supplemental_file.pdf]
